# Whole-genome analysis uncovers loss of *blaZ* associated with carriage isolates belonging to methicillin-resistant *Staphylococcus aureus* (MRSA) clone ST5-VI in Cape Verde

**DOI:** 10.1016/j.jgar.2021.04.018

**Published:** 2021-09

**Authors:** Magdalena Wysocka, Tamar Monteiro, Carine de Pina, Deisy Gonçalves, Sandrine de Pina, Antonio Ludgero-Correia, Joao Moreno, Roxana Zamudio, Nada Almebairik, Laura J. Gray, Manish Pareek, David R. Jenkins, Marta Aires-de-Sousa, Herminia De Lencastre, Sandra Beleza, Isabel I. Araujo, Teresa Conceição, Marco R. Oggioni

**Affiliations:** a Department of Genetics and Genome Biology, University of Leicester, Leicester, UK; b Department of Molecular Biotechnology and Microbiology, Gdansk University of Technology, Gdańsk, Poland; c Universidade de Cabo Verde, Praia, Santiago, Cape Verde; d Department of Health Sciences, University of Leicester, Leicester, UK; e Department of Respiratory Sciences, University of Leicester, Leicester, UK; f Department of Clinical Microbiology, Leicester University Hospitals, NHS Trust, Leicester, UK; g Laboratory of Molecular Genetics, Instituto de Tecnologia Química e Biológica António Xavier (ITQB-NOVA), Universidade Nova de Lisboa, Oeiras, Portugal; h Escola Superior de Saúde da Cruz Vermelha Portuguesa, Lisbon, Portugal; i Laboratory of Microbiology & Infectious Diseases, The Rockefeller University, New York, NY, USA; j Dipartimento di Farmacia e Biotecnologie, Universita’ di Bologna, Bologna, Italy

**Keywords:** Genomic epidemiology, *Staphylococcus aureus*, Antibiotic resistance, Cape Verde, Methicillin-resistant, MRSA

## Abstract

**Objectives:**

Surveillance studies for *Staphylococcus aureus* carriage are a primary tool to survey the prevalence of methicillin-resistant *S. aureus* (MRSA) in the general population, patients and healthcare workers. We have previously reported *S. aureus* carriage in various African countries, including Cape Verde.

**Methods:**

Whole-genome sequences of 106 *S. aureus* isolates from Cape Verde were determined.

**Results:**

*Staphylococcus aureus* carriage isolates in Cape Verde show high genetic variability, with the detection of 27 sequence types (STs) and three primary genetic clusters associated with ST152, ST15 and ST5. One transmission event with less than eight core-genome single nucleotide polymorphisms (cgSNP) differences was detected among the ST5-VI MRSA lineage. Genetic analysis confirmed the phenotypic resistance and allowed the identification of six independent events of plasmid or transposon loss associated with the deletion of *blaZ* in nine isolates. In the four ST5 MRSA isolates, loss of the *blaZ* plasmid coincided with the acquisition of SCC*mec* type VI and an unusual penicillin phenotype with a minimum inhibitory concentration (MIC) at the breakpoint, indicating an adaptation trend in this endemic lineage. Similar events of *blaZ* plasmid loss, with concomitant acquisition SCC*mec* elements, were detected among ST5 isolates from different geographical origins.

**Conclusion:**

Overall, the genome data allowed to place isolates in a phylogenetic context and to identify different *blaZ* gene deletions associated with plasmid or transposon loss. Genomic analysis unveiled adaptation and evolution trends, namely among emerging MRSA lineages in the country, which deserve additional consideration in the design of future infection control protocols.

## Highlights

• One of the first whole genome analyses of *Staphylococcus aureus* carriage isolates in an African country.
• Genome data allowed to place *S. aureus* isolates from Cape Verde in a phylogenetic context.
• Loss of *blaZ*-carrying plasmids and transposons is not rare, which is also evident in other international MRSA isolates.
• Steady increase in antimicrobial drug resistance in Cape Verde.
• Data provide genomic information for the design of intervention measures to decrease antimicrobial resistance.

## 1 Introduction

*Staphylococcus aureus* is a commensal bacterium and a leading causative organism of healthcare and community-associated infections, with a high frequency of methicillin-resistant strains across different geographic regions [1], [2], [3]. *Staphylococcus aureus* poses a serious public-health burden worldwide [4,5], yet little is known about the population structure and dynamics of this pathogen in clinical settings in Africa, namely in Portuguese-speaking African countries (PALOP) [6], [7], [8], [9].

According to the European Antimicrobial Resistance Surveillance Network (EARS-Net) report from 2019 [10], Portugal has one of the highest prevalences of methicillin-resistant *S. aureus* (MRSA) in Europe (38.1%), with nosocomial invasive isolates exceeding the European mean of 16.4%. The close sociodemographic and economic relationships between Portugal and the PALOP countries may affect the clonal types circulating in these countries. A previous study comparing the genetic backgrounds of MRSA and methicillin-susceptible *S. aureus* (MSSA) in Portugal showed some MSSA clonal overlapping between Portugal and Cape Verde [11,12].

The first *S. aureus* surveillance study conducted in Cape Verde showed an *S. aureus* nasal carriage rate of ~41% amongst patients and healthcare workers (HCWs), but no MRSA was detected [6,7]. More recently, a comprehensive overview showed a highly variable MRSA prevalence among PALOP countries, with a low nosocomial prevalence of MRSA in Cape Verde (6%) in 2015, due to a major MRSA clone (50%), ST5-VI, in addition to three major MSSA clones (ST15, ST508 and ST152) recognised as international clonal types [4].

Although the isolates were previously characterised for antimicrobial susceptibility, population structure and the presence of Panton–Valentine leukocidin (PVL) toxin [4,[6], [7], [8], [9]], to date no studies have examined differences in *S. aureus* isolates in Portuguese former colonies in Africa using whole-genome sequencing (WGS) data, which is essential in helping to define local and global public-health priorities and contributes to the global picture of *S. aureus* epidemiology.

In the current study, we aimed to fill this gap by determining the genome information of *S. aureus* nasal carriage isolates from patients and HCWs in two hospitals in Cape Verde in order to analyse the previous information in a phylogenetic context.

## 2 Materials and methods

### 2.1 Hospital setting and sampling

A collection of 106 *S. aureus* isolates, representing 63.1% (106/168 isolates) of the *S. aureus* collection from Cape Verde available at the Laboratory of Molecular Genetics collection at ITQB (Oeiras, Portugal), were selected for WGS. The collection included carriage isolates from HCWs and patients isolated in three distinct time periods (1997, 2013 and 2014) in the two main public hospitals of Cape Verde [4,6], namely Hospital Agostinho Neto (HAN), located in Praia, Santiago Island, and Hospital Baptista de Sousa (HBS), located in Mindelo, São Vicente Island, which receive patients from all other islands of Cape Verde (Supplementary Tables S1–S5). Oral informed consent from each participant or from their guardians in the case of children was obtained at the time of sampling. Isolates were selected to include the highest variability in terms of clonal types [previously defined by pulsed-field gel electrophoresis (PFGE)] and collection period, meaning that at least one isolate from each PFGE subtype identified in each time period was included [4,6]. Antimicrobial susceptibility data for the collection of isolates had been established previously (Supplementary Table S4) [4]. The results were re-interpreted according to the actual guidelines of the European Committee on Antimicrobial Susceptibility Testing (EUCAST) (Version 10, 2020) (http://www.eucast.org/).

Additionally, for comparative genomic analysis of the main sequence types (STs) identified, we included publicly available genomic data from 49 representative isolates of these STs (ST5, *n* = 14; ST72, *n* = 8; ST152, *n* = 13; and ST188, *n* = 14) recovered from geographic regions with migration or economic links to Cape Verde (Supplementary Table S6).

### 2.2 Isolate sequencing and data pre-processing

Genomic DNA was extracted using a DNeasy® Blood & Tissue Kit (QIAGEN, Germantown, MD, USA) according to the manufacturer's instructions. Illumina sequencing libraries with an average insert size of 451 bp were prepared in accordance with the manufacturer's protocol and were sequenced on an Illumina HiSeq X Ten platform (Wellcome Trust Sanger Centre, Hinxton, UK) with 150-bp paired-end reads. This resulted in an average of 2 777 314 reads per sample, corresponding to an average of >100 × coverage per genome. Adapter sequences were trimmed using Trimmomatic v.0.36 [13].

### 2.3 Genome assembly, annotation and alignment

The genomes were de novo assembled using SPAdes v.3.9.0 [14]. QUAST v.5.0.2 [15] was used to generate summary statistics for each assembly (Supplementary Table S1). Bacterial sequence reads were assembled into primary contigs, which were deposited in the NCBI database with BioProject accession number **PRJNA609700** (Supplementary Table S1). Genomes were annotated with Prokka v.1.11 [16] using default parameters and adding –addgenes and –usegenus options. Prokka output was used as the input for the pangenome pipeline Roary v.3.6.0 [17] using the default parameter values. Roary created an alignment of core genes and defined gene ‘presence/absence’ across the isolate collection. Using a custom-made pipeline, a multi-FASTA file was obtained for each target core gene, which were then gene-by-gene aligned using MUSCLE v.3.8.31 [18] and afterwards concatenated using a custom Python script.

### 2.4 Phylogenetic analysis

In silico multilocus sequence typing (MLST) was performed using a BLAST-based tool (https://github.com/tseemann/mlst) on de novo genome assemblies and the staphylococcal cassette chromosome *mec* (SCC*mec*) elements were identified using SCCmecFinder (https://cge.cbs.dtu.dk/services/SCCmecFinder-1.2/). STs identified from the genome sequences are given in Supplementary Table S1. The maximum-likelihood core-genome phylogenetic tree was constructed from the concatenated alignment core genes using the GTR replacement model with four discrete categories of Gamma in RAxML v.8.2.12 [19]. The ggtree R package v.1.15.6 [20] was used for visualisation, manipulation and annotation of the phylogenetic trees. The cluster was defined using rhierBAPS [21,22].

**Fig. 1:**
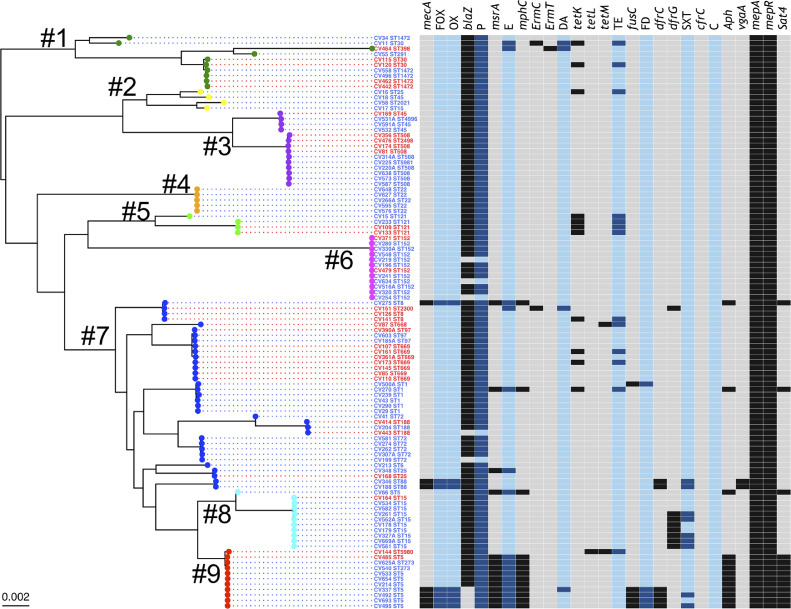
*Staphylococcus aureus* core-genome phylogenetic tree. A maximum-likelihood phylogenetic tree was constructed using 2014 core genes with the genome sequences of 106 Cape Verdean *S. aureus* isolates. The cluster numbers (#1–#9) are labelled on the phylogenetic tree and the colour of the circle in the external node is linked to their cluster (dark green, cluster no. 1; yellow, cluster no. 2; purple, cluster no. 3; orange, cluster no. 4; light green, cluster no. 5; pink, cluster no. 6; dark blue, cluster no. 7; light blue, cluster no. 8; and red, cluster no. 9). The origin of the isolates is distinguished by font colours in the tree: blue, Hospital Agostinho Neto (HAN) in Praia; and red, Hospital Baptista de Sousa (HBS) in Mindelo. The sequence type (ST) is indicated for each isolate, following the isolate name. In the heatmap, the presence/absence profile of the genotype for 13 genes encoding for resistance determinants (black, present; grey, absent) and the phenotype of antibiotic susceptibility testing for six antibiotics (dark blue, resistant; and light blue, susceptible) is indicated. FOX, cefoxitin; OX, oxacillin; P, penicillin; E, erythromycin; DA, clindamycin; TE, tetracycline; FD, fusidic acid; SXT, trimethoprim/sulfamethoxazole; C, chloramphenicol.

### 2.5 Identification of single nucleotide polymorphisms (SNPs)

The SNP pairwise differences matrix was obtained from the previous gene-by-gene alignment using snp-dists v.0.6.3 (https://github.com/tseemann/snp-dists). SNPs in the core-genome alignment were identified by aligning the short-read data from each isolate against the reference (GenBank accession no. **NC_007795**) using Snippy v. 3.1 (https://github.com/tseemann/snippy). SNPs in the core-genome alignment for the major STs were identified against reference genomes of the same ST, selected, whenever possible, from published *S. aureus* genomes isolated in a related geographic region at the same time (GenBank accession no. **BA000017.4** for ST5; BioSample accession no. **SAMEA3464938** for ST72; GenBank accession no. **LFOH01000000** for ST152; and GenBank accession no. **JFFV01000000** for ST188). The analysis pipeline was as follows: map the paired-end reads of each strain to the reference genome, call variants and obtain a variant annotation and the effects of variants on gene prediction using SnpEff tool v.4.3t (http://snpeff.sourceforge.net) [23].

### 2.6 Virulence factors, antibiotic resistance genes and plasmid analysis

The ABRicate tool was used to identify antibiotic resistance or virulence genes by running local assemblies [24], [25], [26]. Gene detection was performed in the genomes in the study using BLAST with a cut-off of 95% identity and length. Plasmid replicon types were identified using PlasmidFinder v.2.0.1 [27].

## 3 Results

### 3.1 Clonality and genetic diversity of Staphylococcus aureus in Cape Verde

We investigated the genome epidemiology of 106 *S. aureus* carriage isolates from two hospitals in Cape Verde collected from HCWs and patients (Supplementary Table S1) [4,6]. We performed a whole-genome phylogenetic analysis of these genomes using 2014 core genes to investigate the population structure of *S. aureus* in our study (Fig. 1). The genomic diversity of our collection is evidenced by the large number of accessory genes identified in the pangenome analysis (Supplementary Fig. S1). A total of 27 STs were represented among the 106 isolates. Five major STs, accounting for 44.3% (47/106) of the isolates, included ST152 (12 isolates), ST15 (11 isolates), ST5 (9 isolates), ST508 (8 isolates) and ST669 (7 isolates). Moreover, ST669 (7 isolates) was the most prevalent lineage in HBS, while ST152 and ST15 (with 11 and 10 isolates, respectively) were the major lineages in HAN (Fig. 1; Supplementary Tables S1 and S2). Two isolates had new STs: CV144 was ST5980, a single-locus variant (SLV) of ST5; and CV225 was ST5981, a SLV of ST508. Three isolates were characterised by STs reported for the first time in Africa (ST2300, ST2498 and ST4996 being, respectively, SLVs of ST2021, ST508 and ST45). Some of the isolates belonged to STs generally connected with livestock-associated strains, which included CV464 of ST398 [28,29] and CV185A, CV390A and CV603 of ST97 [30].

### 3.2 Single nucleotide polymorphisms (SNP) diversity of core genes and core genomes

**Fig. 2:**
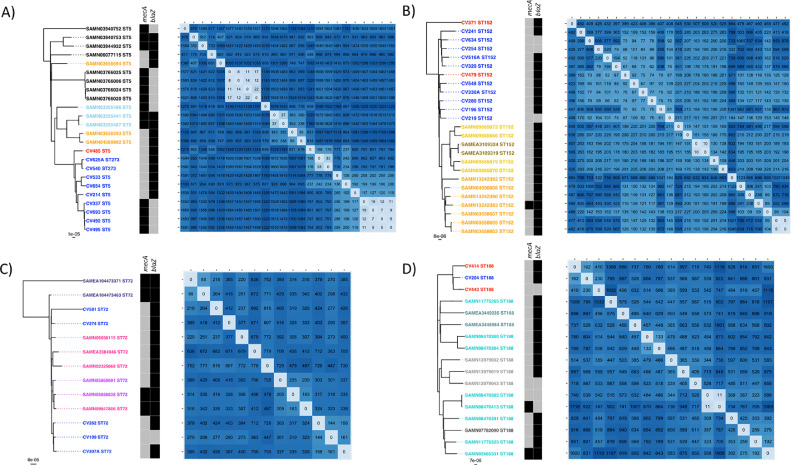
Maximum-likelihood phylogenetic trees based on core genes of the four most prevalent sequence types (ST5, ST152, ST72 and ST188), including publicly available genomes. Panels A through D show, respectively, phylogenetic trees of ST5, ST152, ST72 and ST188. To the right of the trees is the respective matrix of the core-genome single nucleotide polymorphism (cgSNP) differences determined against the reference genome from the same ST. The origin of the isolates is distinguished by their font colours in the tree. Colour codes: (A–D) royal blue, Hospital Agostinho Neto (HAN) in Praia; red, Hospital Baptista de Sousa (HBS) in Mindelo; (A) orange, Ghana; light blue, Portugal; and black, Brazil; (B) dark gold, Tanzania; light gold, Kenya; and orange, Ghana; (C) dark blue, Denmark; dark orchid, Spain; and pink, USA; and (D) dark turquoise, China; light turquoise, Thailand; dark grey, Argentina; and light grey, Colombia. In the heatmap, the presence/absence of the *mecA* and *blaZ* genes (black, present; grey, absent) is indicated.

The genomes were compared with the reference strain NCTC8325 to identify core-genome SNPs (cgSNPs). The isolates differed from the reference genome [31] by an average of 26 076 SNPs, which highlights the considerable genetic diversity of the study collection (Fig. 1). In our collection, we identified nine clusters through rhierBAPS (Fig. 1). To determine a possible local evolution or independent introductions of the most prevalent STs detected in Cape Verde, we included in our phylogenetic analysis publicly available genomes of isolates belonging to the same STs and with related geographical origin (Supplementary Fig. S2). When generating ST-specific core-genome trees (defined against a reference genome of that specific ST), our data showed that in the case of ST5, ST152 and ST188, the isolates from Cape Verde represented single locally circulating clones, while for ST72 multiple independent clones were present (Fig. 2).

To check clusters or related isolates (Supplementary Table S6) for evidence of recent transmission events, we determined the ST-specific cgSNP differences in all clusters and selected clusters for further analysis considering pairwise differences of <40 SNPs [32], [33], [34]. The tightest cluster corresponded to isolates from the major MRSA lineage identified in Cape Verde (ST5-VI), which showed only 5–19 cgSNPs (Fig. 2A). This ST5-VI cluster occurred exclusively among HCWs, sampled in different periods: one HCW was sampled in 2013 (CV337) and was resampled in 2014 (CV495 and CV693), and another HCW from the same ward was sampled in 2014 (CV492), showing an extended carriage period of ST5-VI and a transmission event. All other clusters showed differences of ≥53 cgSNPs.

**Fig. 4:**
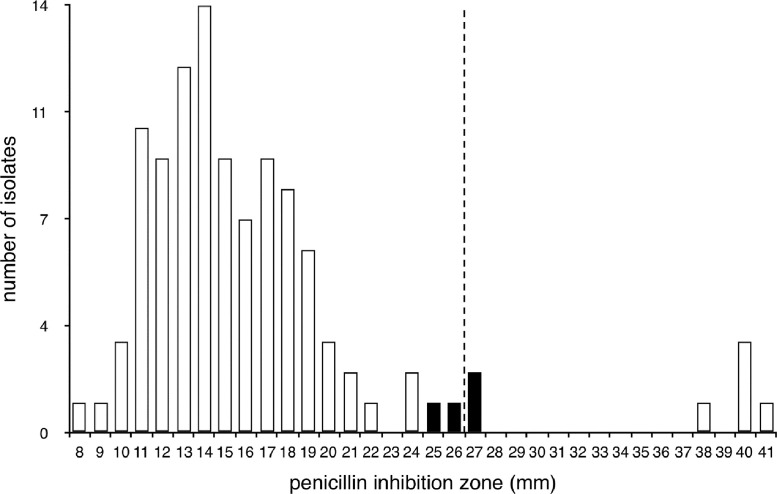
Distribution of penicillin inhibition zone diameters determined by disk diffusion. The diameter of inhibition by a 10 μg penicillin disk was plotted for all isolates. The ST5-VI MRSA isolates with a penicillin phenotype with zone diameters near the breakpoint are shown in black filled bars, with all other isolates represented as empty bars. The cut-off for resistance is plotted as a dotted line, with resistance on the left side and susceptible on the right side. ST, sequence type; MRSA, methicillin-resistant *Staphylococcus aureus*.

### 3.3 Virulence and antimicrobial resistance genes

Virulence gene analysis showed a 28.3% (*n* = 30) prevalence of the PVL genes *lukF* and *lukS*, in agreement with previous PCR data [4,7]. The toxic shock syndrome toxin 1 (TSST-1) gene was detected in 10.4% (*n* = 11) of the isolates, all MSSA. Many virulence genes were associated with specific STs, and the highest number of virulence genes were carried by the MSSA lineages ST1, ST72 and ST669 (Supplementary Fig. S3).

According both to antibiotic susceptibility testing and genome analysis (Supplementary Tables S3 and S4), 6.6% (*n* = 7) of the isolates were classified as MRSA. ST5 isolates carried SCC*mec* type VI, while ST8 and two ST88 isolates carried SCC*mec* type IVa element. Overall, we found perfect phenotype-to-genotype correlation for antibiotic susceptibility data, with all seven MRSA isolates carrying the *mecA* gene (Fig. 1). We observed a higher frequency of tetracycline resistance in Mindelo (25%) compared with the hospital in Praia (6.8%), and all of the tetracycline-resistant isolates were MSSA.

### 3.4 Phylogenetics and loss of penicillin resistance

**Fig. 3:**
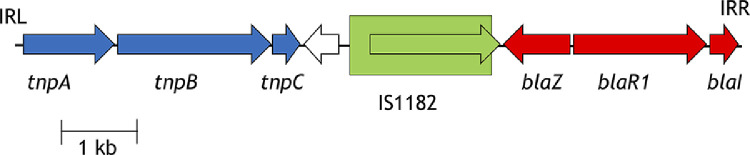
β-Lactamase transposon Tn*6720* and penicillin susceptibility. Tn*6720* is a 9561-bp composite transposon of ST72 *Staphylococcus aureus* and is mobilised by the three Tn*554* transposases (*tnpABC*, in blue). Tn*6720* also carries the β-lactamase module with *blaZ, blaR1* and *blaI* (red), a YolD-like family protein gene (white) and an IS*1182*-like insertion sequence (green; element boxed, transposase arrow). The inverted repeats IRL (TTATATATA) and IRR (TTATATGTA) (reverse complement) are shown at the border of the element. Tn*6720* was identified in isolate CV307A NODE_5 position 103392–112952. ST, sequence type.

The *blaZ* gene was not detected in nine of the 106 *S. aureus* carriage isolates studied, including four ST5 isolates, three ST152 isolates, one ST72 isolate and one ST188 isolate (Figs 1 and 2; Supplementary Fig. S2). In each case, these *blaZ*-negative isolates were part of clusters of related isolates in which all carried *blaZ*, suggesting that these nine cases represented loss of *blaZ* (Fig. 2). In the case of ST188 (isolate CV443) and ST152 (CV219, CV254 and CV634), these four penicillin-susceptible isolates had lost the same 20.8-kb plasmid (identical to GenBank **CP021142.1**; query cover 100%; identity 99%) (Supplementary Table S5), while in the case of ST72, isolate CV199 had lost a 9.5-kb chromosomal element, a transposon now designated Tn*6720*. Tn*6720* contained *blaZ, blaR1, blaI*, IS*1182* and three Tn*554*-like transposases (Fig. 3) [35]. In the case of ST188, isolate CV443 had a different plasmid [rep24_1_rep(pWBG745)] than the other ST188 isolates, while the ST152 *blaZ*-negative isolates (CV219, CV254 and CV634) lost all plasmids, and the ST72 *blaZ*-negative strain (CV199) had no change in their plasmid profile (Supplementary Table S5). Construction of detailed ST-specific phylogenetic trees and SNP matrixes for ST152 and ST188 showed that all four events of plasmid loss (CV443, CV219, CV254 and CV634) were independent events. Regarding the cluster of the four ST5 *bla*Z-negative isolates (CV693, CV495, CV492 and CV337), although the isolates lost a 29.0-kb plasmid containing the *blaZ* gene (identical to GenBank **GQ900405.1**), they acquired an SCC*mec* element (Fig. 2A). The ST5 *blaZ*-negative isolates lost the only plasmid they had, the rep5_4_rep(SAP047A) (Supplementary Table S5). The SCC*mec* element in these ST5 isolates was identified as a SCC*mec* type VI with delta-*mecR* and no *mecI*. Similar events of loss of *blaZ*-carrying plasmids and concomitant acquisition of SCC*mec* elements were also detected in the few international ST5 and ST188 isolates included in the phylogenetic analysis (Fig. 2).

In the *blaZ*-negative ST5 isolates, the presence of penicillin-binding protein 2A (PBP2A) encoded by the *mecA* gene on the SCC*mec* element conferred a penicillin phenotype with inhibition zones that fell between typically resistant and susceptible isolates (Fig. 4). To identify the rationale for this unusual resistance profile to penicillin (Fig. 4) in the absence of the *blaZ* gene, we checked in the cgSNPs that could distinguish the four ST5-VI MRSA isolates from all other ST5 isolates. Of the 53 cgSNPs, only 3, determining premature stop codons, are regarded a priori as having a high likelihood of impact on the phenotype: the bifunctional ornithine acetyltransferase ArgJ (SAOUHSC_00148); the MutS domain V protein (SAOUHSC_02276); and the sigma B regulation protein RsbU (SAOUHSC_02301). Of these, only the deletion of *rsbU* might be linked to the observed phenotype in our strains, since RsbU positively regulates the murein hydrolase genes *cid* and *lrg* [36]. In addition, the common ancestor of all isolates from this ST5 cluster of four highly related isolates (CV214) lost a 61.2-kb bacteriophage with high identity to staphylococcal phage tp310-2 [identity 28 843/29 708 (97%); accession **NC_009762.3**]. This documents at least three important genetic events in the accessory genome (plasmid loss, bacteriophage loss and SCC*mec* acquisition) of this MRSA ST5-VI clonal lineage (Supplementary Fig. S1). No compensatory cgSNPs could be identified associated with the major rearrangements of the accessory genome of this emerging clone.

## 4 Discussion

This work provides the phylogenetic information gained by WGS of *S. aureus* carriage isolates from two surveillance studies in patients and HCWs in 1997 and 2013–2014 in Cape Verde [4,6]. Genome-based sequence typing confirmed the classical MLST profiling of isolates performed previously on these isolates [4,7]. The combination of the two data sets showed that some STs are not homogeneously distributed over the islands, with ST669 being present in HBS only (mostly in 1997) [7] and ST5, ST15 and ST152 predominantly in HAN [4], while others are equally present in both settings, such as ST508 or ST1472 [4,7]. The great advantage of whole-genome phylogenetic analysis is that it provides additional genomic information to assess the relatedness of isolates within a given cluster of isolates, as for example within a single ST, and potentially predictions can be made on the timescale of transmission or the occurrence of outbreaks [37]. In addition, our data reveal substantial *S. aureus* clonal diversity present within the population. Although the majority of the isolates in our collection belonged to the ST152 or ST15 clonal lineages commonly found among *S. aureus* infections occurring in Africa [38], we also identified a significant number of unique isolates that belonged to other clonal lineages. When including additional *S. aureus* genome sequences of isolates from geographical areas with documented contact to Cape Verde, such as Portugal, Brazil, China or other African countries, we could not detect any direct dependence or clear phylogenetic relationship. But this might likely also be due to the limited number of genomes available for each specific ST and countries. Our data set contains the first reported isolates of ST2300 (double locus variant of ST8), ST2498 (SLV of ST508) and ST4996 (SLV of ST45) *S. aureus* in Africa, and multiple novel MLST clonal types that represent SLVs of ST508 and ST5 organisms.

The detection of β-lactam, macrolide and tetracycline resistance genes matches exactly with the susceptibility phenotypes reported previously [4,6]. Distribution of the *ermC* and *msrA* markers is in accordance with the well-described epidemiology of drug resistance markers in *S. aureus* [39]. The single *ermT*-positive isolate belonged to ST398, which corroborates the description of *ermT* as one of the markers defining the human clade of ST398 [40]. The average tetracycline resistance in these carriage isolates is lower than in clinical isolates from Cape Verde [41] as well as isolates from other countries of the region including São Tomé and Príncipe [41], [42], [43]. It should be noted that all our tetracycline resistance was detected in MSSA, which differs from the scenario in neighbouring countries [4]. This difference is reflected in the unusual high prevalence of *tetK* (*n* = 11) compared with *tet(M)* (*n* = 2) [44,45].

The *mecA* gene was detected in seven *S. aureus* isolates, of which four MRSA ST5-VI were recovered from HCWs, evidencing a recent transmission event and suggesting that HCWs could represent reservoirs of this MRSA lineage. A recent 5-year survey of susceptibility profiles in clinical *S. aureus* isolates in the two Cape Verde hospitals included in this work identified a significant increase of MRSA prevalence between 2013 and 2017, with most MRSA isolates being also erythromycin-resistant; however, the extent of MRSA ST5-VI clonal lineage could not be identified [41]. On the other hand, the apparent maintenance of erythromycin resistance suggests that this MRSA ST5-VI clonal lineage identified in carriage isolates could represent the precursor lineage for MRSA clones emerging in the clinical setting in Cape Verde.

An unusual feature in our data set was the high occurrence of *blaZ*-negative isolates within *blaZ*-resistant clusters of isolates. Based on the phylogenetic tree data, we could determine that all of the *blaZ*-negative isolates emerged due to loss of a *blaZ*-containing plasmid or chromosomal element. Importantly, we observe loss of *blaZ* plasmids not only in our Cape Verde data set but also in the genomes of isolates belonging to the same STs but recovered from other countries. This loss of *blaZ* in the MSSA population might be underestimated in the literature, as only genome phylogenetic data can confirm that *blaZ*-negative penicillin-susceptible isolates originated from penicillin-resistant ancestors by the loss of the plasmid or transposon. This loss of *blaZ* event was already reported in other successful clones in African countries [9] and elsewhere [46], as occurred in our ST5-VI MRSA cluster. Moreover, our data suggest that the rare penicillin phenotype with a minimum inhibitory concentration (MIC) at the breakpoint observed among these ST5-VI MRSA was due to a premature stop codon that prevents the production of the sigma B regulation protein RsbU, involved in the regulation of murein hydrolase activity [36]. On the other hand, since these strains contain the *mecA* gene, which encodes an altered penicillin-binding protein (PBP2A) conferring resistance to virtually all β-lactams, it is also possible that the low penicillin resistance phenotype detected is a result of a slower induction of *mecA* owing to the absence of *blaZ*, as it was already demonstrated that expression of *mecA* relies upon an intact *blaZ* system [47].

MSSA strains have a greater likelihood of producing toxins, such as PVL, than MRSA strains [48], which is consistent with our findings confirming the high occurrence of PVL-positive MSSA isolates in Cape Verde, as previously reported [4,7]. In addition, we identified the TSST-1 genetic determinant in 11 isolates. Although *tsst-1* has been commonly identified among MRSA strains, in our collection all *tsst-1*-positive isolates were MSSA [49].

WGS is an ideal tool to identify outbreaks and to trace transmission events in high resolution. Although the definition of an optimal cut-off for *S. aureus* strain relatedness has been controversial, we assumed the proposed cut-off of 40 cgSNPs [32], [33], [34], which allowed the identification of several clusters of related isolates. Nevertheless, ST-specific cgSNP analysis showed that only the ST5 cluster of isolates shared <40 SNP differences. The four ST5-VI MRSA isolates (three of which were from the same subject) collected in two different time periods (2013 and 2014) suggest extended carriage and one transmission event of this MRSA isolate, supporting the idea that HCWs can be reservoirs of MRSA in hospitals. The fact that these ST5-VI isolates differed by ≤8 SNPs raises the concern about the cut-off for the definition of outbreak isolates. Even taking into account the *S. aureus* mutation rate of 3.3 × 10^−6^ mutations per site per year [50], there is still no good cut-off to distinguish an outbreak [33,34]. Assuming a cut-off of 10 cgSNPs for defining an outbreak, as suggested for other pathogens as *Listeria monocytogenes*, might still be problematic since independent isolates and sequential isolates from the same host have been reported to show a similar number of SNPs [33,34]. In the case of *L. monocytogenes*, where tracing of foodborne outbreaks is of key importance, the initially defined cut-off at 10 core-genome MLST (cgMLST) differences [51] was even reduced to <7 cgMLST differences (equivalent to 7 cgSNPs) [52]. Based on our observation of the ST5-VI cluster, which included isolates from 2013 and 2014, we would propose the cut-off for definition of recent transmission to <6 SNPs, which is very similar to the <7 SNP cut-off defined for *L. monocytogenes* outbreaks. While WGS could give outstanding information in defining outbreaks, additional epidemiological evidence will still be needed for definitive confirmation of isolate relatedness in an outbreak situation.

## Competing interests

None declared.

## Appendix Supplementary materials

Image, application 1
