# Supplementary material for: Whole-genome analysis uncovers loss of blaZ associated with carriage isolates belonging to methicillin-resistant Staphylococcus aureus (MRSA) clone ST5-VI in Cape Verde
Source: J Glob Antimicrob Resist. 2021 Sep;26:77–83. doi: 10.1016/j.jgar.2021.04.018 (PMC8440226; doi:10.1016/j.jgar.2021.04.018)
Supplement: Supplementary file 1 [file mmc1.docx]

**SUPPLEMENTARY** **INFORMATION**

**Whole-genome analysis uncovers loss of *blaZ* associated to carriage isolates**

**belonging to MRSA clone ST5-VI in Cape Verde**

Magdalena Wysocka, Tamar Monteiro, Carine de Pina, Deisy Gonçalves, Sandrine de

Pina, Antonio Ludgero-Correia, Joao Moreno, Roxana Zamudio, Nada Almebairik,

Laura J Gray, Manish Pareek, David R Jenkins, Marta Aires De Sousa, Herminia De

Lencastre, Sandra Beleza, Isabel I Araujo, Teresa Conceição, and Marco R Oggioni

Index:

Figure S1: Pan-genome analysis of 106 *S. aureus* whole-genome sequences

Figure S2: Core-genome phylogenetic tree generated for 155 *S. aureus* isolates

Figure S3: Phylogenetic tree and virulence gene profile of 106 *S. aureus* strains

Table S1: Isolate metadata of *S. aureus* isolates from Cape Verde

Table S2: Virulence genes identified in *S. aureus* isolates from Cape Verde

Table S3: Antibiotic resistance genes identified in *S. aureus* isolates from Cape Verde

Table S4: Phenotypic antimicrobial susceptibilities of *S. aureus* isolates from Cape Verde

Table S5: Plasmid replicons types identified in *S. aureus* isolates from Cape Verde

Table S6: List of *S. aureus* genomes used for comparative analysis


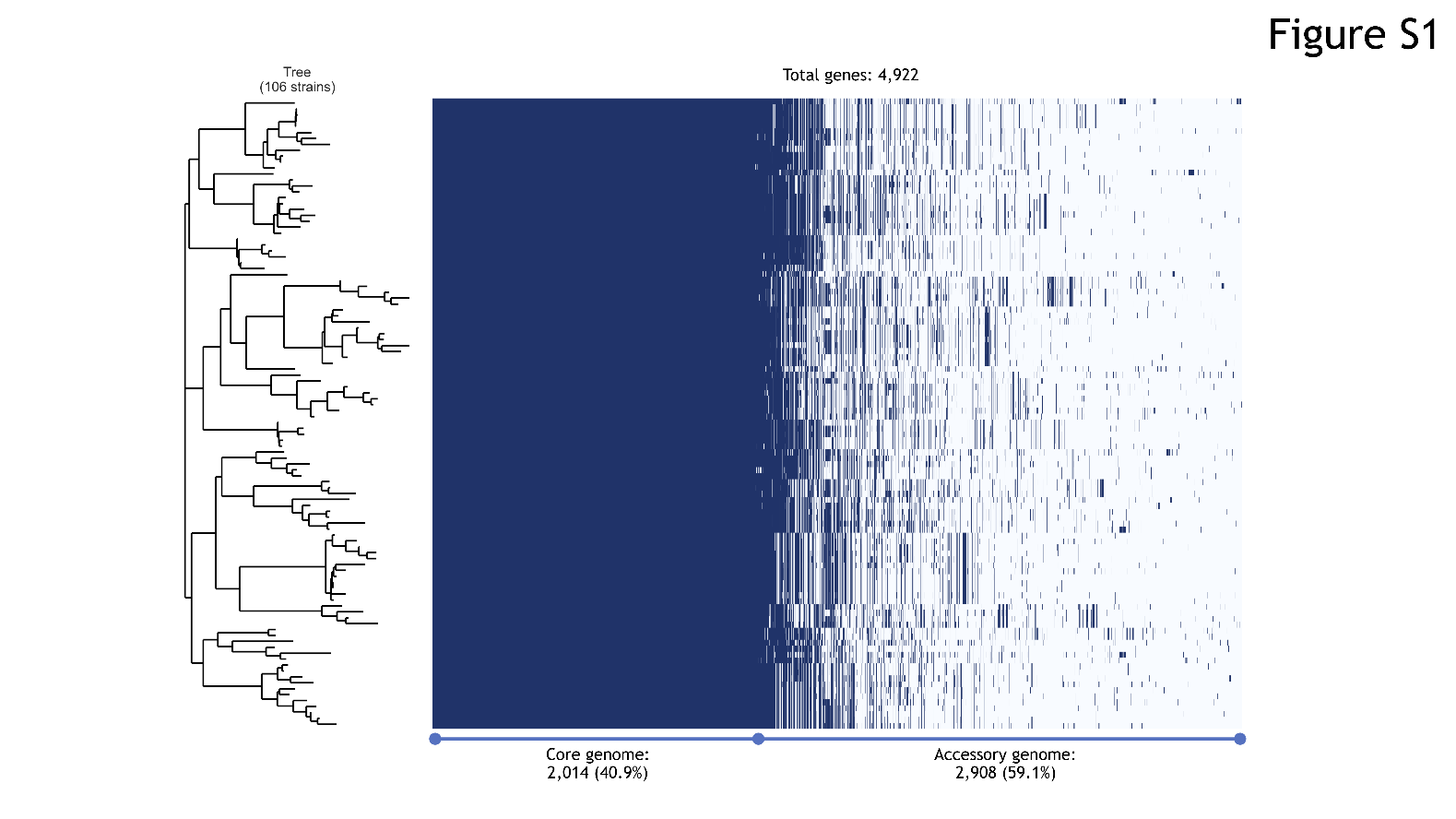


**Figure S1: Pan-genome analysis of 106 *S. aureus* whole-genome sequences.** The left panel shows a phylogenetic tree based on a concatenated core gene alignment. The right panel shows a gene possession matrix, with each row representing each strain’s gene content. Columns are ordered by the frequency of gene presence. The heatmap indicates presence (deep blue) or absence (white) of 2,908 accessory genes that are present in 5%-95% of genomes.


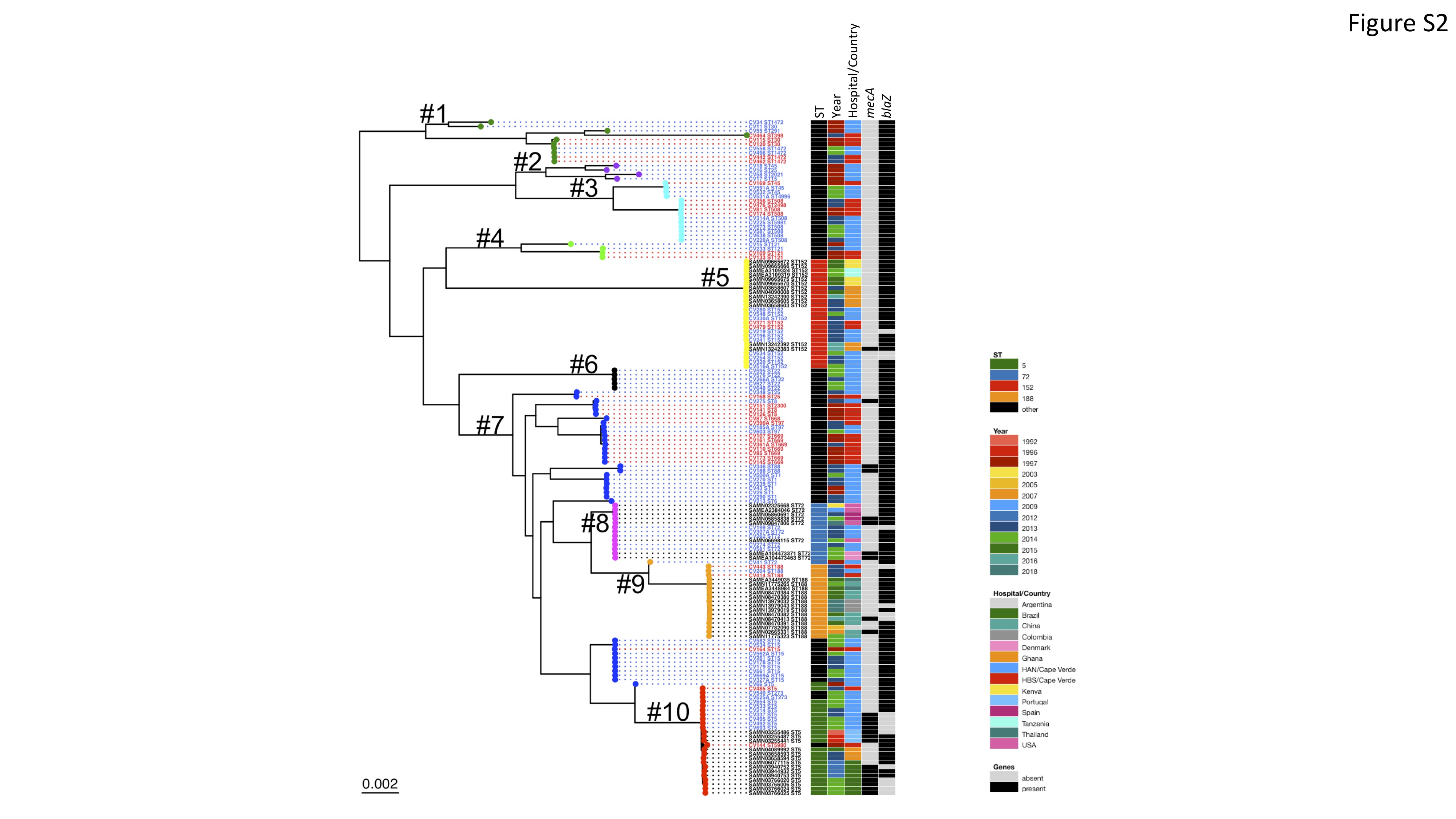


**Figure S2: Core-genome phylogenetic tree generated for 155 *S. aureus* isolates (106 from our collection and 49 from public database, as described in Table S6).** The cluster numbers (#1-#10) are labelled on the phylogenetic tree and the colour of the circle in the external node is linked to their cluster (green - cluster no. 1, purple - cluster no. 2, light blue - cluster no. 3, light green - cluster no. 4, yellow - cluster no. 5, black - cluster no. 6, dark blue - cluster no. 7, pink - cluster no. 8, orange - cluster no. 9, red - cluster no. 9). The origin of the isolates is distinguished by font colours in the tree: blue - Hospital Agostinho Neto (HAN) in Praia; red - Hospital Baptista de Sousa (HBS) in Mindelo; black – geographically diverse isolates. The sequence type (ST) is indicated for each isolate, following the isolate name. In the heatmap the year of isolation, the specific origin of the isolates and the presence/absence of the *mecA* and *blaZ* genes (black - present, grey – absent) are indicated.


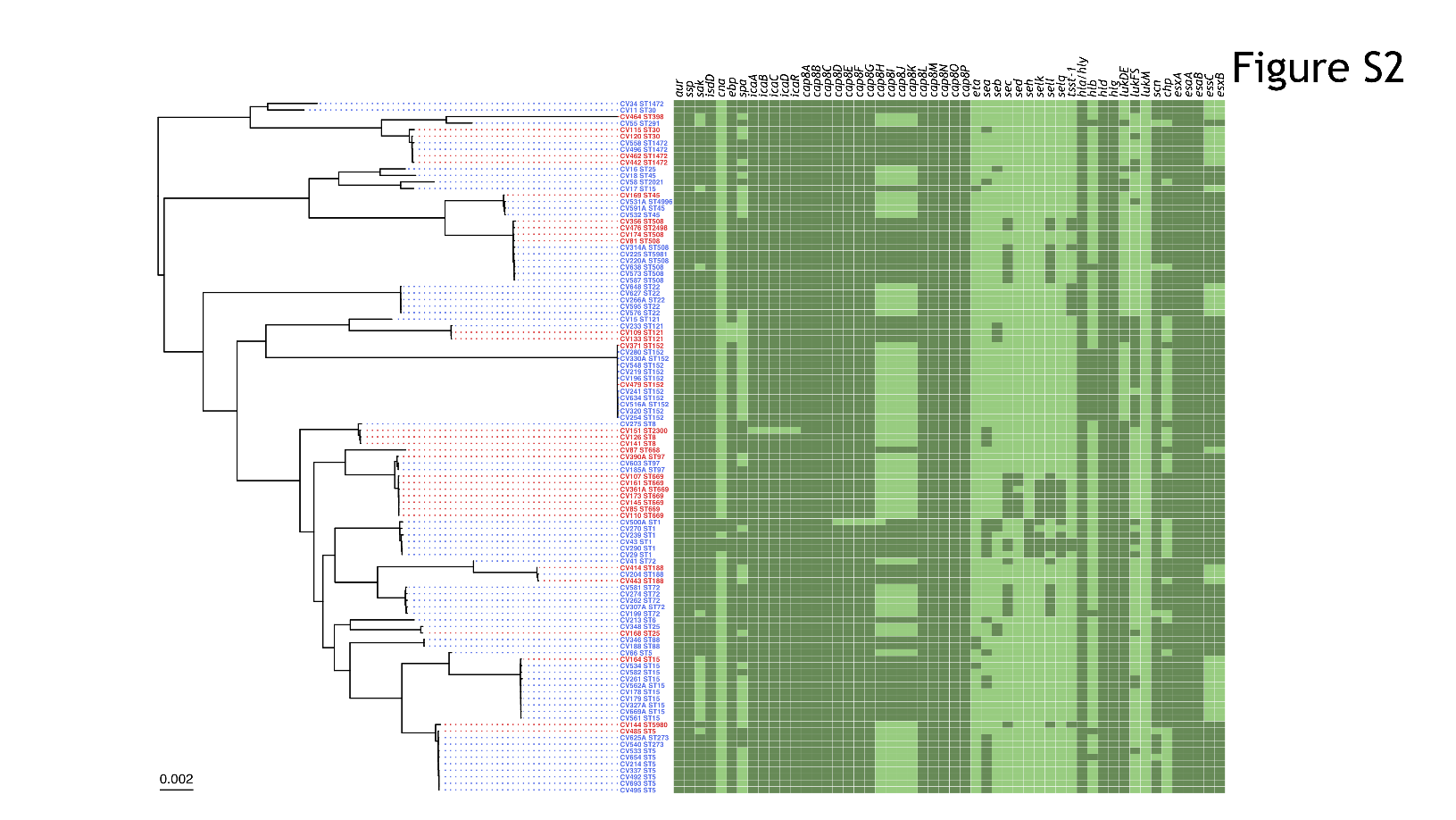


**Figure S3: Phylogenetic tree and virulence gene profile of 106 *S. aureus* strains.** A maximum likelihood tree was constructed using 2,014 core genes from a collection of 106 Cape Verdean isolates. The origin of the isolates is distinguished by font colours in the tree: blue - Hospital Agostinho Neto (HAN) in Praia; red - Hospital Baptista de Sousa (HBS) in Mindelo. The sequence type (ST) is indicated for each isolate, following the isolate name. The presence of a given virulence factor is shown in dark green, while absence is in light green. For a more detailed overview of the single genomes’ profiles, see Table S2.

Table S1: Isolate metadata of *S. aureus* isolates from Cape Verde

| **Assembly** | **Biosample accession** | **Country** | **Hospital^a^** | **Year** | **PA/HCW^b^** | **Source** | **MRSA/MSSA** | **SCC*mec* type^c^** | **ST^d^** | **# contigs** | **Largest contig** | **Total length** | **GC (%)** | **N50** | **N75** | **L50** | **L75** | **# N's per 100 kbp** | **num_seqs** | **Length** | **Depth** | **sum_len** | **min_len** | **avg_len** | **max_len** |
| --- | --- | --- | --- | --- | --- | --- | --- | --- | --- | --- | --- | --- | --- | --- | --- | --- | --- | --- | --- | --- | --- | --- | --- | --- | --- |
| **CV107** | SAMN14257665 | Cape Verde | HBS | 1997 | PA | nasal swab | MSSA | - | 669 | 22 | 799553 | 2764573 | 32.66 | 667084 | 182047 | 2 | 5 | 0.00 | 1575045 | 150 | 83 | 235247273 | 36 | 149.4 | 151 |
| **CV109** | SAMN14257664 | Cape Verde | HBS | 1997 | PA | nasal swab | MSSA | - | 121 | 50 | 433007 | 2787797 | 32.73 | 134592 | 70351 | 6 | 13 | 0.00 | 2027220 | 150 | 107 | 302683038 | 36 | 149.3 | 151 |
| **CV11** | SAMN14257663 | Cape Verde | HAN | 1997 | PA | nasal swab | MSSA | - | 30 | 48 | 278211 | 2771012 | 32.72 | 153681 | 109657 | 7 | 12 | 0.00 | 1040806 | 150 | 55 | 155406721 | 36 | 149.3 | 151 |
| **CV110** | SAMN14257662 | Cape Verde | HBS | 1997 | PA | nasal swab | MSSA | - | 669 | 30 | 700911 | 2827310 | 32.66 | 227342 | 182112 | 3 | 6 | 0.00 | 924358 | 150 | 49 | 138019572 | 36 | 149.3 | 151 |
| **CV115** | SAMN14257661 | Cape Verde | HBS | 1997 | HCW | nasal swab | MSSA | - | 30 | 40 | 423654 | 2753720 | 32.70 | 174931 | 85059 | 5 | 11 | 0.00 | 1426211 | 150 | 75 | 212779298 | 36 | 149.2 | 151 |
| **CV120** | SAMN14257660 | Cape Verde | HBS | 1997 | PA | nasal swab | MSSA | - | 30 | 37 | 395531 | 2779578 | 32.71 | 174839 | 133325 | 5 | 10 | 0.00 | 1845479 | 150 | 98 | 275463324 | 36 | 149.3 | 151 |
| **CV126** | SAMN14257659 | Cape Verde | HBS | 1997 | PA | nasal swab | MSSA | - | 8 | 33 | 961352 | 2823078 | 32.63 | 817405 | 135430 | 2 | 4 | 0.00 | 1977934 | 150 | 105 | 295419288 | 36 | 149.4 | 151 |
| **CV133** | SAMN14257658 | Cape Verde | HBS | 1997 | PA | nasal swab | MSSA | - | 121 | 55 | 433062 | 2835823 | 32.77 | 133900 | 60749 | 6 | 13 | 0.00 | 3614284 | 150 | 192 | 539403847 | 36 | 149.2 | 151 |
| **CV141** | SAMN14257657 | Cape Verde | HBS | 1997 | HCW | nasal swab | MSSA | - | 8 | 35 | 871028 | 2810227 | 32.66 | 845278 | 176141 | 2 | 4 | 0.00 | 3028622 | 150 | 161 | 451616492 | 36 | 149.1 | 151 |
| **CV144** | SAMN14257656 | Cape Verde | HBS | 1997 | PA | nasal swab | MSSA | - | 5980 | 38 | 799389 | 2886215 | 32.77 | 325459 | 172935 | 3 | 6 | 0.00 | 2671787 | 150 | 142 | 398852032 | 36 | 149.3 | 151 |
| **CV145** | SAMN14257655 | Cape Verde | HBS | 1997 | PA | nasal swab | MSSA | - | 669 | 26 | 895415 | 2867984 | 32.70 | 712639 | 193772 | 2 | 5 | 0.00 | 2201465 | 150 | 117 | 328559563 | 36 | 149.2 | 151 |
| **CV15** | SAMN14257654 | Cape Verde | HAN | 1997 | HCW | nasal swab | MSSA | - | 121 | 40 | 457453 | 2786059 | 32.71 | 165631 | 114588 | 6 | 10 | 0.00 | 2434317 | 150 | 129 | 363193788 | 36 | 149.2 | 151 |
| **CV151** | SAMN14257653 | Cape Verde | HBS | 1997 | PA | nasal swab | MSSA | - | 2300 | 29 | 829649 | 2736951 | 32.62 | 804761 | 192518 | 2 | 4 | 0.00 | 1892217 | 150 | 100 | 282298403 | 36 | 149.2 | 151 |
| **CV16** | SAMN14257652 | Cape Verde | HAN | 1997 | HCW | nasal swab | MSSA | - | 25 | 13 | 1462783 | 2849028 | 32.70 | 1462783 | 1045714 | 1 | 2 | 0.00 | 1750598 | 150 | 93 | 261431612 | 36 | 149.3 | 151 |
| **CV161** | SAMN14257651 | Cape Verde | HBS | 1997 | PA | nasal swab | MSSA | - | 669 | 29 | 712580 | 2827498 | 32.68 | 709145 | 182019 | 2 | 6 | 0.00 | 3430300 | 150 | 182 | 511880318 | 36 | 149.2 | 151 |
| **CV164** | SAMN14257650 | Cape Verde | HBS | 1997 | HCW | nasal swab | MSSA | - | 15 | 29 | 658732 | 2732394 | 32.69 | 251722 | 164141 | 4 | 7 | 0.00 | 1662473 | 150 | 88 | 248115056 | 36 | 149.2 | 151 |
| **CV168** | SAMN14257649 | Cape Verde | HBS | 1997 | HCW | nasal swab | MSSA | - | 25 | 18 | 1355066 | 2806867 | 32.64 | 999238 | 999238 | 2 | 2 | 0.00 | 3361129 | 150 | 178 | 501709801 | 36 | 149.3 | 151 |
| **CV169** | SAMN14257648 | Cape Verde | HBS | 1997 | HCW | nasal swab | MSSA | - | 45 | 67 | 923346 | 2891548 | 32.61 | 150750 | 83282 | 4 | 10 | 0.00 | 2709873 | 150 | 144 | 404383140 | 36 | 149.2 | 151 |
| **CV17** | SAMN14257647 | Cape Verde | HAN | 1997 | HCW | nasal swab | MSSA | - | 15 | 35 | 573290 | 2760893 | 32.74 | 254193 | 96834 | 4 | 7 | 0.00 | 2182234 | 150 | 116 | 325638937 | 36 | 149.2 | 151 |
| **CV173** | SAMN14257646 | Cape Verde | HBS | 1997 | HCW | nasal swab | MSSA | - | 669 | 37 | 852590 | 2840823 | 32.64 | 640998 | 121087 | 2 | 6 | 0.00 | 2633131 | 150 | 139 | 392958482 | 36 | 149.2 | 151 |
| **CV174** | SAMN14257645 | Cape Verde | HBS | 1997 | HCW | nasal swab | MSSA | - | 508 | 21 | 902274 | 2716189 | 32.78 | 656885 | 182400 | 2 | 4 | 0.00 | 4931440 | 150 | 262 | 736052991 | 36 | 149.3 | 151 |
| **CV178** | SAMN14257644 | Cape Verde | HAN | 2013 | PA | nasal swab | MSSA | - | 15 | 46 | 317363 | 2771386 | 32.72 | 253618 | 87373 | 5 | 11 | 0.00 | 2483829 | 150 | 132 | 370906177 | 36 | 149.3 | 151 |
| **CV179** | SAMN14257643 | Cape Verde | HAN | 2013 | PA | nasal swab | MSSA | - | 15 | 32 | 577225 | 2770612 | 32.74 | 436459 | 87087 | 3 | 8 | 0.00 | 1174291 | 150 | 62 | 175199132 | 36 | 149.2 | 151 |
| **CV18** | SAMN14257642 | Cape Verde | HAN | 1997 | HCW | nasal swab | MSSA | - | 45 | 70 | 876089 | 2822181 | 32.61 | 145850 | 98941 | 5 | 10 | 0.00 | 3736822 | 150 | 198 | 557164517 | 36 | 149.1 | 151 |
| **CV185A** | SAMN14257641 | Cape Verde | HAN | 2013 | PA | nasal swab | MSSA | - | 97 | 30 | 799132 | 2779661 | 32.73 | 697328 | 143127 | 2 | 5 | 0.00 | 2709628 | 150 | 144 | 404417494 | 36 | 149.3 | 151 |
| **CV188** | SAMN14257640 | Cape Verde | HAN | 2013 | PA | nasal swab | MRSA | IVa | 88 | 31 | 551889 | 2781701 | 32.70 | 424212 | 112804 | 3 | 6 | 0.00 | 3668829 | 150 | 195 | 547490597 | 36 | 149.2 | 151 |
| **CV196** | SAMN14257639 | Cape Verde | HAN | 2013 | HCW | nasal swab | MSSA | - | 152 | 46 | 514041 | 2763099 | 32.74 | 186247 | 103241 | 5 | 10 | 0.00 | 2020075 | 150 | 107 | 301503089 | 36 | 149.3 | 151 |
| **CV199** | SAMN14257638 | Cape Verde | HAN | 2013 | HCW | nasal swab | MSSA | - | 72 | 26 | 941935 | 2668923 | 32.71 | 401015 | 155050 | 2 | 5 | 0.00 | 1940076 | 150 | 103 | 289532211 | 36 | 149.2 | 151 |
| **CV204** | SAMN14257637 | Cape Verde | HAN | 2013 | HCW | nasal swab | MSSA | - | 188 | 32 | 553035 | 2728111 | 32.68 | 273690 | 118238 | 4 | 8 | 0.00 | 2209826 | 150 | 117 | 329799763 | 36 | 149.2 | 151 |
| **CV213** | SAMN14257636 | Cape Verde | HAN | 2013 | HCW | nasal swab | MSSA | - | 6 | 35 | 534800 | 2766314 | 32.72 | 223964 | 108441 | 4 | 9 | 0.00 | 1523517 | 150 | 80 | 227381741 | 36 | 149.2 | 151 |
| **CV214** | SAMN14257635 | Cape Verde | HAN | 2013 | HCW | nasal swab | MSSA | - | 5 | 36 | 956408 | 2800836 | 32.77 | 267232 | 125922 | 3 | 7 | 0.00 | 1201868 | 150 | 63 | 179402344 | 36 | 149.3 | 151 |
| **CV219** | SAMN14257634 | Cape Verde | HAN | 2013 | PA | nasal swab | MSSA | - | 152 | 47 | 514004 | 2712963 | 32.70 | 186374 | 97858 | 5 | 10 | 0.00 | 2509162 | 150 | 133 | 374194630 | 36 | 149.1 | 151 |
| **CV220A** | SAMN14257633 | Cape Verde | HAN | 2013 | PA | nasal swab | MSSA | - | 508 | 21 | 806914 | 2764351 | 32.80 | 806857 | 214933 | 2 | 4 | 0.00 | 2444004 | 150 | 129 | 364815724 | 36 | 149.3 | 151 |
| **CV225** | SAMN14257632 | Cape Verde | HAN | 2013 | PA | nasal swab | MSSA | - | 5981 | 28 | 763974 | 2769586 | 32.78 | 316473 | 138277 | 3 | 6 | 0.00 | 2359716 | 150 | 125 | 352146480 | 36 | 149.2 | 151 |
| **CV233** | SAMN14257631 | Cape Verde | HAN | 2013 | PA | nasal swab | MSSA | - | 121 | 43 | 433016 | 2762039 | 32.70 | 188098 | 109456 | 5 | 10 | 0.00 | 2351267 | 150 | 125 | 350918620 | 36 | 149.2 | 151 |
| **CV239** | SAMN14257630 | Cape Verde | HAN | 2013 | PA | nasal swab | MSSA | - | 1 | 15 | 1032554 | 2838001 | 32.72 | 621839 | 565265 | 2 | 3 | 0.00 | 3295333 | 150 | 175 | 492078264 | 36 | 149.3 | 151 |
| **CV241** | SAMN14257629 | Cape Verde | HAN | 2013 | HCW | nasal swab | MSSA | - | 152 | 46 | 542672 | 2760502 | 32.73 | 170824 | 95048 | 5 | 11 | 0.00 | 2501205 | 150 | 132 | 373116213 | 36 | 149.2 | 151 |
| **CV254** | SAMN14257628 | Cape Verde | HAN | 2013 | PA | nasal swab | MSSA | - | 152 | 44 | 513987 | 2740549 | 32.76 | 180947 | 101000 | 5 | 10 | 0.00 | 2220316 | 150 | 118 | 331484723 | 36 | 149.3 | 151 |
| **CV261** | SAMN14257627 | Cape Verde | HAN | 2013 | HCW | nasal swab | MSSA | - | 15 | 29 | 583924 | 2731456 | 32.70 | 253935 | 88251 | 4 | 8 | 0.00 | 2024174 | 150 | 107 | 302249546 | 36 | 149.3 | 151 |
| **CV262** | SAMN14257626 | Cape Verde | HAN | 2013 | HCW | nasal swab | MSSA | - | 72 | 26 | 669452 | 2724713 | 32.68 | 314861 | 182153 | 3 | 6 | 0.00 | 2220856 | 150 | 118 | 331317681 | 36 | 149.2 | 151 |
| **CV266A** | SAMN14257625 | Cape Verde | HAN | 2013 | HCW | nasal swab | MSSA | - | 22 | 58 | 408569 | 2774410 | 32.69 | 173967 | 73280 | 6 | 12 | 0.00 | 2266872 | 150 | 120 | 338325828 | 36 | 149.2 | 151 |
| **CV270** | SAMN14257624 | Cape Verde | HAN | 2013 | HCW | nasal swab | MSSA | - | 1 | 35 | 546645 | 2826780 | 32.69 | 264931 | 140287 | 4 | 7 | 0.00 | 2755034 | 150 | 146 | 411185264 | 36 | 149.2 | 151 |
| **CV274** | SAMN14257623 | Cape Verde | HAN | 2013 | HCW | nasal swab | MSSA | - | 72 | 26 | 711379 | 2748773 | 32.74 | 333116 | 181664 | 3 | 6 | 0.00 | 3480614 | 150 | 185 | 519582832 | 36 | 149.3 | 151 |
| **CV275** | SAMN14257622 | Cape Verde | HAN | 2013 | PA | nasal swab | MRSA | IVa | 8 | 51 | 825010 | 2860384 | 32.63 | 284081 | 104634 | 3 | 9 | 0.00 | 2392858 | 150 | 127 | 356932872 | 36 | 149.2 | 151 |
| **CV280** | SAMN14257621 | Cape Verde | HAN | 2013 | PA | nasal swab | MSSA | - | 152 | 41 | 513698 | 2713492 | 32.69 | 172853 | 106585 | 6 | 10 | 0.00 | 2263114 | 150 | 120 | 337913597 | 36 | 149.3 | 151 |
| **CV29** | SAMN14257620 | Cape Verde | HAN | 1997 | HCW | nasal swab | MSSA | - | 1 | 25 | 565726 | 2837582 | 32.71 | 546906 | 167445 | 3 | 6 | 0.00 | 2077680 | 150 | 110 | 310151654 | 36 | 149.3 | 151 |
| **CV290** | SAMN14257619 | Cape Verde | HAN | 2013 | PA | nasal swab | MSSA | - | 1 | 29 | 565549 | 2877426 | 32.74 | 348191 | 175751 | 3 | 7 | 0.00 | 1892210 | 150 | 100 | 282524647 | 36 | 149.3 | 151 |
| **CV307A** | SAMN14257618 | Cape Verde | HAN | 2013 | HCW | nasal swab | MSSA | - | 72 | 22 | 995716 | 2764471 | 32.71 | 403685 | 180642 | 2 | 5 | 0.00 | 3636335 | 150 | 193 | 542595908 | 36 | 149.2 | 151 |
| **CV314A** | SAMN14257617 | Cape Verde | HAN | 2013 | PA | nasal swab | MSSA | - | 508 | 20 | 731466 | 2723964 | 32.76 | 634175 | 301896 | 2 | 4 | 0.00 | 1766059 | 150 | 93 | 263607079 | 36 | 149.3 | 151 |
| **CV320** | SAMN14257616 | Cape Verde | HAN | 2013 | PA | nasal swab | MSSA | - | 152 | 38 | 514002 | 2754425 | 32.74 | 186246 | 103350 | 5 | 10 | 0.00 | 2571728 | 150 | 136 | 383594829 | 36 | 149.2 | 151 |
| **CV327A** | SAMN14257615 | Cape Verde | HAN | 2013 | PA | nasal swab | MSSA | - | 15 | 35 | 572261 | 2739335 | 32.69 | 255301 | 87980 | 4 | 8 | 0.00 | 1898129 | 150 | 100 | 283255071 | 36 | 149.2 | 151 |
| **CV330A** | SAMN14257614 | Cape Verde | HAN | 2013 | PA | nasal swab | MSSA | - | 152 | 44 | 346410 | 2721134 | 32.69 | 209202 | 103290 | 5 | 10 | 0.00 | 2736903 | 150 | 145 | 408702653 | 36 | 149.3 | 151 |
| **CV337** | SAMN14257613 | Cape Verde | HAN | 2013 | HCW | nasal swab | MRSA | VI | 5 | 43 | 500444 | 2792148 | 32.74 | 285758 | 189791 | 4 | 7 | 0.00 | 1679900 | 150 | 89 | 250652885 | 36 | 149.2 | 151 |
| **CV34** | SAMN14257612 | Cape Verde | HAN | 1997 | PA | nasal swab | MSSA | - | 1472 | 56 | 343010 | 2808670 | 32.73 | 172617 | 102123 | 6 | 12 | 0.00 | 1918338 | 150 | 101 | 286402390 | 36 | 149.3 | 151 |
| **CV346** | SAMN14257611 | Cape Verde | HAN | 2013 | PA | nasal swab | MRSA | IVa | 88 | 29 | 878071 | 2791315 | 32.70 | 362266 | 148219 | 3 | 5 | 0.00 | 1444828 | 150 | 76 | 215621866 | 36 | 149.2 | 151 |
| **CV348** | SAMN14257610 | Cape Verde | HAN | 2013 | HCW | nasal swab | MSSA | - | 25 | 23 | 1351358 | 2800153 | 32.64 | 766900 | 766900 | 2 | 2 | 0.00 | 2259000 | 150 | 120 | 337161253 | 36 | 149.3 | 151 |
| **CV356** | SAMN14257609 | Cape Verde | HBS | 2013 | HCW | nasal swab | MSSA | - | 508 | 24 | 1098959 | 2737726 | 32.73 | 902906 | 168768 | 2 | 3 | 0.00 | 1944023 | 150 | 103 | 290277705 | 36 | 149.3 | 151 |
| **CV361A** | SAMN14257608 | Cape Verde | HBS | 2013 | HCW | nasal swab | MSSA | - | 669 | 24 | 800192 | 2759692 | 32.69 | 666063 | 182105 | 2 | 6 | 0.00 | 2119013 | 150 | 112 | 316162218 | 36 | 149.2 | 151 |
| **CV371** | SAMN14257607 | Cape Verde | HBS | 2013 | PA | nasal swab | MSSA | - | 152 | 81 | 513818 | 2736119 | 32.69 | 185951 | 63785 | 5 | 11 | 0.00 | 2646512 | 150 | 140 | 394892829 | 36 | 149.2 | 151 |
| **CV390A** | SAMN14257606 | Cape Verde | HBS | 2013 | PA | nasal swab | MSSA | - | 97 | 29 | 433348 | 2736244 | 32.69 | 307448 | 186906 | 4 | 7 | 0.00 | 1883981 | 150 | 100 | 281102026 | 36 | 149.2 | 151 |
| **CV41** | SAMN14257605 | Cape Verde | HAN | 1997 | PA | nasal swab | MSSA | - | 72 | 26 | 539773 | 2726067 | 32.69 | 273496 | 180643 | 4 | 7 | 0.00 | 1270858 | 150 | 67 | 189712036 | 36 | 149.3 | 151 |
| **CV414** | SAMN14257604 | Cape Verde | HBS | 2013 | PA | nasal swab | MSSA | - | 188 | 55 | 752661 | 2779342 | 32.72 | 209571 | 117665 | 4 | 8 | 0.00 | 1890084 | 150 | 100 | 282155959 | 36 | 149.3 | 151 |
| **CV43** | SAMN14257603 | Cape Verde | HAN | 1997 | PA | nasal swab | MSSA | - | 1 | 26 | 633940 | 2873004 | 32.68 | 397164 | 166190 | 3 | 7 | 0.00 | 1990077 | 150 | 105 | 297169871 | 36 | 149.3 | 151 |
| **CV442** | SAMN14257602 | Cape Verde | HBS | 2013 | PA | nasal swab | MSSA | - | 1472 | 63 | 281616 | 2797226 | 32.70 | 170894 | 93054 | 7 | 12 | 0.00 | 1620094 | 150 | 86 | 241826963 | 36 | 149.3 | 151 |
| **CV443** | SAMN14257601 | Cape Verde | HBS | 2013 | PA | nasal swab | MSSA | - | 188 | 30 | 752968 | 2773188 | 32.74 | 252084 | 169966 | 4 | 7 | 0.00 | 1593658 | 150 | 84 | 237952692 | 36 | 149.3 | 151 |
| **CV462** | SAMN14257600 | Cape Verde | HBS | 2013 | HCW | nasal swab | MSSA | - | 1472 | 46 | 341845 | 2765667 | 32.71 | 170856 | 111691 | 6 | 11 | 0.00 | 2480235 | 150 | 131 | 370018847 | 36 | 149.2 | 151 |
| **CV464** | SAMN14257599 | Cape Verde | HBS | 2013 | PA | nasal swab | MSSA | - | 398 | 22 | 424173 | 2717680 | 32.83 | 329536 | 136928 | 4 | 7 | 0.00 | 1586550 | 150 | 84 | 236813767 | 36 | 149.3 | 151 |
| **CV476** | SAMN14257598 | Cape Verde | HBS | 2013 | PA | nasal swab | MSSA | - | 2498 | 46 | 543736 | 2795333 | 32.80 | 278432 | 126497 | 4 | 7 | 0.00 | 1718804 | 150 | 91 | 256445111 | 36 | 149.2 | 151 |
| **CV479** | SAMN14257597 | Cape Verde | HBS | 2013 | HCW | nasal swab | MSSA | - | 152 | 50 | 518305 | 2723988 | 32.68 | 186515 | 87195 | 5 | 11 | 0.00 | 2452921 | 150 | 130 | 366277350 | 36 | 149.3 | 151 |
| **CV485** | SAMN14257596 | Cape Verde | HBS | 2013 | HCW | nasal swab | MSSA | - | 5 | 33 | 969655 | 2733158 | 32.71 | 495510 | 125916 | 2 | 5 | 0.00 | 2617792 | 150 | 139 | 390794123 | 36 | 149.3 | 151 |
| **CV492** | SAMN14257595 | Cape Verde | HAN | 2014 | HCW | nasal swab | MRSA | VI | 5 | 39 | 623738 | 2792148 | 32.74 | 386594 | 189791 | 3 | 6 | 0.00 | 2532463 | 150 | 134 | 377783456 | 36 | 149.2 | 151 |
| **CV495** | SAMN14257594 | Cape Verde | HAN | 2014 | HCW | nasal swab | MRSA | VI | 5 | 41 | 500444 | 2791839 | 32.74 | 289927 | 189791 | 4 | 7 | 0.00 | 2143491 | 150 | 113 | 319949951 | 36 | 149.3 | 151 |
| **CV496** | SAMN14257593 | Cape Verde | HAN | 2014 | HCW | nasal swab | MSSA | - | 1472 | 49 | 341821 | 2767326 | 32.71 | 160379 | 112702 | 6 | 11 | 0.00 | 1964873 | 150 | 104 | 293192271 | 36 | 149.2 | 151 |
| **CV500A** | SAMN14257592 | Cape Verde | HAN | 2014 | PA | nasal swab | MSSA | - | 1 | 19 | 1050239 | 2806968 | 32.66 | 793914 | 541214 | 2 | 3 | 0.00 | 2488791 | 150 | 132 | 371467260 | 36 | 149.3 | 151 |
| **CV516A** | SAMN14257591 | Cape Verde | HAN | 2014 | PA | nasal swab | MSSA | - | 152 | 57 | 513128 | 2764599 | 32.73 | 160198 | 64232 | 5 | 12 | 0.00 | 3652200 | 150 | 194 | 544979580 | 36 | 149.2 | 151 |
| **CV531A** | SAMN14257590 | Cape Verde | HAN | 2014 | PA | nasal swab | MSSA | - | 4996 | 94 | 294400 | 2845389 | 32.66 | 150023 | 86631 | 7 | 14 | 0.00 | 2605923 | 150 | 138 | 389075343 | 36 | 149.3 | 151 |
| **CV532** | SAMN14257589 | Cape Verde | HAN | 2014 | PA | nasal swab | MSSA | - | 45 | 57 | 582270 | 2867799 | 32.63 | 141859 | 86683 | 6 | 12 | 0.00 | 2633194 | 150 | 139 | 392923872 | 36 | 149.2 | 151 |
| **CV533** | SAMN14257588 | Cape Verde | HAN | 2014 | PA | nasal swab | MSSA | - | 5 | 68 | 584493 | 2852566 | 32.77 | 230430 | 125916 | 4 | 8 | 0.00 | 2021206 | 150 | 107 | 301578916 | 36 | 149.2 | 151 |
| **CV534** | SAMN14257587 | Cape Verde | HAN | 2014 | PA | nasal swab | MSSA | - | 15 | 29 | 524780 | 2716419 | 32.73 | 254193 | 114508 | 4 | 7 | 0.00 | 1424711 | 150 | 75 | 212650502 | 36 | 149.3 | 151 |
| **CV540** | SAMN14257586 | Cape Verde | HAN | 2014 | PA | nasal swab | MSSA | - | 273 | 38 | 535475 | 2780631 | 32.75 | 307918 | 125917 | 4 | 7 | 0.00 | 1969874 | 150 | 104 | 294053405 | 36 | 149.3 | 151 |
| **CV548** | SAMN14257585 | Cape Verde | HAN | 2014 | HCW | nasal swab | MSSA | - | 152 | 44 | 349788 | 2719977 | 32.68 | 186374 | 106387 | 6 | 10 | 0.00 | 3931866 | 150 | 209 | 586749376 | 36 | 149.2 | 151 |
| **CV55** | SAMN14257584 | Cape Verde | HAN | 1997 | HCW | nasal swab | MSSA | - | 291 | 33 | 667929 | 2679485 | 32.79 | 249043 | 156057 | 4 | 7 | 0.00 | 2193145 | 150 | 116 | 327133699 | 36 | 149.2 | 151 |
| **CV558** | SAMN14257583 | Cape Verde | HAN | 2014 | PA | nasal swab | MSSA | - | 1472 | 47 | 349362 | 2766424 | 32.71 | 162321 | 105447 | 7 | 13 | 0.00 | 2005095 | 150 | 106 | 299286803 | 36 | 149.3 | 151 |
| **CV561** | SAMN14257582 | Cape Verde | HAN | 2014 | PA | nasal swab | MSSA | - | 15 | 28 | 576114 | 2769754 | 32.74 | 249815 | 121511 | 4 | 7 | 0.00 | 2307256 | 150 | 122 | 343267678 | 36 | 148.8 | 151 |
| **CV562A** | SAMN14257581 | Cape Verde | HAN | 2014 | PA | nasal swab | MSSA | - | 15 | 36 | 468212 | 2733133 | 32.70 | 255505 | 87822 | 4 | 9 | 0.00 | 2309318 | 150 | 122 | 343149813 | 36 | 148.6 | 151 |
| **CV573** | SAMN14257580 | Cape Verde | HAN | 2014 | HCW | nasal swab | MSSA | - | 508 | 28 | 802344 | 2806607 | 32.82 | 759207 | 194337 | 2 | 4 | 0.00 | 2161911 | 150 | 114 | 321388412 | 36 | 148.7 | 151 |
| **CV576** | SAMN14257579 | Cape Verde | HAN | 2014 | HCW | nasal swab | MSSA | - | 22 | 53 | 408459 | 2763375 | 32.70 | 127181 | 72713 | 7 | 14 | 0.00 | 1182047 | 150 | 62 | 176365910 | 36 | 149.2 | 151 |
| **CV58** | SAMN14257578 | Cape Verde | HAN | 1997 | HCW | nasal swab | MSSA | - | 2021 | 37 | 818962 | 2739521 | 32.66 | 559169 | 106147 | 2 | 6 | 0.00 | 1466947 | 150 | 77 | 219064638 | 36 | 149.3 | 151 |
| **CV581** | SAMN14257577 | Cape Verde | HAN | 2014 | HCW | nasal swab | MSSA | - | 72 | 32 | 679125 | 2756757 | 32.71 | 247108 | 148976 | 4 | 7 | 0.00 | 2570126 | 150 | 136 | 380566994 | 36 | 148.1 | 151 |
| **CV582** | SAMN14257576 | Cape Verde | HAN | 2014 | HCW | nasal swab | MSSA | - | 15 | 36 | 439572 | 2729214 | 32.72 | 251805 | 115392 | 5 | 9 | 0.00 | 2481124 | 150 | 131 | 367448974 | 36 | 148.1 | 151 |
| **CV587** | SAMN14257575 | Cape Verde | HAN | 2014 | PA | nasal swab | MSSA | - | 508 | 24 | 738644 | 2803764 | 32.82 | 434199 | 194468 | 3 | 5 | 0.00 | 2241711 | 150 | 119 | 334515748 | 36 | 149.2 | 151 |
| **CV591A** | SAMN14257574 | Cape Verde | HAN | 2014 | PA | nasal swab | MSSA | - | 45 | 52 | 580022 | 2832088 | 32.67 | 188758 | 86863 | 5 | 11 | 0.00 | 2526509 | 150 | 134 | 376720745 | 36 | 149.1 | 151 |
| **CV595** | SAMN14257573 | Cape Verde | HAN | 2014 | PA | nasal swab | MSSA | - | 22 | 59 | 405433 | 2811275 | 32.71 | 173968 | 92712 | 6 | 12 | 0.00 | 1890944 | 150 | 100 | 281059894 | 36 | 148.6 | 151 |
| **CV603** | SAMN14257572 | Cape Verde | HAN | 2014 | PA | nasal swab | MSSA | - | 97 | 34 | 798719 | 2749473 | 32.68 | 289959 | 167877 | 3 | 6 | 0.00 | 4827187 | 150 | 256 | 720715485 | 36 | 149.3 | 151 |
| **CV625A** | SAMN14257571 | Cape Verde | HAN | 2014 | HCW | nasal swab | MSSA | - | 273 | 35 | 640918 | 2779343 | 32.75 | 328447 | 187852 | 3 | 6 | 0.00 | 2229522 | 150 | 118 | 331014194 | 36 | 148.5 | 151 |
| **CV627** | SAMN14257570 | Cape Verde | HAN | 2014 | HCW | nasal swab | MSSA | - | 22 | 52 | 408513 | 2811764 | 32.71 | 152451 | 76732 | 7 | 13 | 0.00 | 2596685 | 150 | 138 | 387692291 | 36 | 149.3 | 151 |
| **CV634** | SAMN14257569 | Cape Verde | HAN | 2014 | HCW | nasal swab | MSSA | - | 152 | 55 | 513126 | 2753189 | 32.75 | 170328 | 67855 | 5 | 11 | 0.00 | 2020968 | 150 | 107 | 300123538 | 36 | 148.5 | 151 |
| **CV638** | SAMN14257568 | Cape Verde | HAN | 2014 | PA | nasal swab | MSSA | - | 508 | 28 | 759252 | 2764909 | 32.82 | 342231 | 137689 | 3 | 6 | 0.00 | 1431057 | 150 | 76 | 212513845 | 36 | 148.5 | 151 |
| **CV648** | SAMN14257567 | Cape Verde | HAN | 2014 | PA | nasal swab | MSSA | - | 22 | 51 | 405931 | 2759397 | 32.69 | 127183 | 65928 | 7 | 14 | 0.00 | 2277037 | 150 | 121 | 338423252 | 36 | 148.6 | 151 |
| **CV654** | SAMN14257566 | Cape Verde | HAN | 2014 | PA | nasal swab | MSSA | - | 5 | 40 | 898770 | 2781514 | 32.74 | 500201 | 125916 | 2 | 6 | 0.00 | 2198996 | 150 | 116 | 326834767 | 36 | 148.6 | 151 |
| **CV66** | SAMN14257565 | Cape Verde | HAN | 1997 | HCW | nasal swab | MSSA | - | 5 | 48 | 640882 | 2775673 | 32.74 | 325553 | 167907 | 3 | 6 | 0.00 | 2167584 | 150 | 115 | 323745671 | 36 | 149.4 | 151 |
| **CV669A** | SAMN14257564 | Cape Verde | HAN | 2014 | PA | nasal swab | MSSA | - | 15 | 37 | 403698 | 2722358 | 32.69 | 255185 | 87101 | 5 | 10 | 0.00 | 1878096 | 150 | 99 | 279156889 | 36 | 148.6 | 151 |
| **CV693** | SAMN14257563 | Cape Verde | HAN | 2014 | HCW | perianal swab | MRSA | VI | 5 | 41 | 499982 | 2790920 | 32.73 | 288480 | 189791 | 4 | 7 | 0.00 | 2127152 | 150 | 113 | 316163711 | 36 | 148.6 | 151 |
| **CV81** | SAMN14257562 | Cape Verde | HBS | 1997 | PA | nasal swab | MSSA | - | 508 | 20 | 1007378 | 2748228 | 32.81 | 902433 | 212695 | 2 | 3 | 0.00 | 2707164 | 150 | 143 | 403588961 | 36 | 149.1 | 151 |
| **CV85** | SAMN14257561 | Cape Verde | HBS | 1997 | PA | nasal swab | MSSA | - | 669 | 28 | 742947 | 2781045 | 32.64 | 667019 | 130125 | 2 | 5 | 0.00 | 2035383 | 150 | 108 | 303701360 | 36 | 149.2 | 151 |
| **CV87** | SAMN14257560 | Cape Verde | HBS | 1997 | PA | nasal swab | MSSA | - | 668 | 42 | 613935 | 2778960 | 32.78 | 169519 | 85108 | 5 | 11 | 0.00 | 1260150 | 150 | 66 | 187975341 | 36 | 149.2 | 151 |

^a^ HAN – Hospital Agostinho Neto; HBS – Hospital Baptista de Sousa

^b^ PA – patient; HCW – healthcare worker

^c^ SCC*mec* – staphylococcal chromosome cassette *mec*

^d^ ST – sequence type

Table S2: Virulence genes identified in *S. aureus* isolates from Cape Verde

| **VFs** | **Function** | Protease | Protease | Staphylokinase | Iron acquisition | Adhesin | Adhesin | Adhesin | Adhesin | Adhesin | Adhesin | Adhesin | Adhesin | Capsule | Capsule | Capsule | Capsule | Capsule | Capsule | Capsule | Capsule | Capsule | Capsule | Capsule | Capsule | Capsule | Capsule | Capsule | Capsule | Toxins | Toxins | Toxins | Toxins | Toxins | Toxins | Toxins | Toxins | Toxins | Toxins | immune evasion | immune evasion | immune evasion | immune evasion | immune evasion | immune evasion | immune evasion | immune evasion | immune evasion | immune evasion | immune evasion | immune evasion | immune evasion | immune evasion |
| --- | --- | --- | --- | --- | --- | --- | --- | --- | --- | --- | --- | --- | --- | --- | --- | --- | --- | --- | --- | --- | --- | --- | --- | --- | --- | --- | --- | --- | --- | --- | --- | --- | --- | --- | --- | --- | --- | --- | --- | --- | --- | --- | --- | --- | --- | --- | --- | --- | --- | --- | --- | --- | --- |
|  |  |  |  |  |  |  |  |  | Intercellular adhesin | Intercellular adhesin | Intercellular adhesin | Intercellular adhesin | Intercellular adhesin |  |  |  |  |  |  |  |  |  |  |  |  |  |  |  |  |  |  |  |  |  |  |  |  |  |  | Hemolysin | Hemolysin | Hemolysin | Hemolysin | Leukocidin | Leukocidin | Leukocidin | Complement inhibitor |  |  |  |  |  |  |
| **Isolate** | **ST** | ***aur*** | ***ssp*** | ***sak*** | ***isdD*** | ***cna*** | ***ebp*** | ***spa*** | ***icaA*** | ***icaB*** | ***icaC*** | ***icaD*** | ***icaR*** | ***cap8A*** | ***cap8B*** | ***cap8C*** | ***cap8D*** | ***cap8E*** | ***cap8F*** | ***cap8G*** | ***cap8H*** | ***cap8I*** | ***cap8J*** | ***cap8K*** | ***cap8L*** | ***cap8M*** | ***cap8N*** | ***cap8O*** | ***cap8P*** | ***eta*** | ***sea*** | ***seb*** | ***sec*** | ***sed*** | ***seh*** | ***selk*** | ***sell*** | ***selq*** | ***tsst-1*** | ***hla/hly*** | ***hlb*** | ***hld*** | ***hlg*** | ***lukDE*** | ***lukFS*** | ***lukM*** | ***scn*** | ***chp*** | ***esxA*** | ***esaA*** | ***esaB*** | ***essC*** | ***esxB*** |
| CV107 | 669 | 1 | 1 | 1 | 1 | 0 | 1 | 1 | 1 | 1 | 1 | 1 | 1 | 1 | 1 | 1 | 1 | 1 | 1 | 1 | 0 | 0 | 0 | 0 | 1 | 1 | 1 | 1 | 1 | 0 | 0 | 0 | 1 | 1 | 0 | 0 | 1 | 0 | 0 | 1 | 0 | 1 | 1 | 1 | 0 | 0 | 1 | 1 | 1 | 1 | 1 | 1 | 1 |
| CV109 | 121 | 1 | 1 | 1 | 1 | 0 | 0 | 0 | 1 | 1 | 1 | 1 | 1 | 1 | 1 | 1 | 1 | 1 | 1 | 1 | 1 | 1 | 1 | 1 | 1 | 1 | 1 | 1 | 1 | 0 | 0 | 1 | 0 | 0 | 0 | 0 | 0 | 0 | 0 | 1 | 0 | 1 | 1 | 1 | 1 | 0 | 1 | 0 | 1 | 1 | 1 | 1 | 1 |
| CV11 | 30 | 1 | 1 | 1 | 1 | 0 | 1 | 1 | 1 | 1 | 1 | 1 | 1 | 1 | 1 | 1 | 1 | 1 | 1 | 1 | 1 | 1 | 1 | 1 | 1 | 1 | 1 | 1 | 1 | 0 | 0 | 0 | 0 | 0 | 0 | 0 | 0 | 0 | 0 | 1 | 0 | 1 | 1 | 0 | 1 | 0 | 1 | 1 | 1 | 1 | 1 | 0 | 0 |
| CV110 | 669 | 1 | 1 | 1 | 1 | 0 | 1 | 1 | 1 | 1 | 1 | 1 | 1 | 1 | 1 | 1 | 1 | 1 | 1 | 1 | 0 | 0 | 0 | 0 | 1 | 1 | 1 | 1 | 1 | 0 | 0 | 0 | 1 | 1 | 0 | 1 | 1 | 1 | 0 | 1 | 0 | 1 | 1 | 1 | 0 | 0 | 1 | 1 | 1 | 1 | 1 | 1 | 1 |
| CV115 | 30 | 1 | 1 | 1 | 1 | 0 | 1 | 0 | 1 | 1 | 1 | 1 | 1 | 1 | 1 | 1 | 1 | 1 | 1 | 1 | 1 | 1 | 1 | 1 | 1 | 1 | 1 | 1 | 1 | 0 | 1 | 0 | 0 | 0 | 0 | 0 | 0 | 0 | 0 | 1 | 0 | 1 | 1 | 0 | 0 | 0 | 1 | 1 | 1 | 1 | 1 | 0 | 0 |
| CV120 | 30 | 1 | 1 | 1 | 1 | 0 | 1 | 0 | 1 | 1 | 1 | 1 | 1 | 1 | 1 | 1 | 1 | 1 | 1 | 1 | 1 | 1 | 1 | 1 | 1 | 1 | 1 | 1 | 1 | 0 | 0 | 0 | 0 | 0 | 0 | 0 | 0 | 0 | 0 | 1 | 0 | 1 | 1 | 0 | 1 | 0 | 1 | 1 | 1 | 1 | 1 | 0 | 0 |
| CV126 | 8 | 1 | 1 | 1 | 1 | 0 | 1 | 1 | 1 | 1 | 1 | 1 | 1 | 1 | 1 | 1 | 1 | 1 | 1 | 1 | 0 | 0 | 0 | 0 | 1 | 1 | 1 | 1 | 1 | 0 | 1 | 0 | 0 | 0 | 0 | 0 | 0 | 0 | 0 | 1 | 0 | 1 | 1 | 1 | 0 | 0 | 1 | 0 | 1 | 1 | 1 | 1 | 1 |
| CV133 | 121 | 1 | 1 | 1 | 1 | 0 | 0 | 0 | 1 | 1 | 1 | 1 | 1 | 1 | 1 | 1 | 1 | 1 | 1 | 1 | 1 | 1 | 1 | 1 | 1 | 1 | 1 | 1 | 1 | 0 | 0 | 1 | 0 | 0 | 0 | 0 | 0 | 0 | 0 | 1 | 0 | 1 | 1 | 1 | 1 | 0 | 1 | 0 | 1 | 1 | 1 | 1 | 1 |
| CV141 | 8 | 1 | 1 | 1 | 1 | 0 | 1 | 1 | 1 | 1 | 1 | 1 | 1 | 1 | 1 | 1 | 1 | 1 | 1 | 1 | 0 | 0 | 0 | 0 | 1 | 1 | 1 | 1 | 1 | 0 | 1 | 0 | 0 | 0 | 0 | 0 | 0 | 0 | 0 | 1 | 0 | 1 | 1 | 1 | 0 | 0 | 1 | 0 | 1 | 1 | 1 | 1 | 1 |
| CV144 | 5980 | 1 | 1 | 1 | 1 | 0 | 1 | 0 | 1 | 1 | 1 | 1 | 1 | 1 | 1 | 1 | 1 | 1 | 1 | 1 | 0 | 0 | 0 | 0 | 1 | 1 | 1 | 1 | 1 | 0 | 0 | 0 | 1 | 0 | 0 | 0 | 1 | 0 | 1 | 1 | 0 | 1 | 1 | 1 | 0 | 0 | 1 | 1 | 1 | 1 | 1 | 1 | 1 |
| CV145 | 669 | 1 | 1 | 1 | 1 | 0 | 1 | 1 | 1 | 1 | 1 | 1 | 1 | 1 | 1 | 1 | 1 | 1 | 1 | 1 | 0 | 0 | 0 | 0 | 1 | 1 | 1 | 1 | 1 | 0 | 0 | 0 | 1 | 1 | 0 | 1 | 1 | 1 | 0 | 1 | 0 | 1 | 1 | 1 | 0 | 0 | 1 | 1 | 1 | 1 | 1 | 1 | 1 |
| CV15 | 121 | 1 | 1 | 1 | 1 | 0 | 1 | 0 | 1 | 1 | 1 | 1 | 1 | 1 | 1 | 1 | 1 | 1 | 1 | 1 | 1 | 1 | 1 | 1 | 1 | 1 | 1 | 1 | 1 | 0 | 0 | 0 | 0 | 0 | 0 | 0 | 0 | 0 | 0 | 1 | 0 | 1 | 1 | 1 | 1 | 0 | 1 | 0 | 1 | 1 | 1 | 1 | 1 |
| CV151 | 2300 | 1 | 1 | 1 | 1 | 0 | 1 | 1 | 0 | 0 | 0 | 0 | 0 | 1 | 1 | 1 | 1 | 1 | 1 | 1 | 0 | 0 | 0 | 0 | 1 | 1 | 1 | 1 | 1 | 0 | 1 | 0 | 0 | 0 | 0 | 0 | 0 | 0 | 0 | 1 | 0 | 1 | 1 | 1 | 0 | 0 | 1 | 0 | 1 | 1 | 1 | 1 | 1 |
| CV16 | 25 | 1 | 1 | 1 | 1 | 0 | 1 | 1 | 1 | 1 | 1 | 1 | 1 | 1 | 1 | 1 | 1 | 1 | 1 | 1 | 0 | 0 | 0 | 0 | 1 | 1 | 1 | 1 | 1 | 0 | 0 | 1 | 0 | 0 | 0 | 0 | 0 | 0 | 0 | 1 | 0 | 1 | 1 | 1 | 1 | 0 | 1 | 1 | 1 | 1 | 1 | 1 | 1 |
| CV161 | 669 | 1 | 1 | 1 | 1 | 0 | 1 | 1 | 1 | 1 | 1 | 1 | 1 | 1 | 1 | 1 | 1 | 1 | 1 | 1 | 0 | 0 | 0 | 0 | 1 | 1 | 1 | 1 | 1 | 0 | 0 | 0 | 1 | 1 | 0 | 1 | 1 | 1 | 0 | 1 | 0 | 1 | 1 | 1 | 0 | 0 | 1 | 1 | 1 | 1 | 1 | 1 | 1 |
| CV164 | 15 | 1 | 1 | 0 | 1 | 0 | 1 | 1 | 1 | 1 | 1 | 1 | 1 | 1 | 1 | 1 | 1 | 1 | 1 | 1 | 1 | 1 | 1 | 1 | 1 | 1 | 1 | 1 | 1 | 0 | 0 | 0 | 0 | 0 | 0 | 0 | 0 | 0 | 0 | 1 | 0 | 1 | 1 | 1 | 0 | 0 | 1 | 1 | 1 | 1 | 1 | 0 | 0 |
| CV168 | 25 | 1 | 1 | 1 | 1 | 0 | 1 | 0 | 1 | 1 | 1 | 1 | 1 | 1 | 1 | 1 | 1 | 1 | 1 | 1 | 0 | 0 | 0 | 0 | 1 | 1 | 1 | 1 | 1 | 0 | 0 | 1 | 0 | 0 | 0 | 0 | 0 | 0 | 0 | 1 | 0 | 1 | 1 | 1 | 1 | 0 | 1 | 1 | 1 | 1 | 1 | 1 | 1 |
| CV169 | 45 | 1 | 1 | 1 | 1 | 0 | 1 | 0 | 1 | 1 | 1 | 1 | 1 | 1 | 1 | 1 | 1 | 1 | 1 | 1 | 0 | 0 | 0 | 0 | 1 | 1 | 1 | 1 | 1 | 0 | 0 | 0 | 0 | 0 | 0 | 0 | 0 | 0 | 0 | 1 | 0 | 1 | 1 | 0 | 0 | 0 | 1 | 1 | 1 | 1 | 1 | 1 | 1 |
| CV17 | 15 | 1 | 1 | 0 | 1 | 0 | 1 | 0 | 1 | 1 | 1 | 1 | 1 | 1 | 1 | 1 | 1 | 1 | 1 | 1 | 1 | 1 | 1 | 1 | 1 | 1 | 1 | 1 | 1 | 1 | 0 | 0 | 0 | 0 | 0 | 0 | 0 | 0 | 0 | 1 | 0 | 1 | 1 | 1 | 0 | 0 | 1 | 1 | 1 | 1 | 1 | 0 | 0 |
| CV173 | 669 | 1 | 1 | 1 | 1 | 0 | 1 | 1 | 1 | 1 | 1 | 1 | 1 | 1 | 1 | 1 | 1 | 1 | 1 | 1 | 0 | 0 | 0 | 0 | 1 | 1 | 1 | 1 | 1 | 0 | 0 | 0 | 1 | 1 | 0 | 1 | 1 | 1 | 0 | 1 | 0 | 1 | 1 | 1 | 0 | 0 | 1 | 1 | 1 | 1 | 1 | 1 | 1 |
| CV174 | 508 | 1 | 1 | 1 | 1 | 0 | 1 | 1 | 1 | 1 | 1 | 1 | 1 | 1 | 1 | 1 | 1 | 1 | 1 | 1 | 1 | 1 | 1 | 1 | 1 | 1 | 1 | 1 | 1 | 0 | 0 | 0 | 0 | 0 | 0 | 0 | 0 | 0 | 0 | 1 | 0 | 1 | 1 | 0 | 0 | 0 | 1 | 1 | 1 | 1 | 1 | 1 | 1 |
| CV178 | 15 | 1 | 1 | 0 | 1 | 0 | 1 | 0 | 1 | 1 | 1 | 1 | 1 | 1 | 1 | 1 | 1 | 1 | 1 | 1 | 1 | 1 | 1 | 1 | 1 | 1 | 1 | 1 | 1 | 0 | 0 | 0 | 0 | 0 | 0 | 0 | 0 | 0 | 0 | 1 | 0 | 1 | 1 | 1 | 0 | 0 | 1 | 1 | 1 | 1 | 1 | 0 | 0 |
| CV179 | 15 | 1 | 1 | 0 | 1 | 0 | 1 | 0 | 1 | 1 | 1 | 1 | 1 | 1 | 1 | 1 | 1 | 1 | 1 | 1 | 1 | 1 | 1 | 1 | 1 | 1 | 1 | 1 | 1 | 0 | 0 | 0 | 0 | 0 | 0 | 0 | 0 | 0 | 0 | 1 | 0 | 1 | 1 | 1 | 0 | 0 | 1 | 1 | 1 | 1 | 1 | 0 | 0 |
| CV18 | 45 | 1 | 1 | 1 | 1 | 0 | 1 | 0 | 1 | 1 | 1 | 1 | 1 | 1 | 1 | 1 | 1 | 1 | 1 | 1 | 0 | 0 | 0 | 0 | 1 | 1 | 1 | 1 | 1 | 0 | 0 | 0 | 0 | 0 | 0 | 0 | 0 | 0 | 0 | 1 | 0 | 1 | 1 | 0 | 0 | 0 | 1 | 1 | 1 | 1 | 1 | 1 | 1 |
| CV185A | 97 | 1 | 1 | 1 | 1 | 0 | 1 | 1 | 1 | 1 | 1 | 1 | 1 | 1 | 1 | 1 | 1 | 1 | 1 | 1 | 0 | 0 | 0 | 0 | 1 | 1 | 1 | 1 | 1 | 0 | 0 | 0 | 0 | 0 | 0 | 0 | 0 | 0 | 0 | 1 | 0 | 1 | 1 | 1 | 0 | 0 | 1 | 0 | 1 | 1 | 1 | 1 | 1 |
| CV188 | 88 | 1 | 1 | 1 | 1 | 0 | 1 | 1 | 1 | 1 | 1 | 1 | 1 | 1 | 1 | 1 | 1 | 1 | 1 | 1 | 1 | 1 | 1 | 1 | 1 | 1 | 1 | 1 | 1 | 1 | 0 | 0 | 0 | 0 | 0 | 0 | 0 | 0 | 0 | 1 | 0 | 1 | 1 | 1 | 0 | 0 | 1 | 1 | 1 | 1 | 1 | 1 | 1 |
| CV196 | 152 | 1 | 1 | 1 | 1 | 0 | 1 | 0 | 1 | 1 | 1 | 1 | 1 | 1 | 1 | 1 | 1 | 1 | 1 | 1 | 0 | 0 | 0 | 0 | 1 | 1 | 1 | 1 | 1 | 0 | 0 | 0 | 0 | 0 | 0 | 0 | 0 | 0 | 0 | 1 | 1 | 1 | 1 | 0 | 1 | 0 | 1 | 0 | 1 | 1 | 1 | 1 | 1 |
| CV199 | 72 | 1 | 1 | 0 | 1 | 0 | 1 | 1 | 1 | 1 | 1 | 1 | 1 | 1 | 1 | 1 | 1 | 1 | 1 | 1 | 0 | 0 | 0 | 0 | 1 | 1 | 1 | 1 | 1 | 0 | 0 | 0 | 1 | 0 | 0 | 0 | 1 | 0 | 0 | 1 | 1 | 1 | 1 | 1 | 0 | 0 | 0 | 0 | 1 | 1 | 1 | 1 | 1 |
| CV204 | 188 | 1 | 1 | 1 | 1 | 0 | 1 | 0 | 1 | 1 | 1 | 1 | 1 | 1 | 1 | 1 | 1 | 1 | 1 | 1 | 1 | 1 | 1 | 1 | 1 | 1 | 1 | 1 | 1 | 0 | 0 | 0 | 0 | 0 | 0 | 0 | 0 | 0 | 0 | 1 | 0 | 1 | 1 | 1 | 0 | 0 | 1 | 1 | 1 | 1 | 1 | 0 | 0 |
| CV213 | 6 | 1 | 1 | 1 | 1 | 0 | 1 | 1 | 1 | 1 | 1 | 1 | 1 | 1 | 1 | 1 | 1 | 1 | 1 | 1 | 1 | 1 | 1 | 1 | 1 | 1 | 1 | 1 | 1 | 0 | 1 | 0 | 0 | 0 | 0 | 0 | 0 | 0 | 0 | 1 | 0 | 1 | 1 | 1 | 0 | 0 | 1 | 0 | 1 | 1 | 1 | 1 | 1 |
| CV214 | 5 | 1 | 1 | 1 | 1 | 0 | 1 | 0 | 1 | 1 | 1 | 1 | 1 | 1 | 1 | 1 | 1 | 1 | 1 | 1 | 0 | 0 | 0 | 0 | 1 | 1 | 1 | 1 | 1 | 0 | 1 | 0 | 0 | 0 | 0 | 0 | 0 | 0 | 0 | 1 | 0 | 1 | 1 | 1 | 0 | 0 | 1 | 0 | 1 | 1 | 1 | 1 | 1 |
| CV219 | 152 | 1 | 1 | 1 | 1 | 0 | 1 | 0 | 1 | 1 | 1 | 1 | 1 | 1 | 1 | 1 | 1 | 1 | 1 | 1 | 0 | 0 | 0 | 0 | 1 | 1 | 1 | 1 | 1 | 0 | 0 | 0 | 0 | 0 | 0 | 0 | 0 | 0 | 0 | 1 | 1 | 1 | 1 | 0 | 1 | 0 | 1 | 0 | 1 | 1 | 1 | 1 | 1 |
| CV220A | 508 | 1 | 1 | 1 | 1 | 0 | 1 | 1 | 1 | 1 | 1 | 1 | 1 | 1 | 1 | 1 | 1 | 1 | 1 | 1 | 1 | 1 | 1 | 1 | 1 | 1 | 1 | 1 | 1 | 0 | 0 | 0 | 1 | 0 | 0 | 0 | 1 | 0 | 0 | 1 | 0 | 1 | 1 | 0 | 0 | 0 | 1 | 1 | 1 | 1 | 1 | 1 | 1 |
| CV225 | 5981 | 1 | 1 | 1 | 1 | 0 | 1 | 1 | 1 | 1 | 1 | 1 | 1 | 1 | 1 | 1 | 1 | 1 | 1 | 1 | 1 | 1 | 1 | 1 | 1 | 1 | 1 | 1 | 1 | 0 | 0 | 0 | 1 | 0 | 0 | 0 | 1 | 0 | 0 | 1 | 0 | 1 | 1 | 0 | 0 | 0 | 1 | 1 | 1 | 1 | 1 | 1 | 1 |
| CV233 | 121 | 1 | 1 | 1 | 1 | 0 | 0 | 0 | 1 | 1 | 1 | 1 | 1 | 1 | 1 | 1 | 1 | 1 | 1 | 1 | 1 | 1 | 1 | 1 | 1 | 1 | 1 | 1 | 1 | 0 | 0 | 1 | 0 | 0 | 0 | 0 | 0 | 0 | 0 | 1 | 0 | 1 | 1 | 1 | 1 | 0 | 1 | 0 | 1 | 1 | 1 | 1 | 1 |
| CV239 | 1 | 1 | 1 | 1 | 1 | 0 | 1 | 1 | 1 | 1 | 1 | 1 | 1 | 1 | 1 | 1 | 1 | 1 | 1 | 1 | 1 | 1 | 1 | 1 | 1 | 1 | 1 | 1 | 1 | 0 | 1 | 0 | 0 | 0 | 1 | 1 | 0 | 1 | 0 | 1 | 0 | 1 | 1 | 1 | 1 | 0 | 1 | 0 | 1 | 1 | 1 | 1 | 1 |
| CV241 | 152 | 1 | 1 | 1 | 1 | 0 | 1 | 0 | 1 | 1 | 1 | 1 | 1 | 1 | 1 | 1 | 1 | 1 | 1 | 1 | 0 | 0 | 0 | 0 | 1 | 1 | 1 | 1 | 1 | 0 | 0 | 0 | 0 | 0 | 0 | 0 | 0 | 0 | 0 | 1 | 1 | 1 | 1 | 0 | 0 | 0 | 1 | 0 | 1 | 1 | 1 | 1 | 1 |
| CV254 | 152 | 1 | 1 | 1 | 1 | 0 | 1 | 0 | 1 | 1 | 1 | 1 | 1 | 1 | 1 | 1 | 1 | 1 | 1 | 1 | 0 | 0 | 0 | 0 | 1 | 1 | 1 | 1 | 1 | 0 | 0 | 0 | 0 | 0 | 0 | 0 | 0 | 0 | 0 | 1 | 1 | 1 | 1 | 0 | 1 | 0 | 1 | 0 | 1 | 1 | 1 | 1 | 1 |
| CV261 | 15 | 1 | 1 | 0 | 1 | 0 | 1 | 0 | 1 | 1 | 1 | 1 | 1 | 1 | 1 | 1 | 1 | 1 | 1 | 1 | 1 | 1 | 1 | 1 | 1 | 1 | 1 | 1 | 1 | 0 | 1 | 0 | 0 | 0 | 0 | 0 | 0 | 0 | 0 | 1 | 0 | 1 | 1 | 1 | 1 | 0 | 1 | 1 | 1 | 1 | 1 | 0 | 0 |
| CV262 | 72 | 1 | 1 | 1 | 1 | 0 | 1 | 1 | 1 | 1 | 1 | 1 | 1 | 1 | 1 | 1 | 1 | 1 | 1 | 1 | 0 | 0 | 0 | 0 | 1 | 1 | 1 | 1 | 1 | 0 | 0 | 0 | 1 | 0 | 0 | 0 | 1 | 0 | 0 | 1 | 0 | 1 | 1 | 1 | 0 | 0 | 1 | 1 | 1 | 1 | 1 | 1 | 1 |
| CV266A | 22 | 1 | 1 | 1 | 1 | 0 | 1 | 1 | 1 | 1 | 1 | 1 | 1 | 1 | 1 | 1 | 1 | 1 | 1 | 1 | 0 | 0 | 0 | 0 | 1 | 1 | 1 | 1 | 1 | 0 | 0 | 0 | 0 | 0 | 0 | 0 | 0 | 0 | 1 | 1 | 0 | 1 | 1 | 0 | 0 | 0 | 1 | 1 | 1 | 1 | 1 | 0 | 0 |
| CV270 | 1 | 1 | 1 | 1 | 1 | 1 | 1 | 0 | 1 | 1 | 1 | 1 | 1 | 1 | 1 | 1 | 1 | 1 | 1 | 1 | 1 | 1 | 1 | 1 | 1 | 1 | 1 | 1 | 1 | 0 | 1 | 1 | 0 | 0 | 1 | 0 | 0 | 0 | 0 | 1 | 0 | 1 | 1 | 1 | 0 | 0 | 1 | 0 | 1 | 1 | 1 | 1 | 1 |
| CV274 | 72 | 1 | 1 | 1 | 1 | 0 | 1 | 1 | 1 | 1 | 1 | 1 | 1 | 1 | 1 | 1 | 1 | 1 | 1 | 1 | 0 | 0 | 0 | 0 | 1 | 1 | 1 | 1 | 1 | 0 | 0 | 0 | 1 | 0 | 0 | 0 | 1 | 0 | 0 | 1 | 0 | 1 | 1 | 1 | 0 | 0 | 1 | 1 | 1 | 1 | 1 | 1 | 1 |
| CV275 | 8 | 1 | 1 | 1 | 1 | 0 | 1 | 1 | 1 | 1 | 1 | 1 | 1 | 1 | 1 | 1 | 1 | 1 | 1 | 1 | 0 | 0 | 0 | 0 | 1 | 1 | 1 | 1 | 1 | 0 | 0 | 0 | 0 | 0 | 0 | 0 | 0 | 0 | 0 | 1 | 0 | 1 | 1 | 1 | 1 | 0 | 1 | 1 | 1 | 1 | 1 | 1 | 1 |
| CV280 | 152 | 1 | 1 | 1 | 1 | 0 | 1 | 0 | 1 | 1 | 1 | 1 | 1 | 1 | 1 | 1 | 1 | 1 | 1 | 1 | 0 | 0 | 0 | 0 | 1 | 1 | 1 | 1 | 1 | 0 | 0 | 0 | 0 | 0 | 0 | 0 | 0 | 0 | 0 | 1 | 1 | 1 | 1 | 0 | 1 | 0 | 1 | 0 | 1 | 1 | 1 | 1 | 1 |
| CV29 | 1 | 1 | 1 | 1 | 1 | 0 | 1 | 1 | 1 | 1 | 1 | 1 | 1 | 1 | 1 | 1 | 1 | 1 | 1 | 1 | 1 | 1 | 1 | 1 | 1 | 1 | 1 | 1 | 1 | 0 | 1 | 0 | 0 | 0 | 1 | 1 | 0 | 1 | 0 | 1 | 0 | 1 | 1 | 1 | 1 | 0 | 1 | 0 | 1 | 1 | 1 | 1 | 1 |
| CV290 | 1 | 1 | 1 | 1 | 1 | 1 | 1 | 1 | 1 | 1 | 1 | 1 | 1 | 1 | 1 | 1 | 1 | 1 | 1 | 1 | 1 | 1 | 1 | 1 | 1 | 1 | 1 | 1 | 1 | 0 | 1 | 0 | 1 | 0 | 1 | 1 | 1 | 1 | 1 | 1 | 0 | 1 | 1 | 1 | 0 | 0 | 1 | 0 | 1 | 1 | 1 | 1 | 1 |
| CV307A | 72 | 1 | 1 | 1 | 1 | 0 | 1 | 1 | 1 | 1 | 1 | 1 | 1 | 1 | 1 | 1 | 1 | 1 | 1 | 1 | 0 | 0 | 0 | 0 | 1 | 1 | 1 | 1 | 1 | 0 | 0 | 0 | 1 | 0 | 0 | 0 | 1 | 0 | 0 | 1 | 0 | 1 | 1 | 1 | 0 | 0 | 1 | 1 | 1 | 1 | 1 | 1 | 1 |
| CV314A | 508 | 1 | 1 | 1 | 1 | 0 | 1 | 1 | 1 | 1 | 1 | 1 | 1 | 1 | 1 | 1 | 1 | 1 | 1 | 1 | 1 | 1 | 1 | 1 | 1 | 1 | 1 | 1 | 1 | 0 | 0 | 0 | 1 | 0 | 0 | 0 | 1 | 0 | 1 | 1 | 0 | 1 | 1 | 0 | 0 | 0 | 1 | 1 | 1 | 1 | 1 | 1 | 1 |
| CV320 | 152 | 1 | 1 | 1 | 1 | 0 | 1 | 0 | 1 | 1 | 1 | 1 | 1 | 1 | 1 | 1 | 1 | 1 | 1 | 1 | 0 | 0 | 0 | 0 | 1 | 1 | 1 | 1 | 1 | 0 | 0 | 0 | 0 | 0 | 0 | 0 | 0 | 0 | 0 | 1 | 1 | 1 | 1 | 0 | 1 | 0 | 1 | 0 | 1 | 1 | 1 | 1 | 1 |
| CV327A | 15 | 1 | 1 | 0 | 1 | 0 | 1 | 0 | 1 | 1 | 1 | 1 | 1 | 1 | 1 | 1 | 1 | 1 | 1 | 1 | 1 | 1 | 1 | 1 | 1 | 1 | 1 | 1 | 1 | 0 | 0 | 0 | 0 | 0 | 0 | 0 | 0 | 0 | 0 | 1 | 0 | 1 | 1 | 1 | 0 | 0 | 1 | 1 | 1 | 1 | 1 | 0 | 0 |
| CV330A | 152 | 1 | 1 | 1 | 1 | 0 | 1 | 0 | 1 | 1 | 1 | 1 | 1 | 1 | 1 | 1 | 1 | 1 | 1 | 1 | 0 | 0 | 0 | 0 | 1 | 1 | 1 | 1 | 1 | 0 | 0 | 0 | 0 | 0 | 0 | 0 | 0 | 0 | 0 | 1 | 1 | 1 | 1 | 0 | 1 | 0 | 1 | 0 | 1 | 1 | 1 | 1 | 1 |
| CV337 | 5 | 1 | 1 | 1 | 1 | 0 | 1 | 0 | 1 | 1 | 1 | 1 | 1 | 1 | 1 | 1 | 1 | 1 | 1 | 1 | 0 | 0 | 0 | 0 | 1 | 1 | 1 | 1 | 1 | 0 | 1 | 0 | 0 | 0 | 0 | 0 | 0 | 0 | 0 | 1 | 0 | 1 | 1 | 1 | 0 | 0 | 1 | 0 | 1 | 1 | 1 | 1 | 1 |
| CV34 | 1472 | 1 | 1 | 1 | 1 | 0 | 1 | 1 | 1 | 1 | 1 | 1 | 1 | 1 | 1 | 1 | 1 | 1 | 1 | 1 | 1 | 1 | 1 | 1 | 1 | 1 | 1 | 1 | 1 | 0 | 0 | 0 | 0 | 0 | 0 | 0 | 0 | 0 | 0 | 1 | 0 | 1 | 1 | 0 | 1 | 0 | 1 | 1 | 1 | 1 | 1 | 0 | 0 |
| CV346 | 88 | 1 | 1 | 1 | 1 | 0 | 1 | 1 | 1 | 1 | 1 | 1 | 1 | 1 | 1 | 1 | 1 | 1 | 1 | 1 | 1 | 1 | 1 | 1 | 1 | 1 | 1 | 1 | 1 | 1 | 0 | 0 | 0 | 0 | 0 | 0 | 0 | 0 | 0 | 1 | 0 | 1 | 1 | 1 | 0 | 0 | 1 | 1 | 1 | 1 | 1 | 1 | 1 |
| CV348 | 25 | 1 | 1 | 1 | 1 | 0 | 1 | 1 | 1 | 1 | 1 | 1 | 1 | 1 | 1 | 1 | 1 | 1 | 1 | 1 | 0 | 0 | 0 | 0 | 1 | 1 | 1 | 1 | 1 | 0 | 0 | 1 | 0 | 0 | 0 | 0 | 0 | 0 | 0 | 1 | 0 | 1 | 1 | 1 | 0 | 0 | 1 | 1 | 1 | 1 | 1 | 1 | 1 |
| CV356 | 508 | 1 | 1 | 1 | 1 | 0 | 1 | 1 | 1 | 1 | 1 | 1 | 1 | 1 | 1 | 1 | 1 | 1 | 1 | 1 | 1 | 1 | 1 | 1 | 1 | 1 | 1 | 1 | 1 | 0 | 0 | 0 | 1 | 0 | 0 | 0 | 1 | 0 | 1 | 1 | 0 | 1 | 1 | 0 | 0 | 0 | 1 | 1 | 1 | 1 | 1 | 1 | 1 |
| CV361A | 669 | 1 | 1 | 1 | 1 | 0 | 1 | 1 | 1 | 1 | 1 | 1 | 1 | 1 | 1 | 1 | 1 | 1 | 1 | 1 | 0 | 0 | 0 | 0 | 1 | 1 | 1 | 1 | 1 | 0 | 0 | 0 | 1 | 0 | 0 | 1 | 1 | 1 | 0 | 1 | 0 | 1 | 1 | 1 | 0 | 0 | 1 | 1 | 1 | 1 | 1 | 1 | 1 |
| CV371 | 152 | 1 | 1 | 1 | 1 | 0 | 1 | 0 | 1 | 1 | 1 | 1 | 1 | 1 | 1 | 1 | 1 | 1 | 1 | 1 | 0 | 0 | 0 | 0 | 1 | 1 | 1 | 1 | 1 | 0 | 0 | 0 | 0 | 0 | 0 | 0 | 0 | 0 | 0 | 1 | 0 | 1 | 1 | 0 | 1 | 0 | 1 | 1 | 1 | 1 | 1 | 1 | 1 |
| CV390A | 97 | 1 | 1 | 1 | 1 | 0 | 1 | 0 | 1 | 1 | 1 | 1 | 1 | 1 | 1 | 1 | 1 | 1 | 1 | 1 | 0 | 0 | 0 | 0 | 1 | 1 | 1 | 1 | 1 | 0 | 0 | 0 | 0 | 0 | 0 | 0 | 0 | 0 | 0 | 1 | 0 | 1 | 1 | 1 | 0 | 0 | 1 | 0 | 1 | 1 | 1 | 1 | 1 |
| CV41 | 72 | 1 | 1 | 1 | 1 | 0 | 1 | 1 | 1 | 1 | 1 | 1 | 1 | 1 | 1 | 1 | 1 | 1 | 1 | 1 | 0 | 0 | 0 | 0 | 1 | 1 | 1 | 1 | 1 | 0 | 0 | 0 | 1 | 0 | 0 | 0 | 1 | 0 | 0 | 1 | 0 | 1 | 1 | 1 | 0 | 0 | 1 | 1 | 1 | 1 | 1 | 1 | 1 |
| CV414 | 188 | 1 | 1 | 1 | 1 | 0 | 1 | 0 | 1 | 1 | 1 | 1 | 1 | 1 | 1 | 1 | 1 | 1 | 1 | 1 | 1 | 1 | 1 | 1 | 1 | 1 | 1 | 1 | 1 | 0 | 0 | 0 | 0 | 0 | 0 | 0 | 0 | 0 | 0 | 1 | 0 | 1 | 1 | 1 | 0 | 0 | 1 | 1 | 1 | 1 | 1 | 0 | 0 |
| CV43 | 1 | 1 | 1 | 1 | 1 | 1 | 1 | 1 | 1 | 1 | 1 | 1 | 1 | 1 | 1 | 1 | 1 | 1 | 1 | 1 | 1 | 1 | 1 | 1 | 1 | 1 | 1 | 1 | 1 | 0 | 1 | 0 | 1 | 0 | 1 | 1 | 1 | 1 | 1 | 1 | 0 | 1 | 1 | 1 | 1 | 0 | 1 | 0 | 1 | 1 | 1 | 1 | 1 |
| CV442 | 1472 | 1 | 1 | 1 | 1 | 0 | 1 | 0 | 1 | 1 | 1 | 1 | 1 | 1 | 1 | 1 | 1 | 1 | 1 | 1 | 1 | 1 | 1 | 1 | 1 | 1 | 1 | 1 | 1 | 0 | 0 | 0 | 0 | 0 | 0 | 0 | 0 | 0 | 0 | 1 | 0 | 1 | 1 | 0 | 1 | 0 | 1 | 1 | 1 | 1 | 1 | 0 | 0 |
| CV443 | 188 | 1 | 1 | 1 | 1 | 0 | 1 | 0 | 1 | 1 | 1 | 1 | 1 | 1 | 1 | 1 | 1 | 1 | 1 | 1 | 1 | 1 | 1 | 1 | 1 | 1 | 1 | 1 | 1 | 0 | 0 | 0 | 0 | 0 | 0 | 0 | 0 | 0 | 0 | 1 | 0 | 1 | 1 | 1 | 0 | 0 | 1 | 0 | 1 | 1 | 1 | 0 | 0 |
| CV462 | 1472 | 1 | 1 | 1 | 1 | 0 | 1 | 1 | 1 | 1 | 1 | 1 | 1 | 1 | 1 | 1 | 1 | 1 | 1 | 1 | 1 | 1 | 1 | 1 | 1 | 1 | 1 | 1 | 1 | 0 | 0 | 0 | 0 | 0 | 0 | 0 | 0 | 0 | 0 | 1 | 0 | 1 | 1 | 0 | 0 | 0 | 1 | 1 | 1 | 1 | 1 | 0 | 0 |
| CV464 | 398 | 1 | 1 | 0 | 1 | 0 | 1 | 0 | 1 | 1 | 1 | 1 | 1 | 1 | 1 | 1 | 1 | 1 | 1 | 1 | 0 | 0 | 0 | 0 | 1 | 1 | 1 | 1 | 1 | 0 | 0 | 0 | 0 | 0 | 0 | 0 | 0 | 0 | 0 | 1 | 0 | 1 | 1 | 0 | 0 | 0 | 1 | 1 | 1 | 1 | 1 | 0 | 0 |
| CV476 | 2498 | 1 | 1 | 1 | 1 | 0 | 1 | 1 | 1 | 1 | 1 | 1 | 1 | 1 | 1 | 1 | 1 | 1 | 1 | 1 | 1 | 1 | 1 | 1 | 1 | 1 | 1 | 1 | 1 | 0 | 0 | 0 | 1 | 0 | 0 | 0 | 1 | 0 | 1 | 1 | 0 | 1 | 1 | 0 | 0 | 0 | 1 | 1 | 1 | 1 | 1 | 1 | 1 |
| CV479 | 152 | 1 | 1 | 1 | 1 | 0 | 1 | 0 | 1 | 1 | 1 | 1 | 1 | 1 | 1 | 1 | 1 | 1 | 1 | 1 | 0 | 0 | 0 | 0 | 1 | 1 | 1 | 1 | 1 | 0 | 0 | 0 | 0 | 0 | 0 | 0 | 0 | 0 | 0 | 1 | 1 | 1 | 1 | 0 | 1 | 0 | 1 | 0 | 1 | 1 | 1 | 1 | 1 |
| CV485 | 5 | 1 | 1 | 0 | 1 | 0 | 1 | 1 | 1 | 1 | 1 | 1 | 1 | 1 | 1 | 1 | 1 | 1 | 1 | 1 | 0 | 0 | 0 | 0 | 1 | 1 | 1 | 1 | 1 | 0 | 0 | 0 | 0 | 0 | 0 | 0 | 0 | 0 | 0 | 1 | 1 | 1 | 1 | 1 | 0 | 0 | 0 | 0 | 1 | 1 | 1 | 1 | 1 |
| CV492 | 5 | 1 | 1 | 1 | 1 | 0 | 1 | 0 | 1 | 1 | 1 | 1 | 1 | 1 | 1 | 1 | 1 | 1 | 1 | 1 | 0 | 0 | 0 | 0 | 1 | 1 | 1 | 1 | 1 | 0 | 1 | 0 | 0 | 0 | 0 | 0 | 0 | 0 | 0 | 1 | 0 | 1 | 1 | 1 | 0 | 0 | 1 | 0 | 1 | 1 | 1 | 1 | 1 |
| CV495 | 5 | 1 | 1 | 1 | 1 | 0 | 1 | 0 | 1 | 1 | 1 | 1 | 1 | 1 | 1 | 1 | 1 | 1 | 1 | 1 | 0 | 0 | 0 | 0 | 1 | 1 | 1 | 1 | 1 | 0 | 1 | 0 | 0 | 0 | 0 | 0 | 0 | 0 | 0 | 1 | 0 | 1 | 1 | 1 | 0 | 0 | 1 | 0 | 1 | 1 | 1 | 1 | 1 |
| CV496 | 1472 | 1 | 1 | 1 | 1 | 0 | 1 | 1 | 1 | 1 | 1 | 1 | 1 | 1 | 1 | 1 | 1 | 1 | 1 | 1 | 1 | 1 | 1 | 1 | 1 | 1 | 1 | 1 | 1 | 0 | 0 | 0 | 0 | 0 | 0 | 0 | 0 | 0 | 0 | 1 | 0 | 1 | 1 | 0 | 0 | 0 | 1 | 1 | 1 | 1 | 1 | 0 | 0 |
| CV500A | 1 | 1 | 1 | 1 | 1 | 1 | 1 | 1 | 1 | 1 | 1 | 1 | 1 | 1 | 1 | 1 | 0 | 0 | 0 | 0 | 0 | 1 | 1 | 1 | 1 | 1 | 1 | 1 | 1 | 0 | 1 | 1 | 0 | 0 | 1 | 1 | 0 | 1 | 0 | 1 | 0 | 1 | 1 | 1 | 1 | 0 | 1 | 0 | 1 | 1 | 1 | 1 | 1 |
| CV516A | 152 | 1 | 1 | 1 | 1 | 0 | 1 | 0 | 1 | 1 | 1 | 1 | 1 | 1 | 1 | 1 | 1 | 1 | 1 | 1 | 0 | 0 | 0 | 0 | 1 | 1 | 1 | 1 | 1 | 0 | 0 | 0 | 0 | 0 | 0 | 0 | 0 | 0 | 0 | 1 | 1 | 1 | 1 | 0 | 1 | 0 | 1 | 0 | 1 | 1 | 1 | 1 | 1 |
| CV531A | 4996 | 1 | 1 | 1 | 1 | 0 | 1 | 0 | 1 | 1 | 1 | 1 | 1 | 1 | 1 | 1 | 1 | 1 | 1 | 1 | 0 | 0 | 0 | 0 | 1 | 1 | 1 | 1 | 1 | 0 | 0 | 0 | 0 | 0 | 0 | 0 | 0 | 0 | 0 | 1 | 0 | 1 | 1 | 0 | 1 | 0 | 1 | 1 | 1 | 1 | 1 | 1 | 1 |
| CV532 | 45 | 1 | 1 | 1 | 1 | 0 | 1 | 0 | 1 | 1 | 1 | 1 | 1 | 1 | 1 | 1 | 1 | 1 | 1 | 1 | 0 | 0 | 0 | 0 | 1 | 1 | 1 | 1 | 1 | 0 | 0 | 0 | 0 | 0 | 0 | 0 | 0 | 0 | 0 | 1 | 0 | 1 | 1 | 0 | 0 | 0 | 1 | 1 | 1 | 1 | 1 | 1 | 1 |
| CV533 | 5 | 1 | 1 | 1 | 1 | 0 | 1 | 0 | 1 | 1 | 1 | 1 | 1 | 1 | 1 | 1 | 1 | 1 | 1 | 1 | 0 | 0 | 0 | 0 | 1 | 1 | 1 | 1 | 1 | 0 | 1 | 0 | 0 | 0 | 0 | 0 | 0 | 0 | 0 | 1 | 0 | 1 | 1 | 1 | 1 | 0 | 1 | 0 | 1 | 1 | 1 | 1 | 1 |
| CV534 | 15 | 1 | 1 | 0 | 1 | 0 | 1 | 0 | 1 | 1 | 1 | 1 | 1 | 1 | 1 | 1 | 1 | 1 | 1 | 1 | 1 | 1 | 1 | 1 | 1 | 1 | 1 | 1 | 1 | 1 | 0 | 0 | 0 | 0 | 0 | 0 | 0 | 0 | 0 | 1 | 0 | 1 | 1 | 1 | 0 | 0 | 1 | 1 | 1 | 1 | 1 | 0 | 0 |
| CV540 | 273 | 1 | 1 | 1 | 1 | 0 | 1 | 1 | 1 | 1 | 1 | 1 | 1 | 1 | 1 | 1 | 1 | 1 | 1 | 1 | 0 | 0 | 0 | 0 | 1 | 1 | 1 | 1 | 1 | 0 | 1 | 0 | 0 | 0 | 0 | 0 | 0 | 0 | 0 | 1 | 0 | 1 | 1 | 1 | 0 | 0 | 1 | 0 | 1 | 1 | 1 | 1 | 1 |
| CV548 | 152 | 1 | 1 | 1 | 1 | 0 | 1 | 0 | 1 | 1 | 1 | 1 | 1 | 1 | 1 | 1 | 1 | 1 | 1 | 1 | 0 | 0 | 0 | 0 | 1 | 1 | 1 | 1 | 1 | 0 | 0 | 0 | 0 | 0 | 0 | 0 | 0 | 0 | 0 | 1 | 1 | 1 | 1 | 0 | 1 | 0 | 1 | 0 | 1 | 1 | 1 | 1 | 1 |
| CV55 | 291 | 1 | 1 | 0 | 1 | 0 | 1 | 1 | 1 | 1 | 1 | 1 | 1 | 1 | 1 | 1 | 1 | 1 | 1 | 1 | 0 | 0 | 0 | 0 | 1 | 1 | 1 | 1 | 1 | 0 | 0 | 0 | 0 | 0 | 0 | 0 | 0 | 0 | 0 | 1 | 1 | 1 | 1 | 1 | 0 | 0 | 0 | 0 | 1 | 1 | 1 | 1 | 1 |
| CV558 | 1472 | 1 | 1 | 1 | 1 | 0 | 1 | 1 | 1 | 1 | 1 | 1 | 1 | 1 | 1 | 1 | 1 | 1 | 1 | 1 | 1 | 1 | 1 | 1 | 1 | 1 | 1 | 1 | 1 | 0 | 0 | 0 | 0 | 0 | 0 | 0 | 0 | 0 | 0 | 1 | 0 | 1 | 1 | 0 | 0 | 0 | 1 | 1 | 1 | 1 | 1 | 0 | 0 |
| CV561 | 15 | 1 | 1 | 0 | 1 | 0 | 1 | 0 | 1 | 1 | 1 | 1 | 1 | 1 | 1 | 1 | 1 | 1 | 1 | 1 | 1 | 1 | 1 | 1 | 1 | 1 | 1 | 1 | 1 | 0 | 0 | 0 | 0 | 0 | 0 | 0 | 0 | 0 | 0 | 1 | 0 | 1 | 1 | 1 | 0 | 0 | 1 | 1 | 1 | 1 | 1 | 0 | 0 |
| CV562A | 15 | 1 | 1 | 0 | 1 | 0 | 1 | 0 | 1 | 1 | 1 | 1 | 1 | 1 | 1 | 1 | 1 | 1 | 1 | 1 | 1 | 1 | 1 | 1 | 1 | 1 | 1 | 1 | 1 | 0 | 1 | 0 | 0 | 0 | 0 | 0 | 0 | 0 | 0 | 1 | 0 | 1 | 1 | 1 | 1 | 0 | 1 | 1 | 1 | 1 | 1 | 0 | 0 |
| CV573 | 508 | 1 | 1 | 1 | 1 | 0 | 1 | 1 | 1 | 1 | 1 | 1 | 1 | 1 | 1 | 1 | 1 | 1 | 1 | 1 | 1 | 1 | 1 | 1 | 1 | 1 | 1 | 1 | 1 | 0 | 0 | 0 | 1 | 0 | 0 | 0 | 1 | 0 | 0 | 1 | 0 | 1 | 1 | 0 | 0 | 0 | 1 | 1 | 1 | 1 | 1 | 1 | 1 |
| CV576 | 22 | 1 | 1 | 1 | 1 | 0 | 1 | 0 | 1 | 1 | 1 | 1 | 1 | 1 | 1 | 1 | 1 | 1 | 1 | 1 | 0 | 0 | 0 | 0 | 1 | 1 | 1 | 1 | 1 | 0 | 0 | 0 | 0 | 0 | 0 | 0 | 0 | 0 | 1 | 1 | 0 | 1 | 1 | 0 | 0 | 0 | 1 | 1 | 1 | 1 | 1 | 0 | 0 |
| CV58 | 2021 | 1 | 1 | 1 | 1 | 0 | 1 | 1 | 1 | 1 | 1 | 1 | 1 | 1 | 1 | 1 | 1 | 1 | 1 | 1 | 0 | 0 | 0 | 0 | 1 | 1 | 1 | 1 | 1 | 0 | 1 | 0 | 0 | 0 | 0 | 0 | 0 | 0 | 0 | 1 | 0 | 1 | 1 | 1 | 0 | 0 | 1 | 0 | 1 | 1 | 1 | 1 | 1 |
| CV581 | 72 | 1 | 1 | 1 | 1 | 0 | 1 | 1 | 1 | 1 | 1 | 1 | 1 | 1 | 1 | 1 | 1 | 1 | 1 | 1 | 0 | 0 | 0 | 0 | 1 | 1 | 1 | 1 | 1 | 0 | 0 | 0 | 1 | 0 | 0 | 0 | 1 | 0 | 0 | 1 | 0 | 1 | 1 | 1 | 0 | 0 | 1 | 1 | 1 | 1 | 1 | 1 | 1 |
| CV582 | 15 | 1 | 1 | 0 | 1 | 0 | 1 | 1 | 1 | 1 | 1 | 1 | 1 | 1 | 1 | 1 | 1 | 1 | 1 | 1 | 1 | 1 | 1 | 1 | 1 | 1 | 1 | 1 | 1 | 0 | 0 | 0 | 0 | 0 | 0 | 0 | 0 | 0 | 0 | 1 | 0 | 1 | 1 | 1 | 0 | 0 | 1 | 1 | 1 | 1 | 1 | 0 | 0 |
| CV587 | 508 | 1 | 1 | 1 | 1 | 0 | 1 | 1 | 1 | 1 | 1 | 1 | 1 | 1 | 1 | 1 | 1 | 1 | 1 | 1 | 1 | 1 | 1 | 1 | 1 | 1 | 1 | 1 | 1 | 0 | 0 | 0 | 1 | 0 | 0 | 0 | 1 | 0 | 0 | 1 | 0 | 1 | 1 | 0 | 0 | 0 | 1 | 1 | 1 | 1 | 1 | 1 | 1 |
| CV591A | 45 | 1 | 1 | 1 | 1 | 0 | 1 | 0 | 1 | 1 | 1 | 1 | 1 | 1 | 1 | 1 | 1 | 1 | 1 | 1 | 0 | 0 | 0 | 0 | 1 | 1 | 1 | 1 | 1 | 0 | 0 | 0 | 0 | 0 | 0 | 0 | 0 | 0 | 0 | 1 | 0 | 1 | 1 | 0 | 0 | 0 | 1 | 1 | 1 | 1 | 1 | 1 | 1 |
| CV595 | 22 | 1 | 1 | 1 | 1 | 0 | 1 | 1 | 1 | 1 | 1 | 1 | 1 | 1 | 1 | 1 | 1 | 1 | 1 | 1 | 0 | 0 | 0 | 0 | 1 | 1 | 1 | 1 | 1 | 0 | 0 | 0 | 0 | 0 | 0 | 0 | 0 | 0 | 1 | 1 | 0 | 1 | 1 | 0 | 0 | 0 | 1 | 1 | 1 | 1 | 1 | 0 | 0 |
| CV603 | 97 | 1 | 1 | 1 | 1 | 0 | 1 | 0 | 1 | 1 | 1 | 1 | 1 | 1 | 1 | 1 | 1 | 1 | 1 | 1 | 0 | 0 | 0 | 0 | 1 | 1 | 1 | 1 | 1 | 0 | 0 | 0 | 0 | 0 | 0 | 0 | 0 | 0 | 0 | 1 | 0 | 1 | 1 | 1 | 0 | 0 | 1 | 0 | 1 | 1 | 1 | 1 | 1 |
| CV625A | 273 | 1 | 1 | 1 | 1 | 0 | 1 | 1 | 1 | 1 | 1 | 1 | 1 | 1 | 1 | 1 | 1 | 1 | 1 | 1 | 0 | 0 | 0 | 0 | 1 | 1 | 1 | 1 | 1 | 0 | 1 | 0 | 0 | 0 | 0 | 0 | 0 | 0 | 0 | 1 | 0 | 1 | 1 | 1 | 0 | 0 | 1 | 0 | 1 | 1 | 1 | 1 | 1 |
| CV627 | 22 | 1 | 1 | 1 | 1 | 0 | 1 | 1 | 1 | 1 | 1 | 1 | 1 | 1 | 1 | 1 | 1 | 1 | 1 | 1 | 0 | 0 | 0 | 0 | 1 | 1 | 1 | 1 | 1 | 0 | 0 | 0 | 0 | 0 | 0 | 0 | 0 | 0 | 1 | 1 | 0 | 1 | 1 | 0 | 0 | 0 | 1 | 1 | 1 | 1 | 1 | 0 | 0 |
| CV634 | 152 | 1 | 1 | 1 | 1 | 0 | 1 | 0 | 1 | 1 | 1 | 1 | 1 | 1 | 1 | 1 | 1 | 1 | 1 | 1 | 0 | 0 | 0 | 0 | 1 | 1 | 1 | 1 | 1 | 0 | 0 | 0 | 0 | 0 | 0 | 0 | 0 | 0 | 0 | 1 | 1 | 1 | 1 | 0 | 1 | 0 | 1 | 0 | 1 | 1 | 1 | 1 | 1 |
| CV638 | 508 | 1 | 1 | 0 | 1 | 0 | 1 | 1 | 1 | 1 | 1 | 1 | 1 | 1 | 1 | 1 | 1 | 1 | 1 | 1 | 1 | 1 | 1 | 1 | 1 | 1 | 1 | 1 | 1 | 0 | 0 | 0 | 1 | 0 | 0 | 0 | 1 | 0 | 0 | 1 | 1 | 1 | 1 | 0 | 0 | 0 | 0 | 0 | 1 | 1 | 1 | 1 | 1 |
| CV648 | 22 | 1 | 1 | 1 | 1 | 0 | 1 | 1 | 1 | 1 | 1 | 1 | 1 | 1 | 1 | 1 | 1 | 1 | 1 | 1 | 0 | 0 | 0 | 0 | 1 | 1 | 1 | 1 | 1 | 0 | 0 | 0 | 0 | 0 | 0 | 0 | 0 | 0 | 1 | 1 | 0 | 1 | 1 | 0 | 0 | 0 | 1 | 1 | 1 | 1 | 1 | 0 | 0 |
| CV654 | 5 | 1 | 1 | 1 | 1 | 0 | 1 | 0 | 1 | 1 | 1 | 1 | 1 | 1 | 1 | 1 | 1 | 1 | 1 | 1 | 0 | 0 | 0 | 0 | 1 | 1 | 1 | 1 | 1 | 0 | 1 | 0 | 0 | 0 | 0 | 0 | 0 | 0 | 0 | 1 | 1 | 1 | 1 | 1 | 0 | 0 | 0 | 0 | 1 | 1 | 1 | 1 | 1 |
| CV66 | 5 | 1 | 1 | 1 | 1 | 0 | 1 | 1 | 1 | 1 | 1 | 1 | 1 | 1 | 1 | 1 | 1 | 1 | 1 | 1 | 0 | 0 | 0 | 0 | 1 | 1 | 1 | 1 | 1 | 0 | 1 | 0 | 0 | 0 | 0 | 0 | 0 | 0 | 0 | 1 | 0 | 1 | 1 | 1 | 0 | 0 | 1 | 0 | 1 | 1 | 1 | 1 | 1 |
| CV669A | 15 | 1 | 1 | 0 | 1 | 0 | 1 | 0 | 1 | 1 | 1 | 1 | 1 | 1 | 1 | 1 | 1 | 1 | 1 | 1 | 1 | 1 | 1 | 1 | 1 | 1 | 1 | 1 | 1 | 0 | 0 | 0 | 0 | 0 | 0 | 0 | 0 | 0 | 0 | 1 | 0 | 1 | 1 | 1 | 0 | 0 | 1 | 1 | 1 | 1 | 1 | 0 | 0 |
| CV693 | 5 | 1 | 1 | 1 | 1 | 0 | 1 | 0 | 1 | 1 | 1 | 1 | 1 | 1 | 1 | 1 | 1 | 1 | 1 | 1 | 0 | 0 | 0 | 0 | 1 | 1 | 1 | 1 | 1 | 0 | 1 | 0 | 0 | 0 | 0 | 0 | 0 | 0 | 0 | 1 | 0 | 1 | 1 | 1 | 0 | 0 | 1 | 0 | 1 | 1 | 1 | 1 | 1 |
| CV81 | 508 | 1 | 1 | 1 | 1 | 0 | 1 | 1 | 1 | 1 | 1 | 1 | 1 | 1 | 1 | 1 | 1 | 1 | 1 | 1 | 1 | 1 | 1 | 1 | 1 | 1 | 1 | 1 | 1 | 0 | 0 | 0 | 0 | 0 | 0 | 0 | 0 | 0 | 0 | 1 | 0 | 1 | 1 | 0 | 0 | 0 | 1 | 1 | 1 | 1 | 1 | 1 | 1 |
| CV85 | 669 | 1 | 1 | 1 | 1 | 0 | 1 | 1 | 1 | 1 | 1 | 1 | 1 | 1 | 1 | 1 | 1 | 1 | 1 | 1 | 0 | 0 | 0 | 0 | 1 | 1 | 1 | 1 | 1 | 0 | 0 | 0 | 1 | 1 | 0 | 1 | 1 | 1 | 0 | 1 | 0 | 1 | 1 | 1 | 0 | 0 | 1 | 1 | 1 | 1 | 1 | 1 | 1 |
| CV87 | 668 | 1 | 1 | 1 | 1 | 0 | 1 | 1 | 1 | 1 | 1 | 1 | 1 | 1 | 1 | 1 | 1 | 1 | 1 | 1 | 1 | 1 | 1 | 1 | 1 | 1 | 1 | 1 | 1 | 0 | 0 | 0 | 0 | 0 | 0 | 0 | 0 | 0 | 0 | 1 | 0 | 1 | 1 | 1 | 0 | 0 | 1 | 1 | 1 | 1 | 1 | 0 | 0 |

0 – absence; 1 - presence

Table S3: Antibiotic resistance genes identified in *S. aureus* isolates from Cape Verde

|  |  | **Antibiotic resistance genes** | | | | | | | | | | | | | | | | | | |
| --- | --- | --- | --- | --- | --- | --- | --- | --- | --- | --- | --- | --- | --- | --- | --- | --- | --- | --- | --- | --- |
| Isolate | **ST** | ***tetM*** | ***tetL*** | ***tetK*** | ***msrA*** | ***mecA*** | ***mecR1*** | ***fusC*** | ***ermT*** | ***ermC*** | ***mphC*** | ***dfrC*** | ***dfrG*** | ***blaZ*** | ***aph(3')-III*** | ***cfrC*** | ***vgaA*** | ***mepA*** | ***mep1*** | ***sat4*** |
| CV107 | 669 | 0 | 0 | 0 | 0 | 0 | 0 | 0 | 0 | 0 | 0 | 0 | 0 | 1 | 0 | 0 | 0 | 1 | 1 | 0 |
| CV109 | 121 | 0 | 0 | 1 | 0 | 0 | 0 | 0 | 0 | 0 | 0 | 0 | 0 | 1 | 0 | 0 | 0 | 1 | 1 | 0 |
| CV11 | 30 | 0 | 0 | 1 | 0 | 0 | 0 | 0 | 0 | 1 | 0 | 0 | 0 | 1 | 0 | 0 | 0 | 1 | 1 | 0 |
| CV110 | 669 | 0 | 0 | 0 | 0 | 0 | 0 | 0 | 0 | 0 | 0 | 0 | 0 | 1 | 0 | 0 | 0 | 1 | 1 | 0 |
| CV115 | 30 | 0 | 0 | 0 | 0 | 0 | 0 | 0 | 0 | 0 | 0 | 0 | 0 | 1 | 0 | 0 | 0 | 1 | 1 | 0 |
| CV120 | 30 | 0 | 0 | 1 | 0 | 0 | 0 | 0 | 0 | 0 | 0 | 0 | 0 | 1 | 0 | 0 | 0 | 1 | 1 | 0 |
| CV126 | 8 | 0 | 0 | 0 | 0 | 0 | 0 | 0 | 0 | 0 | 0 | 0 | 0 | 1 | 0 | 0 | 0 | 1 | 1 | 0 |
| CV133 | 121 | 0 | 0 | 1 | 0 | 0 | 0 | 0 | 0 | 0 | 0 | 0 | 0 | 1 | 0 | 0 | 0 | 1 | 1 | 0 |
| CV141 | 8 | 0 | 0 | 1 | 0 | 0 | 0 | 0 | 0 | 0 | 0 | 0 | 0 | 1 | 0 | 0 | 0 | 1 | 1 | 0 |
| CV144 | 5980 | 1 | 1 | 0 | 0 | 0 | 0 | 0 | 0 | 0 | 0 | 0 | 0 | 1 | 0 | 0 | 0 | 1 | 1 | 0 |
| CV145 | 669 | 0 | 0 | 0 | 0 | 0 | 0 | 0 | 0 | 0 | 0 | 0 | 0 | 1 | 0 | 0 | 0 | 1 | 1 | 0 |
| CV15 | 121 | 0 | 0 | 1 | 0 | 0 | 0 | 0 | 0 | 0 | 0 | 0 | 0 | 1 | 0 | 0 | 0 | 1 | 1 | 0 |
| CV151 | 2300 | 0 | 0 | 0 | 0 | 0 | 0 | 0 | 0 | 1 | 0 | 0 | 1 | 1 | 0 | 0 | 0 | 1 | 1 | 0 |
| CV16 | 25 | 0 | 0 | 1 | 0 | 0 | 0 | 0 | 0 | 0 | 0 | 0 | 0 | 1 | 0 | 0 | 0 | 1 | 1 | 0 |
| CV161 | 669 | 0 | 0 | 1 | 0 | 0 | 0 | 0 | 0 | 0 | 0 | 0 | 0 | 1 | 0 | 0 | 0 | 1 | 1 | 0 |
| CV164 | 15 | 0 | 0 | 0 | 0 | 0 | 0 | 0 | 0 | 0 | 0 | 0 | 0 | 1 | 0 | 0 | 0 | 1 | 1 | 0 |
| CV168 | 25 | 0 | 0 | 0 | 0 | 0 | 0 | 0 | 0 | 0 | 0 | 0 | 0 | 1 | 0 | 0 | 0 | 1 | 1 | 0 |
| CV169 | 45 | 0 | 0 | 0 | 0 | 0 | 0 | 0 | 0 | 0 | 0 | 0 | 0 | 1 | 0 | 0 | 0 | 1 | 1 | 0 |
| CV17 | 15 | 0 | 0 | 0 | 0 | 0 | 0 | 0 | 0 | 0 | 0 | 0 | 0 | 1 | 0 | 0 | 0 | 1 | 1 | 0 |
| CV173 | 669 | 0 | 0 | 1 | 0 | 0 | 0 | 0 | 0 | 0 | 0 | 0 | 0 | 1 | 0 | 0 | 0 | 1 | 1 | 0 |
| CV174 | 508 | 0 | 0 | 0 | 0 | 0 | 0 | 0 | 0 | 0 | 0 | 0 | 0 | 1 | 0 | 0 | 0 | 1 | 1 | 0 |
| CV178 | 15 | 0 | 0 | 0 | 0 | 0 | 0 | 0 | 0 | 0 | 0 | 0 | 1 | 1 | 0 | 0 | 0 | 1 | 1 | 0 |
| CV179 | 15 | 0 | 0 | 0 | 0 | 0 | 0 | 0 | 0 | 0 | 0 | 0 | 1 | 1 | 0 | 0 | 0 | 1 | 1 | 0 |
| CV18 | 45 | 0 | 0 | 0 | 0 | 0 | 0 | 0 | 0 | 0 | 0 | 0 | 0 | 1 | 0 | 0 | 0 | 1 | 1 | 0 |
| CV185A | 97 | 0 | 0 | 0 | 0 | 0 | 0 | 0 | 0 | 0 | 0 | 0 | 0 | 1 | 0 | 0 | 0 | 1 | 1 | 0 |
| CV188 | 88 | 0 | 0 | 0 | 0 | 1 | 1 | 0 | 0 | 0 | 0 | 1 | 0 | 1 | 0 | 0 | 1 | 1 | 1 | 0 |
| CV196 | 152 | 0 | 0 | 0 | 0 | 0 | 0 | 0 | 0 | 0 | 0 | 0 | 0 | 1 | 0 | 0 | 0 | 1 | 1 | 0 |
| CV199 | 72 | 0 | 0 | 0 | 0 | 0 | 0 | 0 | 0 | 0 | 0 | 0 | 0 | 0 | 0 | 0 | 0 | 1 | 1 | 0 |
| CV204 | 188 | 0 | 0 | 0 | 0 | 0 | 0 | 0 | 0 | 0 | 0 | 0 | 0 | 1 | 0 | 0 | 0 | 1 | 1 | 0 |
| CV213 | 6 | 0 | 0 | 0 | 0 | 0 | 0 | 0 | 0 | 0 | 0 | 0 | 0 | 1 | 0 | 0 | 0 | 1 | 1 | 0 |
| CV214 | 5 | 0 | 0 | 0 | 1 | 0 | 0 | 0 | 0 | 0 | 1 | 0 | 0 | 1 | 1 | 0 | 0 | 1 | 1 | 1 |
| CV219 | 152 | 0 | 0 | 0 | 0 | 0 | 0 | 0 | 0 | 0 | 0 | 0 | 0 | 0 | 0 | 0 | 0 | 1 | 1 | 0 |
| CV220A | 508 | 0 | 0 | 0 | 0 | 0 | 0 | 0 | 0 | 0 | 0 | 0 | 0 | 1 | 0 | 0 | 0 | 1 | 1 | 0 |
| CV225 | 5981 | 0 | 0 | 0 | 0 | 0 | 0 | 0 | 0 | 0 | 0 | 0 | 0 | 1 | 0 | 0 | 0 | 1 | 1 | 0 |
| CV233 | 121 | 0 | 0 | 1 | 0 | 0 | 0 | 0 | 0 | 0 | 0 | 0 | 0 | 1 | 0 | 0 | 0 | 1 | 1 | 0 |
| CV239 | 1 | 0 | 0 | 0 | 0 | 0 | 0 | 0 | 0 | 0 | 0 | 0 | 0 | 1 | 0 | 0 | 0 | 1 | 1 | 0 |
| CV241 | 152 | 0 | 0 | 0 | 0 | 0 | 0 | 0 | 0 | 0 | 0 | 0 | 0 | 1 | 0 | 0 | 0 | 1 | 1 | 0 |
| CV254 | 152 | 0 | 0 | 0 | 0 | 0 | 0 | 0 | 0 | 0 | 0 | 0 | 0 | 0 | 0 | 0 | 0 | 1 | 1 | 0 |
| CV261 | 15 | 0 | 0 | 0 | 0 | 0 | 0 | 0 | 0 | 0 | 0 | 0 | 1 | 1 | 0 | 0 | 0 | 1 | 1 | 0 |
| CV262 | 72 | 0 | 0 | 0 | 0 | 0 | 0 | 0 | 0 | 0 | 0 | 0 | 0 | 1 | 0 | 0 | 0 | 1 | 1 | 0 |
| CV266A | 22 | 0 | 0 | 0 | 0 | 0 | 0 | 0 | 0 | 0 | 0 | 0 | 0 | 1 | 0 | 0 | 0 | 1 | 1 | 0 |
| CV270 | 1 | 0 | 0 | 1 | 1 | 0 | 0 | 0 | 0 | 0 | 1 | 0 | 0 | 1 | 1 | 0 | 0 | 1 | 1 | 1 |
| CV274 | 72 | 0 | 0 | 0 | 0 | 0 | 0 | 0 | 0 | 0 | 0 | 0 | 0 | 1 | 0 | 0 | 0 | 1 | 1 | 0 |
| CV275 | 8 | 0 | 0 | 0 | 1 | 1 | 1 | 0 | 0 | 0 | 1 | 0 | 0 | 1 | 1 | 0 | 0 | 1 | 1 | 1 |
| CV280 | 152 | 0 | 0 | 0 | 0 | 0 | 0 | 0 | 0 | 0 | 0 | 0 | 0 | 1 | 0 | 0 | 0 | 1 | 1 | 0 |
| CV29 | 1 | 0 | 0 | 0 | 0 | 0 | 0 | 0 | 0 | 0 | 0 | 0 | 0 | 1 | 0 | 0 | 0 | 1 | 1 | 0 |
| CV290 | 1 | 0 | 0 | 0 | 0 | 0 | 0 | 0 | 0 | 0 | 0 | 0 | 0 | 1 | 0 | 0 | 0 | 1 | 1 | 0 |
| CV307A | 72 | 0 | 0 | 0 | 0 | 0 | 0 | 0 | 0 | 0 | 0 | 0 | 0 | 1 | 0 | 0 | 0 | 1 | 1 | 0 |
| CV314A | 508 | 0 | 0 | 0 | 0 | 0 | 0 | 0 | 0 | 0 | 0 | 0 | 0 | 1 | 0 | 0 | 0 | 1 | 1 | 0 |
| CV320 | 152 | 0 | 0 | 0 | 0 | 0 | 0 | 0 | 0 | 0 | 0 | 0 | 0 | 1 | 0 | 0 | 0 | 1 | 1 | 0 |
| CV327A | 15 | 0 | 0 | 0 | 0 | 0 | 0 | 0 | 0 | 0 | 0 | 0 | 1 | 1 | 0 | 0 | 0 | 1 | 1 | 0 |
| CV330A | 152 | 0 | 0 | 0 | 0 | 0 | 0 | 0 | 0 | 0 | 0 | 0 | 0 | 1 | 0 | 0 | 0 | 1 | 1 | 0 |
| CV337 | 5 | 0 | 0 | 0 | 1 | 1 | 1 | 1 | 0 | 0 | 1 | 1 | 0 | 0 | 1 | 0 | 0 | 1 | 1 | 1 |
| CV34 | 1472 | 0 | 0 | 0 | 0 | 0 | 0 | 0 | 0 | 0 | 0 | 0 | 0 | 1 | 0 | 0 | 0 | 1 | 1 | 0 |
| CV346 | 88 | 0 | 0 | 0 | 0 | 1 | 1 | 0 | 0 | 0 | 0 | 1 | 0 | 1 | 0 | 0 | 1 | 1 | 1 | 0 |
| CV348 | 25 | 0 | 0 | 0 | 1 | 0 | 0 | 0 | 0 | 0 | 0 | 0 | 0 | 1 | 0 | 0 | 0 | 1 | 1 | 0 |
| CV356 | 508 | 0 | 0 | 0 | 0 | 0 | 0 | 0 | 0 | 0 | 0 | 0 | 0 | 1 | 0 | 0 | 0 | 1 | 1 | 0 |
| CV361A | 669 | 0 | 0 | 0 | 0 | 0 | 0 | 0 | 0 | 0 | 0 | 0 | 0 | 1 | 0 | 0 | 0 | 1 | 1 | 0 |
| CV371 | 152 | 0 | 0 | 0 | 0 | 0 | 0 | 0 | 0 | 0 | 0 | 0 | 0 | 1 | 0 | 0 | 0 | 1 | 1 | 0 |
| CV390A | 97 | 0 | 0 | 0 | 0 | 0 | 0 | 0 | 0 | 0 | 0 | 0 | 0 | 1 | 0 | 0 | 0 | 1 | 1 | 0 |
| CV41 | 72 | 0 | 0 | 0 | 0 | 0 | 0 | 0 | 0 | 0 | 0 | 0 | 0 | 1 | 0 | 0 | 0 | 1 | 1 | 0 |
| CV414 | 188 | 0 | 0 | 0 | 0 | 0 | 0 | 0 | 0 | 0 | 0 | 0 | 0 | 1 | 0 | 0 | 0 | 1 | 1 | 0 |
| CV43 | 1 | 0 | 0 | 0 | 0 | 0 | 0 | 0 | 0 | 0 | 0 | 0 | 0 | 1 | 0 | 0 | 0 | 1 | 1 | 0 |
| CV442 | 1472 | 0 | 0 | 0 | 0 | 0 | 0 | 0 | 0 | 0 | 0 | 0 | 0 | 1 | 0 | 0 | 0 | 1 | 1 | 0 |
| CV443 | 188 | 0 | 0 | 0 | 0 | 0 | 0 | 0 | 0 | 0 | 0 | 0 | 0 | 0 | 0 | 0 | 0 | 1 | 1 | 0 |
| CV462 | 1472 | 0 | 0 | 0 | 0 | 0 | 0 | 0 | 0 | 0 | 0 | 0 | 0 | 1 | 0 | 0 | 0 | 1 | 1 | 0 |
| CV464 | 398 | 0 | 0 | 0 | 0 | 0 | 0 | 0 | 1 | 0 | 0 | 0 | 0 | 1 | 0 | 0 | 0 | 1 | 1 | 0 |
| CV476 | 2498 | 0 | 0 | 0 | 0 | 0 | 0 | 0 | 0 | 0 | 0 | 0 | 0 | 1 | 0 | 0 | 0 | 1 | 1 | 0 |
| CV479 | 152 | 0 | 0 | 0 | 0 | 0 | 0 | 0 | 0 | 0 | 0 | 0 | 0 | 1 | 0 | 0 | 0 | 1 | 1 | 0 |
| CV485 | 5 | 0 | 0 | 0 | 1 | 0 | 0 | 0 | 0 | 0 | 1 | 0 | 0 | 1 | 1 | 0 | 0 | 1 | 1 | 1 |
| CV492 | 5 | 0 | 0 | 0 | 1 | 1 | 1 | 1 | 0 | 0 | 1 | 1 | 0 | 0 | 1 | 0 | 0 | 1 | 1 | 1 |
| CV495 | 5 | 0 | 0 | 0 | 1 | 1 | 1 | 1 | 0 | 0 | 1 | 1 | 0 | 0 | 1 | 0 | 0 | 1 | 1 | 1 |
| CV496 | 1472 | 0 | 0 | 0 | 0 | 0 | 0 | 0 | 0 | 0 | 0 | 0 | 0 | 1 | 0 | 0 | 0 | 1 | 1 | 0 |
| CV500A | 1 | 0 | 0 | 0 | 0 | 0 | 0 | 1 | 0 | 0 | 0 | 0 | 0 | 1 | 0 | 0 | 0 | 1 | 1 | 0 |
| CV516A | 152 | 0 | 0 | 0 | 0 | 0 | 0 | 0 | 0 | 0 | 0 | 0 | 0 | 1 | 0 | 0 | 0 | 1 | 1 | 0 |
| CV531A | 4996 | 0 | 0 | 0 | 0 | 0 | 0 | 0 | 0 | 0 | 0 | 0 | 0 | 1 | 0 | 0 | 0 | 1 | 1 | 0 |
| CV532 | 45 | 0 | 0 | 0 | 0 | 0 | 0 | 0 | 0 | 0 | 0 | 0 | 0 | 1 | 0 | 0 | 0 | 1 | 1 | 0 |
| CV533 | 5 | 0 | 0 | 0 | 1 | 0 | 0 | 0 | 0 | 0 | 1 | 0 | 0 | 1 | 1 | 0 | 0 | 1 | 1 | 1 |
| CV534 | 15 | 0 | 0 | 0 | 0 | 0 | 0 | 0 | 0 | 0 | 0 | 0 | 0 | 1 | 0 | 0 | 0 | 1 | 1 | 0 |
| CV540 | 273 | 0 | 0 | 0 | 1 | 0 | 0 | 0 | 0 | 0 | 1 | 0 | 0 | 1 | 1 | 0 | 0 | 1 | 1 | 1 |
| CV548 | 152 | 0 | 0 | 0 | 0 | 0 | 0 | 0 | 0 | 0 | 0 | 0 | 0 | 1 | 0 | 0 | 0 | 1 | 1 | 0 |
| CV55 | 291 | 0 | 0 | 0 | 0 | 0 | 0 | 0 | 0 | 0 | 0 | 0 | 0 | 1 | 0 | 0 | 0 | 1 | 1 | 0 |
| CV558 | 1472 | 0 | 0 | 0 | 0 | 0 | 0 | 0 | 0 | 0 | 0 | 0 | 0 | 1 | 0 | 0 | 0 | 1 | 1 | 0 |
| CV561 | 15 | 0 | 0 | 0 | 0 | 0 | 0 | 0 | 0 | 0 | 0 | 0 | 1 | 1 | 0 | 0 | 0 | 1 | 1 | 0 |
| CV562A | 15 | 0 | 0 | 0 | 0 | 0 | 0 | 0 | 0 | 0 | 0 | 0 | 1 | 1 | 0 | 0 | 0 | 1 | 1 | 0 |
| CV573 | 508 | 0 | 0 | 0 | 0 | 0 | 0 | 0 | 0 | 0 | 0 | 0 | 0 | 1 | 0 | 0 | 0 | 1 | 1 | 0 |
| CV576 | 22 | 0 | 0 | 0 | 0 | 0 | 0 | 0 | 0 | 0 | 0 | 0 | 0 | 1 | 0 | 0 | 0 | 1 | 1 | 0 |
| CV58 | 2021 | 0 | 0 | 0 | 0 | 0 | 0 | 0 | 0 | 0 | 0 | 0 | 0 | 1 | 0 | 0 | 0 | 1 | 1 | 0 |
| CV581 | 72 | 0 | 0 | 0 | 0 | 0 | 0 | 0 | 0 | 0 | 0 | 0 | 0 | 1 | 0 | 0 | 0 | 1 | 1 | 0 |
| CV582 | 15 | 0 | 0 | 0 | 0 | 0 | 0 | 0 | 0 | 0 | 0 | 0 | 0 | 1 | 0 | 0 | 0 | 1 | 1 | 0 |
| CV587 | 508 | 0 | 0 | 0 | 0 | 0 | 0 | 0 | 0 | 0 | 0 | 0 | 0 | 1 | 0 | 0 | 0 | 1 | 1 | 0 |
| CV591A | 45 | 0 | 0 | 0 | 0 | 0 | 0 | 0 | 0 | 0 | 0 | 0 | 0 | 1 | 0 | 0 | 0 | 1 | 1 | 0 |
| CV595 | 22 | 0 | 0 | 0 | 0 | 0 | 0 | 0 | 0 | 0 | 0 | 0 | 0 | 1 | 0 | 0 | 0 | 1 | 1 | 0 |
| CV603 | 97 | 0 | 0 | 0 | 0 | 0 | 0 | 0 | 0 | 0 | 0 | 0 | 0 | 1 | 0 | 0 | 0 | 1 | 1 | 0 |
| CV625A | 273 | 0 | 0 | 0 | 1 | 0 | 0 | 0 | 0 | 0 | 1 | 0 | 0 | 1 | 1 | 0 | 0 | 1 | 1 | 1 |
| CV627 | 22 | 0 | 0 | 0 | 0 | 0 | 0 | 0 | 0 | 0 | 0 | 0 | 0 | 1 | 0 | 0 | 0 | 1 | 1 | 0 |
| CV634 | 152 | 0 | 0 | 0 | 0 | 0 | 0 | 0 | 0 | 0 | 0 | 0 | 0 | 0 | 0 | 0 | 0 | 1 | 1 | 0 |
| CV638 | 508 | 0 | 0 | 0 | 0 | 0 | 0 | 0 | 0 | 0 | 0 | 0 | 0 | 1 | 0 | 0 | 0 | 1 | 1 | 0 |
| CV648 | 22 | 0 | 0 | 0 | 0 | 0 | 0 | 0 | 0 | 0 | 0 | 0 | 0 | 1 | 0 | 0 | 0 | 1 | 1 | 0 |
| CV654 | 5 | 0 | 0 | 0 | 1 | 0 | 0 | 0 | 0 | 0 | 1 | 0 | 0 | 1 | 1 | 0 | 0 | 1 | 1 | 1 |
| CV66 | 5 | 0 | 0 | 0 | 1 | 0 | 0 | 0 | 0 | 0 | 1 | 0 | 0 | 1 | 1 | 0 | 0 | 1 | 1 | 1 |
| CV669A | 15 | 0 | 0 | 0 | 0 | 0 | 0 | 0 | 0 | 0 | 0 | 0 | 1 | 1 | 0 | 0 | 0 | 1 | 1 | 0 |
| CV693 | 5 | 0 | 0 | 0 | 1 | 1 | 1 | 1 | 0 | 0 | 1 | 1 | 0 | 0 | 1 | 0 | 0 | 1 | 1 | 1 |
| CV81 | 508 | 0 | 0 | 0 | 0 | 0 | 0 | 0 | 0 | 0 | 0 | 0 | 0 | 1 | 0 | 0 | 0 | 1 | 1 | 0 |
| CV85 | 669 | 0 | 0 | 0 | 0 | 0 | 0 | 0 | 0 | 0 | 0 | 0 | 0 | 1 | 0 | 0 | 0 | 1 | 1 | 0 |
| CV87 | 668 | 1 | 0 | 0 | 0 | 0 | 0 | 0 | 0 | 0 | 0 | 0 | 0 | 1 | 0 | 0 | 0 | 1 | 1 | 0 |

0 – absence; 1 – presence

ST – sequence type

*tetM*, *tetL*, *tetK* – tetracycline resistance; *msrA* – erythromycin resistance; *mecA*, *mecR1* – methicillin resistance; *fusC* – fusidic acid resistance; *ermT*, *ermC*, *mphC* – erythron- and clindamycin resistance; *dfrC*, *dfrG* – trimethoprim resistance; *blaZ* – penicillin resistance; *aph(3’)-III* – kanamycin and neomycin resistance; *cfrC* – chloramphenicol resistance; *vgaA* – streptogramin A resistance; *mepA*, *mep1* – tigecycline resistance; *sat4* – streptothricin resistance

Table S4: Phenotypic antimicrobial susceptibilities of *S. aureus* isolates from Cape Verde

| Isolate | **ST** | **TE** | **OX** | **FOX** | **FD** | **E** | **DA** | **P** | **C** | **RD** | **CIP** | **CN** | **TEC** | **MUP** | **SXT** | **QD** | **LZD** | **VA** |
| --- | --- | --- | --- | --- | --- | --- | --- | --- | --- | --- | --- | --- | --- | --- | --- | --- | --- | --- |
| CV107 | 669 | S | S | S | S | S | S | R | S | S | S | S | S | I | S | S | S | S |
| CV109 | 121 | R | S | S | S | S | S | R | S | S | S | S | S | I | S | S | S | S |
| CV11 | 30 | R | S | S | S | R | R | R | S | S | S | S | S | I | S | S | S | S |
| CV110 | 669 | S | S | S | S | S | S | R | S | S | S | S | S | S | S | S | S | S |
| CV115 | 30 | S | S | S | S | S | S | R | S | S | S | S | S | S | S | S | S | S |
| CV120 | 30 | R | S | S | S | S | S | R | S | S | S | S | S | S | S | S | S | S |
| CV126 | 8 | S | S | S | S | S | S | R | S | S | S | S | S | I | S | S | S | S |
| CV133 | 121 | R | S | S | S | S | S | R | S | S | S | S | S | S | S | S | S | S |
| CV141 | 8 | R | S | S | S | S | S | R | S | S | S | S | S | I | S | S | S | S |
| CV144 | 5980 | R | S | S | S | S | S | R | S | S | S | S | S | S | S | S | S | S |
| CV145 | 669 | S | S | S | S | S | S | R | S | R | S | S | S | S | S | S | S | S |
| CV15 | 121 | R | S | S | S | S | S | R | S | S | S | S | S | I | S | S | S | S |
| CV151 | 2300 | S | S | S | S | R | R | R | S | S | S | S | S | I | S | S | S | S |
| CV16 | 25 | R | S | S | S | S | S | R | S | S | S | S | S | S | S | S | S | S |
| CV161 | 669 | R | S | S | S | S | S | R | S | S | S | S | S | S | S | S | S | S |
| CV164 | 15 | S | S | S | S | S | S | R | S | S | S | S | S | I | S | S | S | S |
| CV168 | 25 | S | S | S | S | S | S | R | S | S | S | S | S | S | S | S | S | S |
| CV169 | 45 | S | S | S | S | S | S | R | S | S | S | S | S | I | S | S | S | S |
| CV17 | 15 | S | S | S | S | S | S | R | S | S | S | S | S | I | S | S | S | S |
| CV173 | 669 | R | S | S | S | S | S | R | S | S | S | S | S | I | S | S | S | S |
| CV174 | 508 | S | S | S | S | S | S | R | S | S | S | S | S | S | S | S | S | S |
| CV178 | 15 | S | S | S | S | S | S | R | S | S | S | S | S | S | S | S | S | S |
| CV179 | 15 | S | S | S | S | S | S | R | S | S | S | S | S | S | S | S | S | S |
| CV18 | 45 | S | S | S | S | S | S | R | S | S | S | S | S | I | S | S | S | S |
| CV185A | 97 | S | S | S | S | S | S | R | S | S | S | S | S | S | S | S | S | S |
| CV188 | 88 | S | R | R | S | S | S | R | S | S | S | S | S | S | R | S | S | S |
| CV196 | 152 | S | S | S | S | S | S | R | S | S | S | S | S | S | S | S | S | S |
| CV199 | 72 | S | S | S | S | S | S | S | S | S | S | S | S | S | S | S | S | S |
| CV204 | 188 | S | S | S | S | S | S | R | S | S | S | S | S | S | S | S | S | S |
| CV213 | 6 | S | S | S | S | S | S | R | S | S | S | S | S | S | S | S | S | S |
| CV214 | 5 | S | S | S | S | R | S | R | S | S | S | S | S | S | S | S | S | S |
| CV219 | 152 | S | S | S | S | S | S | S | S | S | S | S | S | S | S | S | S | S |
| CV220A | 508 | S | S | S | S | S | S | R | S | S | S | S | S | S | S | S | S | S |
| CV225 | 5981 | S | S | S | S | S | S | R | S | S | S | S | S | S | S | S | S | S |
| CV233 | 121 | R | S | S | S | S | S | R | S | S | S | S | S | S | S | S | S | S |
| CV239 | 1 | S | S | S | S | S | S | R | S | S | S | S | S | S | S | S | S | S |
| CV241 | 152 | S | S | S | S | S | S | R | S | S | S | S | S | S | S | S | S | S |
| CV254 | 152 | S | S | S | S | S | S | S | S | S | S | S | S | S | S | S | S | S |
| CV261 | 15 | S | S | S | S | S | S | R | S | S | S | S | S | S | R | S | S | S |
| CV262 | 72 | S | S | S | S | S | S | R | S | S | S | S | S | S | S | S | S | S |
| CV266A | 22 | S | S | S | S | S | S | R | S | S | S | S | S | S | S | S | S | S |
| CV270 | 1 | R | S | S | S | R | S | R | S | S | R | S | S | S | S | S | S | S |
| CV274 | 72 | S | S | S | S | S | S | R | S | S | S | S | S | S | S | S | S | S |
| CV275 | 8 | S | R | R | S | R | S | R | S | S | R | S | S | S | S | S | S | S |
| CV280 | 152 | S | S | S | S | S | S | R | S | S | S | S | S | S | S | S | S | S |
| CV29 | 1 | S | S | S | S | S | S | R | S | S | S | S | S | I | S | S | S | S |
| CV290 | 1 | S | S | S | S | S | S | R | S | S | S | S | S | S | S | S | S | S |
| CV307A | 72 | S | S | S | S | S | S | R | S | S | S | S | S | S | S | S | S | S |
| CV314A | 508 | S | S | S | S | S | S | R | S | S | S | S | S | S | S | S | S | S |
| CV320 | 152 | S | S | S | S | S | S | R | S | S | S | S | S | S | S | S | S | S |
| CV327A | 15 | S | S | S | S | S | S | R | S | S | S | S | S | S | R | S | S | S |
| CV330A | 152 | S | S | S | S | S | S | R | S | S | S | S | S | S | S | S | S | S |
| CV337 | 5 | S | R | R | R | R | R | R | S | S | S | S | S | S | S | S | S | S |
| CV34 | 1472 | S | S | S | S | S | S | R | S | S | S | S | S | S | S | S | S | S |
| CV346 | 88 | S | R | R | S | S | S | R | S | S | S | S | S | S | R | S | S | S |
| CV348 | 25 | S | S | S | S | R | S | R | S | S | S | S | S | S | S | S | S | S |
| CV356 | 508 | S | S | S | S | S | S | R | S | S | S | S | S | S | S | S | S | S |
| CV361A | 669 | S | S | S | S | S | S | R | S | S | S | S | S | S | S | S | S | S |
| CV371 | 152 | S | S | S | S | S | S | R | S | S | S | S | S | S | S | S | S | S |
| CV390A | 97 | S | S | S | S | S | S | R | S | S | S | S | S | S | S | S | S | S |
| CV41 | 72 | S | S | S | S | S | S | R | S | S | S | S | S | I | S | S | S | S |
| CV414 | 188 | S | S | S | S | S | S | R | S | S | S | S | S | S | S | S | S | S |
| CV43 | 1 | S | S | S | S | S | S | R | S | S | S | S | S | S | S | S | S | S |
| CV442 | 1472 | S | S | S | S | S | S | R | S | S | S | S | S | S | S | S | S | S |
| CV443 | 188 | S | S | S | S | S | S | S | S | S | S | S | S | S | S | S | S | S |
| CV462 | 1472 | S | S | S | S | S | S | R | S | S | S | S | S | S | S | S | S | S |
| CV464 | 398 | S | S | S | S | R | R | R | S | S | S | S | S | S | S | S | S | S |
| CV476 | 2498 | S | S | S | S | S | S | R | S | S | S | S | S | S | S | S | S | S |
| CV479 | 152 | S | S | S | S | S | S | R | S | S | S | S | S | S | S | S | S | S |
| CV485 | 5 | S | S | S | S | R | S | R | S | S | S | S | S | S | S | S | S | S |
| CV492 | 5 | S | R | R | R | R | S | R | S | S | I | S | S | S | R | S | S | S |
| CV495 | 5 | S | R | R | R | R | S | R | S | S | I | S | S | S | R | S | S | S |
| CV496 | 1472 | S | S | S | S | S | S | R | S | S | S | S | S | S | S | S | S | S |
| CV500A | 1 | S | S | S | R | S | S | R | S | S | S | S | S | S | S | S | S | S |
| CV516A | 152 | S | S | S | S | S | S | R | S | S | S | S | S | S | S | S | S | S |
| CV531A | 4996 | S | S | S | S | S | S | R | S | S | S | S | S | S | S | S | S | S |
| CV532 | 45 | S | S | S | S | S | S | R | S | S | S | S | S | S | S | S | S | S |
| CV533 | 5 | S | S | S | S | R | S | R | S | S | S | S | S | S | S | S | S | S |
| CV534 | 15 | S | S | S | S | S | S | R | S | S | S | S | S | S | S | S | S | S |
| CV540 | 273 | S | S | S | S | R | S | R | S | S | S | S | S | S | S | S | S | S |
| CV548 | 152 | S | S | S | S | S | S | R | S | S | S | S | S | S | S | S | S | S |
| CV55 | 291 | S | S | S | S | S | S | R | S | S | S | S | S | I | S | S | S | S |
| CV558 | 1472 | S | S | S | S | S | S | R | S | S | S | S | S | S | S | S | S | S |
| CV561 | 15 | S | S | S | S | S | S | R | S | S | S | S | S | R | R | S | S | S |
| CV562A | 15 | S | S | S | S | S | S | R | S | S | S | S | S | S | R | S | S | S |
| CV573 | 508 | S | S | S | S | S | S | R | S | S | S | S | S | S | S | S | S | S |
| CV576 | 22 | S | S | S | S | S | S | R | S | S | S | S | S | S | S | S | S | S |
| CV58 | 2021 | S | S | S | S | S | S | R | S | S | S | S | S | S | S | S | S | S |
| CV581 | 72 | S | S | S | S | S | S | R | S | S | S | S | S | S | S | S | S | S |
| CV582 | 15 | S | S | S | S | S | S | R | S | S | S | S | S | R | S | S | S | S |
| CV587 | 508 | S | S | S | S | S | S | R | S | S | S | S | S | S | S | S | S | S |
| CV591A | 45 | S | S | S | S | S | S | R | S | S | S | S | S | S | S | S | S | S |
| CV595 | 22 | S | S | S | S | S | S | R | S | S | S | S | S | S | S | S | S | S |
| CV603 | 97 | S | S | S | S | S | S | R | S | S | S | S | S | S | S | S | S | S |
| CV625A | 273 | S | S | S | S | R | S | R | S | S | S | S | S | R | S | S | S | S |
| CV627 | 22 | S | S | S | S | S | S | R | S | S | S | S | S | S | S | S | S | S |
| CV634 | 152 | S | S | S | S | S | S | S | S | S | S | S | S | S | S | S | S | S |
| CV638 | 508 | S | S | S | S | S | S | R | S | S | S | S | S | R | S | S | S | S |
| CV648 | 22 | S | S | S | S | S | S | R | S | S | S | S | S | S | S | S | S | S |
| CV654 | 5 | S | S | S | S | R | S | R | S | S | S | S | S | S | S | S | S | S |
| CV66 | 5 | S | S | S | S | R | S | R | S | S | S | S | S | I | S | S | S | S |
| CV669A | 15 | S | S | S | S | S | S | R | S | S | S | S | S | R | R | S | S | S |
| CV693 | 5 | S | R | R | R | R | S | S | S | S | S | S | S | S | S | S | S | S |
| CV81 | 508 | S | S | S | S | S | S | R | S | S | S | S | S | I | S | S | S | S |
| CV85 | 669 | S | S | S | S | S | S | R | S | S | S | S | S | I | S | S | S | S |
| CV87 | 668 | R | S | S | S | S | S | R | S | S | S | S | S | S | S | S | S | S |

ST – sequence type

R – resistant; S – susceptible; I – intermediate resistance according to EUCAST guidelines.

TE – tetracycline; OX – oxacillin; FOX – cefoxitin; FD – fusidic acid; E – erythromycin; DA – clindamycin; P – penicillin; C – chloramphenicol; RD – rifampicin; CIP – ciprofloxacin; CN – gentamicin; TEC – teicoplanin; MUP – mupirocin; SXT - trimethoprim-sulfamethoxazole; QD - quinupristin-dalfopristin; LZD – linezolid; VA - vancomycin

Table S5: Plasmid replicons types identified in *S. aureus* isolates from Cape Verde

| **Isolate** | **ST** | ***blaZ*** | **rep10_3_ORF(pNE131)** | **rep13_3_rep(pWBG1773)** | **rep13_4_rep(pKH13)** | **rep16_1_CDS8(pSAS)** | **rep16_2_CDS6(pSJH101)** | **rep16_3_rep(Saa6159)** | **rep16_4_unknown(SAP056A)** | **rep16_7_rep(pBORa53)** | **rep19_10_rep(pWBG746)** | **rep19_3_CDS20(pSJH901)** | **rep19_7_repA(SAP019A)** | **rep20_1_ORF1(EDINA)** | **rep20_3_rep(pTW20)** | **rep21_10_rep(pKH14)** | **rep21_11_rep(pSA1308)** | **rep24_1_rep(pWBG745)** | **rep5_1_rep(pMW2)** | **rep5_4_rep(SAP047A)** | **rep5_6_rep(pRJ9)** | **rep7_14_rep(MSSA476)** | **rep7_1_repC(Cassette)** | **repUS12__rep(pUB110)** | **repUS5__CDS20(pETB)** |
| --- | --- | --- | --- | --- | --- | --- | --- | --- | --- | --- | --- | --- | --- | --- | --- | --- | --- | --- | --- | --- | --- | --- | --- | --- | --- |
| **CV214** | **5** | 1 | 0 | 0 | 0 | 0 | 0 | 0 | 0 | 0 | 0 | 0 | 0 | 0 | 0 | 0 | 0 | 0 | 0 | 1 | 0 | 0 | 0 | 0 | 0 |
| **CV337** | **5** | 0 | 0 | 0 | 0 | 0 | 0 | 0 | 0 | 0 | 0 | 0 | 0 | 0 | 0 | 0 | 0 | 0 | 0 | 0 | 0 | 0 | 0 | 0 | 0 |
| **CV485** | **5** | 1 | 0 | 1 | 0 | 0 | 0 | 0 | 0 | 0 | 0 | 0 | 0 | 0 | 0 | 0 | 0 | 0 | 0 | 1 | 0 | 0 | 0 | 0 | 0 |
| **CV492** | **5** | 0 | 0 | 0 | 0 | 0 | 0 | 0 | 0 | 0 | 0 | 0 | 0 | 0 | 0 | 0 | 0 | 0 | 0 | 0 | 0 | 0 | 0 | 0 | 0 |
| **CV495** | **5** | 0 | 0 | 0 | 0 | 0 | 0 | 0 | 0 | 0 | 0 | 0 | 0 | 0 | 0 | 0 | 0 | 0 | 0 | 0 | 0 | 0 | 0 | 0 | 0 |
| **CV533** | **5** | 1 | 0 | 0 | 0 | 0 | 0 | 0 | 0 | 0 | 0 | 0 | 0 | 0 | 0 | 0 | 0 | 0 | 0 | 1 | 0 | 0 | 0 | 0 | 0 |
| **CV654** | **5** | 1 | 0 | 0 | 0 | 0 | 0 | 0 | 0 | 0 | 0 | 0 | 0 | 0 | 0 | 0 | 0 | 0 | 0 | 1 | 0 | 0 | 0 | 0 | 0 |
| **CV66** | **5** | 1 | 0 | 0 | 0 | 0 | 0 | 0 | 0 | 0 | 0 | 0 | 0 | 0 | 0 | 0 | 0 | 0 | 0 | 1 | 0 | 0 | 0 | 0 | 0 |
| **CV693** | **5** | 0 | 0 | 0 | 0 | 0 | 0 | 0 | 0 | 0 | 0 | 0 | 0 | 0 | 0 | 0 | 0 | 0 | 0 | 0 | 0 | 0 | 0 | 0 | 0 |
| **CV199** | **72** | 0 | 0 | 0 | 0 | 0 | 0 | 0 | 0 | 0 | 0 | 0 | 0 | 0 | 0 | 0 | 0 | 0 | 0 | 0 | 0 | 1 | 0 | 0 | 0 |
| **CV262** | **72** | 1 | 0 | 0 | 0 | 0 | 0 | 0 | 0 | 0 | 0 | 0 | 0 | 0 | 0 | 0 | 1 | 0 | 0 | 0 | 0 | 1 | 0 | 0 | 0 |
| **CV274** | **72** | 1 | 0 | 0 | 0 | 0 | 0 | 0 | 0 | 0 | 0 | 0 | 0 | 0 | 0 | 0 | 0 | 0 | 0 | 0 | 0 | 1 | 0 | 0 | 0 |
| **CV307A** | **72** | 1 | 0 | 0 | 0 | 0 | 0 | 0 | 0 | 0 | 0 | 0 | 0 | 0 | 0 | 0 | 0 | 0 | 0 | 0 | 0 | 1 | 0 | 0 | 0 |
| **CV41** | **72** | 1 | 0 | 0 | 0 | 0 | 0 | 0 | 0 | 0 | 0 | 0 | 0 | 0 | 0 | 0 | 1 | 0 | 0 | 0 | 0 | 1 | 0 | 0 | 0 |
| **CV581** | **72** | 1 | 0 | 0 | 0 | 0 | 0 | 0 | 0 | 0 | 0 | 0 | 0 | 0 | 0 | 1 | 0 | 0 | 0 | 0 | 0 | 1 | 0 | 0 | 0 |
| **CV196** | **152** | 1 | 0 | 0 | 0 | 0 | 0 | 1 | 0 | 0 | 0 | 0 | 0 | 0 | 0 | 0 | 0 | 0 | 1 | 0 | 0 | 0 | 0 | 0 | 0 |
| **CV219** | **152** | 0 | 0 | 0 | 0 | 0 | 0 | 0 | 0 | 0 | 0 | 0 | 0 | 0 | 0 | 0 | 0 | 0 | 0 | 0 | 0 | 0 | 0 | 0 | 0 |
| **CV241** | **152** | 1 | 0 | 0 | 0 | 0 | 0 | 1 | 0 | 0 | 0 | 0 | 0 | 0 | 0 | 0 | 0 | 0 | 1 | 0 | 0 | 0 | 0 | 0 | 0 |
| **CV254** | **152** | 0 | 0 | 0 | 0 | 0 | 0 | 0 | 0 | 0 | 0 | 0 | 0 | 0 | 0 | 0 | 0 | 0 | 0 | 0 | 0 | 0 | 0 | 0 | 0 |
| **CV280** | **152** | 1 | 0 | 1 | 0 | 0 | 0 | 1 | 0 | 0 | 0 | 0 | 0 | 0 | 0 | 0 | 0 | 0 | 1 | 0 | 0 | 0 | 0 | 0 | 0 |
| **CV320** | **152** | 1 | 0 | 0 | 0 | 0 | 0 | 1 | 0 | 0 | 0 | 0 | 0 | 0 | 0 | 0 | 0 | 0 | 1 | 0 | 0 | 0 | 0 | 0 | 0 |
| **CV330A** | **152** | 1 | 0 | 0 | 0 | 0 | 0 | 1 | 0 | 0 | 0 | 0 | 0 | 0 | 0 | 0 | 0 | 0 | 1 | 0 | 0 | 0 | 0 | 0 | 0 |
| **CV371** | **152** | 1 | 0 | 0 | 0 | 0 | 0 | 1 | 0 | 0 | 0 | 0 | 0 | 0 | 0 | 0 | 0 | 0 | 1 | 0 | 0 | 0 | 0 | 0 | 0 |
| **CV479** | **152** | 1 | 0 | 1 | 0 | 0 | 0 | 1 | 0 | 0 | 0 | 0 | 0 | 0 | 0 | 0 | 0 | 0 | 1 | 0 | 0 | 0 | 0 | 0 | 0 |
| **CV516A** | **152** | 1 | 0 | 1 | 0 | 0 | 0 | 1 | 0 | 0 | 0 | 0 | 0 | 0 | 0 | 0 | 0 | 0 | 1 | 0 | 0 | 0 | 0 | 0 | 0 |
| **CV548** | **152** | 1 | 0 | 0 | 0 | 0 | 0 | 1 | 0 | 0 | 0 | 0 | 0 | 0 | 0 | 0 | 0 | 0 | 1 | 0 | 0 | 0 | 0 | 0 | 0 |
| **CV634** | **152** | 0 | 0 | 0 | 0 | 0 | 0 | 0 | 0 | 0 | 0 | 0 | 0 | 0 | 0 | 0 | 0 | 0 | 0 | 0 | 0 | 0 | 0 | 0 | 0 |
| **CV204** | **188** | 1 | 0 | 0 | 0 | 0 | 0 | 1 | 0 | 0 | 0 | 0 | 0 | 0 | 0 | 0 | 0 | 0 | 1 | 0 | 0 | 0 | 0 | 0 | 0 |
| **CV414** | **188** | 1 | 0 | 0 | 0 | 0 | 0 | 1 | 0 | 0 | 0 | 0 | 0 | 0 | 0 | 0 | 0 | 0 | 1 | 0 | 0 | 0 | 0 | 0 | 0 |
| **CV443** | **188** | 0 | 0 | 0 | 0 | 0 | 0 | 0 | 0 | 0 | 0 | 0 | 0 | 0 | 0 | 0 | 0 | 1 | 0 | 0 | 0 | 0 | 0 | 0 | 0 |
| CV239 | 1 | 1 | 0 | 0 | 0 | 1 | 0 | 0 | 0 | 0 | 0 | 0 | 0 | 0 | 0 | 0 | 0 | 0 | 1 | 0 | 0 | 1 | 0 | 0 | 0 |
| CV270 | 1 | 1 | 0 | 0 | 0 | 0 | 0 | 0 | 0 | 0 | 0 | 0 | 0 | 0 | 1 | 0 | 0 | 0 | 0 | 0 | 0 | 1 | 0 | 0 | 0 |
| CV29 | 1 | 1 | 0 | 0 | 0 | 1 | 0 | 0 | 0 | 0 | 0 | 0 | 0 | 0 | 0 | 0 | 0 | 0 | 1 | 0 | 0 | 1 | 0 | 0 | 0 |
| CV290 | 1 | 1 | 0 | 0 | 0 | 1 | 0 | 0 | 0 | 0 | 0 | 0 | 0 | 0 | 0 | 0 | 0 | 0 | 1 | 0 | 0 | 1 | 0 | 0 | 0 |
| CV43 | 1 | 1 | 0 | 0 | 0 | 1 | 0 | 0 | 0 | 0 | 0 | 0 | 0 | 0 | 0 | 0 | 0 | 1 | 1 | 0 | 0 | 1 | 0 | 0 | 0 |
| CV500A | 1 | 1 | 0 | 1 | 0 | 1 | 0 | 0 | 0 | 0 | 0 | 0 | 0 | 0 | 0 | 0 | 0 | 0 | 1 | 0 | 0 | 1 | 0 | 0 | 0 |
| CV213 | 6 | 1 | 0 | 0 | 0 | 0 | 0 | 1 | 0 | 0 | 0 | 0 | 0 | 0 | 0 | 0 | 0 | 0 | 1 | 0 | 0 | 0 | 0 | 0 | 0 |
| CV126 | 8 | 1 | 0 | 0 | 0 | 0 | 0 | 0 | 0 | 0 | 0 | 0 | 0 | 0 | 1 | 0 | 0 | 0 | 0 | 0 | 0 | 1 | 0 | 0 | 0 |
| CV141 | 8 | 1 | 0 | 0 | 0 | 0 | 0 | 0 | 0 | 0 | 0 | 0 | 0 | 0 | 1 | 0 | 0 | 0 | 0 | 0 | 0 | 1 | 1 | 0 | 0 |
| CV275 | 8 | 1 | 0 | 0 | 0 | 0 | 1 | 0 | 0 | 0 | 0 | 1 | 0 | 0 | 0 | 0 | 0 | 0 | 0 | 0 | 0 | 1 | 0 | 0 | 0 |
| CV164 | 15 | 1 | 0 | 0 | 0 | 0 | 0 | 1 | 0 | 0 | 0 | 0 | 0 | 0 | 0 | 0 | 0 | 0 | 1 | 0 | 0 | 0 | 0 | 0 | 0 |
| CV17 | 15 | 1 | 0 | 0 | 0 | 0 | 0 | 1 | 0 | 0 | 0 | 0 | 0 | 0 | 0 | 0 | 0 | 0 | 1 | 0 | 0 | 0 | 0 | 0 | 0 |
| CV178 | 15 | 1 | 0 | 0 | 0 | 0 | 0 | 1 | 0 | 0 | 0 | 0 | 0 | 0 | 0 | 0 | 0 | 0 | 1 | 0 | 0 | 0 | 0 | 0 | 0 |
| CV179 | 15 | 1 | 0 | 0 | 0 | 0 | 0 | 1 | 0 | 0 | 0 | 0 | 0 | 0 | 0 | 0 | 0 | 0 | 1 | 0 | 0 | 0 | 0 | 0 | 0 |
| CV261 | 15 | 1 | 0 | 0 | 0 | 0 | 0 | 1 | 0 | 0 | 0 | 0 | 0 | 0 | 0 | 0 | 0 | 0 | 1 | 0 | 0 | 0 | 0 | 0 | 0 |
| CV327A | 15 | 1 | 0 | 0 | 0 | 0 | 0 | 1 | 0 | 0 | 0 | 0 | 0 | 0 | 0 | 0 | 0 | 0 | 1 | 0 | 0 | 0 | 0 | 0 | 0 |
| CV534 | 15 | 1 | 0 | 0 | 0 | 0 | 0 | 1 | 0 | 0 | 0 | 0 | 0 | 0 | 0 | 0 | 0 | 0 | 1 | 0 | 0 | 0 | 0 | 0 | 0 |
| CV561 | 15 | 1 | 0 | 1 | 0 | 0 | 0 | 1 | 0 | 0 | 0 | 0 | 0 | 0 | 0 | 0 | 0 | 0 | 1 | 0 | 0 | 0 | 0 | 0 | 0 |
| CV562A | 15 | 1 | 0 | 0 | 0 | 0 | 0 | 1 | 0 | 0 | 0 | 0 | 0 | 0 | 0 | 0 | 0 | 0 | 1 | 0 | 0 | 0 | 0 | 0 | 0 |
| CV582 | 15 | 1 | 0 | 0 | 0 | 0 | 0 | 1 | 0 | 0 | 0 | 0 | 0 | 0 | 0 | 0 | 0 | 0 | 1 | 0 | 0 | 0 | 0 | 0 | 0 |
| CV669A | 15 | 1 | 0 | 0 | 0 | 0 | 0 | 1 | 0 | 0 | 0 | 0 | 0 | 0 | 0 | 0 | 0 | 0 | 1 | 0 | 0 | 0 | 0 | 0 | 0 |
| CV266A | 22 | 1 | 0 | 1 | 0 | 0 | 0 | 0 | 0 | 0 | 0 | 0 | 0 | 0 | 1 | 0 | 0 | 0 | 1 | 0 | 1 | 0 | 0 | 0 | 0 |
| CV576 | 22 | 1 | 0 | 0 | 0 | 0 | 0 | 0 | 0 | 0 | 0 | 0 | 0 | 0 | 1 | 0 | 0 | 0 | 1 | 0 | 0 | 0 | 0 | 0 | 0 |
| CV595 | 22 | 1 | 0 | 0 | 0 | 0 | 0 | 0 | 0 | 0 | 0 | 0 | 0 | 0 | 1 | 0 | 0 | 0 | 1 | 0 | 0 | 0 | 0 | 0 | 0 |
| CV627 | 22 | 1 | 0 | 1 | 0 | 0 | 0 | 0 | 0 | 0 | 0 | 0 | 0 | 0 | 1 | 0 | 0 | 0 | 1 | 0 | 0 | 0 | 0 | 0 | 0 |
| CV648 | 22 | 1 | 0 | 0 | 0 | 0 | 0 | 0 | 0 | 0 | 0 | 0 | 0 | 0 | 1 | 0 | 0 | 0 | 1 | 0 | 0 | 0 | 0 | 0 | 0 |
| CV16 | 25 | 1 | 0 | 0 | 0 | 0 | 0 | 0 | 0 | 0 | 0 | 1 | 0 | 0 | 0 | 0 | 0 | 0 | 0 | 0 | 0 | 1 | 1 | 0 | 0 |
| CV168 | 25 | 1 | 0 | 0 | 0 | 0 | 0 | 0 | 0 | 1 | 0 | 1 | 0 | 0 | 0 | 0 | 0 | 0 | 0 | 0 | 0 | 1 | 0 | 0 | 0 |
| CV348 | 25 | 1 | 0 | 0 | 0 | 0 | 0 | 0 | 0 | 1 | 0 | 1 | 0 | 0 | 0 | 0 | 0 | 0 | 0 | 0 | 0 | 1 | 0 | 0 | 0 |
| CV11 | 30 | 1 | 1 | 0 | 0 | 0 | 0 | 1 | 0 | 0 | 0 | 0 | 0 | 0 | 0 | 0 | 0 | 0 | 1 | 0 | 0 | 0 | 1 | 0 | 0 |
| CV115 | 30 | 1 | 0 | 0 | 0 | 0 | 1 | 0 | 0 | 0 | 0 | 0 | 1 | 0 | 0 | 0 | 0 | 0 | 1 | 0 | 0 | 0 | 0 | 0 | 0 |
| CV120 | 30 | 1 | 0 | 0 | 0 | 0 | 0 | 1 | 0 | 0 | 0 | 0 | 0 | 0 | 0 | 0 | 0 | 0 | 1 | 0 | 0 | 0 | 1 | 0 | 0 |
| CV169 | 45 | 1 | 0 | 0 | 0 | 0 | 0 | 1 | 0 | 0 | 0 | 0 | 0 | 0 | 0 | 0 | 0 | 0 | 1 | 0 | 1 | 0 | 0 | 0 | 1 |
| CV18 | 45 | 1 | 0 | 0 | 0 | 0 | 0 | 1 | 0 | 0 | 0 | 0 | 0 | 0 | 0 | 1 | 0 | 0 | 1 | 0 | 0 | 0 | 0 | 0 | 1 |
| CV532 | 45 | 1 | 0 | 0 | 0 | 0 | 0 | 0 | 1 | 0 | 0 | 0 | 0 | 0 | 0 | 1 | 0 | 0 | 0 | 0 | 1 | 0 | 0 | 0 | 1 |
| CV591A | 45 | 1 | 0 | 0 | 0 | 0 | 0 | 0 | 0 | 0 | 0 | 0 | 0 | 0 | 0 | 1 | 0 | 0 | 0 | 0 | 0 | 0 | 0 | 0 | 0 |
| CV188 | 88 | 1 | 0 | 0 | 0 | 0 | 0 | 0 | 0 | 0 | 0 | 0 | 0 | 0 | 1 | 0 | 0 | 0 | 1 | 0 | 0 | 0 | 0 | 0 | 0 |
| CV346 | 88 | 1 | 0 | 0 | 0 | 0 | 0 | 0 | 0 | 0 | 0 | 0 | 0 | 0 | 1 | 0 | 0 | 0 | 1 | 0 | 0 | 0 | 0 | 0 | 0 |
| CV185A | 97 | 1 | 0 | 0 | 0 | 0 | 0 | 0 | 0 | 0 | 0 | 0 | 0 | 0 | 1 | 0 | 0 | 0 | 0 | 0 | 0 | 0 | 0 | 0 | 0 |
| CV390A | 97 | 1 | 0 | 0 | 0 | 0 | 0 | 0 | 0 | 0 | 0 | 0 | 0 | 0 | 1 | 0 | 0 | 0 | 0 | 0 | 0 | 0 | 0 | 0 | 0 |
| CV603 | 97 | 1 | 0 | 0 | 0 | 0 | 0 | 0 | 0 | 0 | 0 | 0 | 0 | 0 | 1 | 0 | 0 | 0 | 0 | 0 | 0 | 0 | 0 | 0 | 0 |
| CV109 | 121 | 1 | 0 | 0 | 0 | 0 | 0 | 0 | 0 | 0 | 0 | 0 | 0 | 0 | 0 | 0 | 0 | 0 | 0 | 0 | 0 | 0 | 1 | 0 | 0 |
| CV133 | 121 | 1 | 0 | 0 | 0 | 0 | 0 | 0 | 0 | 0 | 0 | 0 | 0 | 0 | 0 | 0 | 0 | 0 | 0 | 0 | 0 | 0 | 0 | 0 | 0 |
| CV15 | 121 | 1 | 0 | 0 | 0 | 0 | 0 | 0 | 0 | 0 | 0 | 0 | 0 | 0 | 0 | 0 | 1 | 0 | 0 | 0 | 0 | 0 | 0 | 0 | 0 |
| CV233 | 121 | 1 | 0 | 0 | 0 | 0 | 0 | 0 | 0 | 0 | 0 | 0 | 0 | 0 | 0 | 0 | 0 | 0 | 0 | 0 | 0 | 0 | 0 | 0 | 0 |
| CV540 | 273 | 1 | 0 | 0 | 0 | 0 | 0 | 0 | 0 | 0 | 0 | 0 | 0 | 0 | 0 | 0 | 0 | 0 | 0 | 1 | 0 | 0 | 0 | 0 | 0 |
| CV625A | 273 | 1 | 0 | 0 | 0 | 0 | 0 | 0 | 0 | 0 | 0 | 0 | 0 | 0 | 0 | 0 | 0 | 0 | 0 | 1 | 0 | 0 | 0 | 0 | 0 |
| CV55 | 291 | 1 | 0 | 0 | 0 | 0 | 0 | 1 | 0 | 0 | 0 | 0 | 0 | 0 | 0 | 0 | 0 | 0 | 1 | 0 | 0 | 0 | 0 | 0 | 0 |
| CV464 | 398 | 1 | 0 | 0 | 1 | 0 | 0 | 0 | 0 | 0 | 0 | 0 | 0 | 0 | 0 | 0 | 0 | 0 | 0 | 0 | 0 | 0 | 0 | 0 | 0 |
| CV174 | 508 | 1 | 0 | 0 | 0 | 0 | 0 | 1 | 0 | 0 | 0 | 0 | 0 | 0 | 0 | 0 | 0 | 0 | 1 | 0 | 1 | 0 | 0 | 0 | 0 |
| CV220A | 508 | 1 | 0 | 0 | 0 | 0 | 0 | 1 | 0 | 0 | 0 | 0 | 0 | 0 | 0 | 1 | 0 | 0 | 1 | 0 | 1 | 0 | 0 | 0 | 0 |
| CV314A | 508 | 1 | 0 | 0 | 0 | 0 | 0 | 1 | 0 | 0 | 0 | 0 | 0 | 0 | 0 | 0 | 0 | 0 | 1 | 0 | 0 | 0 | 0 | 0 | 0 |
| CV356 | 508 | 1 | 0 | 0 | 1 | 0 | 0 | 0 | 1 | 0 | 0 | 0 | 0 | 0 | 0 | 0 | 0 | 0 | 1 | 0 | 1 | 0 | 0 | 0 | 1 |
| CV573 | 508 | 1 | 0 | 0 | 0 | 0 | 0 | 1 | 0 | 0 | 0 | 0 | 0 | 0 | 0 | 1 | 0 | 0 | 1 | 0 | 1 | 0 | 0 | 0 | 0 |
| CV587 | 508 | 1 | 0 | 0 | 0 | 0 | 0 | 1 | 0 | 0 | 0 | 0 | 0 | 0 | 0 | 0 | 0 | 0 | 1 | 0 | 1 | 0 | 0 | 0 | 0 |
| CV638 | 508 | 1 | 0 | 0 | 0 | 0 | 0 | 1 | 0 | 0 | 0 | 0 | 0 | 0 | 0 | 0 | 0 | 0 | 1 | 0 | 1 | 0 | 0 | 0 | 0 |
| CV81 | 508 | 1 | 0 | 0 | 0 | 0 | 0 | 1 | 0 | 0 | 0 | 0 | 0 | 0 | 0 | 1 | 0 | 0 | 1 | 0 | 1 | 0 | 0 | 0 | 0 |
| CV87 | 668 | 1 | 0 | 0 | 0 | 0 | 0 | 0 | 0 | 0 | 0 | 0 | 0 | 0 | 1 | 0 | 0 | 0 | 0 | 0 | 0 | 0 | 0 | 0 | 0 |
| CV107 | 669 | 1 | 0 | 0 | 0 | 0 | 0 | 0 | 0 | 0 | 0 | 0 | 0 | 0 | 1 | 0 | 0 | 0 | 0 | 0 | 0 | 0 | 0 | 0 | 0 |
| CV110 | 669 | 1 | 0 | 0 | 0 | 0 | 0 | 0 | 0 | 0 | 0 | 0 | 0 | 0 | 1 | 0 | 0 | 0 | 0 | 0 | 0 | 0 | 0 | 0 | 0 |
| CV145 | 669 | 1 | 0 | 0 | 0 | 0 | 0 | 0 | 0 | 0 | 0 | 0 | 0 | 0 | 1 | 0 | 0 | 0 | 0 | 0 | 0 | 0 | 0 | 0 | 0 |
| CV161 | 669 | 1 | 0 | 0 | 0 | 0 | 0 | 0 | 0 | 0 | 0 | 0 | 0 | 0 | 1 | 0 | 0 | 0 | 0 | 0 | 0 | 0 | 1 | 0 | 0 |
| CV173 | 669 | 1 | 0 | 0 | 0 | 0 | 0 | 0 | 0 | 0 | 0 | 0 | 0 | 0 | 1 | 0 | 0 | 0 | 0 | 0 | 0 | 0 | 0 | 0 | 0 |
| CV361A | 669 | 1 | 0 | 0 | 0 | 0 | 0 | 0 | 0 | 0 | 0 | 0 | 0 | 0 | 0 | 0 | 0 | 0 | 0 | 0 | 0 | 0 | 0 | 0 | 0 |
| CV85 | 669 | 1 | 0 | 0 | 0 | 0 | 0 | 0 | 0 | 0 | 0 | 0 | 0 | 0 | 1 | 0 | 0 | 0 | 0 | 0 | 0 | 0 | 0 | 0 | 0 |
| CV34 | 1472 | 1 | 0 | 0 | 0 | 0 | 0 | 0 | 0 | 0 | 0 | 0 | 0 | 0 | 1 | 0 | 0 | 0 | 0 | 0 | 0 | 0 | 0 | 0 | 0 |
| CV442 | 1472 | 1 | 0 | 0 | 0 | 0 | 0 | 0 | 0 | 0 | 0 | 0 | 0 | 0 | 1 | 0 | 0 | 0 | 0 | 0 | 1 | 0 | 0 | 0 | 0 |
| CV462 | 1472 | 1 | 0 | 0 | 0 | 0 | 0 | 0 | 0 | 0 | 0 | 0 | 0 | 0 | 1 | 0 | 0 | 0 | 0 | 0 | 0 | 0 | 0 | 0 | 0 |
| CV496 | 1472 | 1 | 0 | 0 | 0 | 0 | 0 | 0 | 0 | 0 | 0 | 0 | 0 | 0 | 1 | 0 | 0 | 0 | 0 | 0 | 0 | 0 | 0 | 0 | 0 |
| CV558 | 1472 | 1 | 0 | 0 | 0 | 0 | 0 | 0 | 0 | 0 | 0 | 0 | 0 | 0 | 1 | 0 | 0 | 0 | 0 | 0 | 0 | 0 | 0 | 0 | 0 |
| CV58 | 2021 | 1 | 0 | 0 | 0 | 0 | 0 | 0 | 0 | 0 | 0 | 0 | 0 | 0 | 1 | 0 | 0 | 0 | 0 | 0 | 0 | 1 | 0 | 0 | 0 |
| CV151 | 2300 | 1 | 1 | 0 | 0 | 0 | 0 | 0 | 0 | 0 | 0 | 0 | 0 | 0 | 1 | 0 | 0 | 0 | 0 | 0 | 0 | 1 | 0 | 0 | 0 |
| CV476 | 2498 | 1 | 0 | 0 | 0 | 0 | 0 | 1 | 0 | 0 | 0 | 0 | 0 | 0 | 0 | 0 | 0 | 0 | 1 | 0 | 1 | 0 | 0 | 0 | 0 |
| CV531A | 4996 | 1 | 0 | 0 | 0 | 0 | 0 | 0 | 0 | 0 | 0 | 0 | 0 | 0 | 0 | 0 | 0 | 0 | 0 | 0 | 0 | 0 | 0 | 0 | 0 |
| CV144 | 5980 | 1 | 0 | 0 | 0 | 0 | 1 | 0 | 0 | 0 | 1 | 0 | 0 | 1 | 0 | 0 | 0 | 1 | 0 | 0 | 0 | 0 | 0 | 1 | 0 |
| CV225 | 5981 | 1 | 0 | 0 | 0 | 0 | 0 | 1 | 0 | 0 | 0 | 0 | 0 | 0 | 0 | 1 | 0 | 0 | 1 | 0 | 1 | 0 | 0 | 0 | 0 |

Table S6: List of *S. aureus* genomes used for comparative analysis

| **Strain ID** | **Isolation Country** | **ST** | **BioProject** | **BioSample** | **Collection Date** | **Assembly** | **GenBank** |
| --- | --- | --- | --- | --- | --- | --- | --- |
| W24B_090215 | Ghana | 152 | PRJNA295807 | SAMN04090008 | 2015 | GCA_002905515.1 | LKTK01000000 |
| GHA/LAMRSA/2016/12 | Ghana | 152 | PRJNA588370 | SAMN13242392 | 2016 |  | WJTV01000000 |
| GHA/LAMRSA/2016/10 | Ghana | 152 | PRJNA588370 | SAMN13242390 | 2016 |  | WJTX01000000 |
| GHA/LAMRSA/2016/3 | Ghana | 152 | PRJNA588370 | SAMN13242383 | 2016 |  | WJUJ01000000 |
| SA23KEN | Kenya | 152 | PRJNA481322 | SAMN09665675 | 2015 |  | QPLA01000000 |
| SA20KEN | Kenya | 152 | PRJNA481322 | SAMN09665672 | 2015 |  | QPLC01000000 |
| SA14KEN | Kenya | 152 | PRJNA481322 | SAMN09665666 | 2015 |  | QPLH01000000 |
| SA18KEN | Kenya | 152 | PRJNA481322 | SAMN09665670 | 2015 |  | QPLE01000000 |
| BU_G0301_t8 | Ghana | 152 | PRJNA283747 | SAMN03658603 | 2013 | GCF_001297465.1 | LFOG01000000 |
| BU_G1101_t2 | Ghana | 152 | PRJNA283747 | SAMN03658605 | 2013 | GCF_001297625.1 | LFTU01000000 |
| BU_N17W_t2 | Ghana | 152 | PRJNA283747 | SAMN03658607 | 2013 | GCF_001297635.1 | LFTV01000000 |
| 04Hi | Tanzania | 152 | PRJEB2655 | SAMEA3109324 | 2014 | GCF_900097465.1 | FMNR00000000 |
| 011Hii | Tanzania | 152 | PRJEB2655 | SAMEA3109319 | 2014 | GCF_900097565.1 | FMMU00000000 |
| 3688STDY6125027 | Thailand | 188 | PRJEB9575 | SAMEA3449035 | 2015 | GCF_900125165.1 | FQEW00000000 |
| 3688STDY6124974 | Thailand | 188 | PRJEB9575 | SAMEA3448984 | 2015 | GCF_900124685.1 | FQDC00000000 |
| HU-85a | Argentina | 188 | PRJNA414566 | SAMN07782090 | 2005 | GCA_002930565.1 | PKCG01000000 |
| SGY37 | China | 188 | PRJNA433074 | SAMN08470413 | 2016 | GCA_003309205.1 | PTAA01000000 |
| S1AH041 | China | 188 | PRJNA433074 | SAMN08470384 | 2015 | GCA_003309645.1 | PTBD01000000 |
| S1AG056 | China | 188 | PRJNA433074 | SAMN08470382 | 2015 | GCA_003309655.1 | PTBF01000000 |
| S1AD014 | China | 188 | PRJNA433074 | SAMN08470380 | 2015 | GCA_003309705.1 | PTBH01000000 |
| S1AK033 | China | 188 | PRJNA433074 | SAMN08470391 | 2015 | GCA_003309935.1 | PTAW01000000 |
| YNSA504 | China | 188 | PRJNA543691 | SAMN11775323 | 2014 | GCA_005860885.1 | VCGR01000000 |
| YNSA5 | China | 188 | PRJNA543691 | SAMN11775265 | 2014 | GCA_005861625.1 | VCEL01000000 |
| 5230 | Colombia | 188 | PRJNA595347 | SAMN13979032 | 2018 |  |  |
| 5144 | Colombia | 188 | PRJNA595347 | SAMN13979019 | 2018 |  |  |
| 5321 | Colombia | 188 | PRJNA595347 | SAMN13979043 | 2018 |  |  |
| CUHK_HK188 | China | 188 | PRJNA239406 | SAMN02665331 | 2007 | GCF_000590395.1 | JFFV01000000 |
| HPV107 | Portugal | 5 | PRJNA231221 | SAMN03255486 | 1992 | GCF_001019575.1 | JYAY01000000 |
| HDE288 | Portugal | 5 | PRJNA231221 | SAMN03255441 | 1996 | GCF_001019255.1 | JXZF01000000 |
| HDE288 | Portugal | 5 | PRJNA231221 | SAMN03255487 | 1996 | GCF_001019205.1 | JYAZ01000000 |
| N34_260215 | Ghana | 5 | PRJNA295807 | SAMN04089992 | 2015 | GCA_002905355.1 | LKSW01000000 |
| BU_W7A_t11 | Ghana | 5 | PRJNA283747 | SAMN03658594 | 2013 | GCF_001297285.1 | LFNL01000000 |
| BU_W22_t4 | Ghana | 5 | PRJNA283747 | SAMN03658593 | 2013 | GCF_001297525.1 | LFNK01000000 |
| C80 | Brazil | 5 | PRJNA355562 | SAMN06077115 | 2012 | GCF_001921695.1 | MSFE00000000 |
| UB590 | Brazil | 5 | PRJNA291213 | SAMN03940752 | 2012 | GCA_002267645.1 | LGWU00000000 |
| UB594 | Brazil | 5 | PRJNA291213 | SAMN03940753 | 2012 | GCA_002267705.1 | LGWC00000000 |
| UB670 | Brazil | 5 | PRJNA291213 | SAMN03944932 | 2012 | GCA_002267965.1 | LGYL00000000 |
| C6 | Brazil | 5 | PRJNA262826 | SAMN03766024 | 2014 | GCA_002734415.1 | LELV01000000 |
| A5 | Brazil | 5 | PRJNA262826 | SAMN03766006 | 2014 | GCA_002734455.1 | LELM01000000 |
| C7 | Brazil | 5 | PRJNA262826 | SAMN03766025 | 2014 | GCA_002734425.1 | LELW01000000 |
| B7 | Brazil | 5 | PRJNA262826 | SAMN03766020 | 2014 | GCA_002734485.1 | LELR01000000 |
| SA_70002 | Spain | 72 | PRJNA345163 | SAMN05858838 | 2014 | GCF_001900005.1 | MKZD00000000 |
| SA_190006 | Spain | 72 | PRJNA345163 | SAMN05860691 | 2013 | GCF_001900165.1 | MKZK00000000 |
| CFSA157 | United States | 72 | PRJNA380429 | SAMN06698115 | 2014 | GCA_002123615.1 | NDQX00000000 |
| M3140 | Denmark | 72 | PRJEB24452 | SAMEA104473463 | 2014 | GCA_900251275.1 | OFYF01000000 |
| H1356 | Denmark | 72 | PRJEB24452 | SAMEA104473371 | 2014 | GCA_900250315.1 | OFUN01000000 |
| BCH-SA-03 | United States | 72 | PRJNA480016 | SAMN09847806 | 2018 | GCA_003721095.1 | RIWJ01000000 |
| MSSA | United States | 72 | PRJEB1915 | SAMEA2384046 | 2009 | GCF_900080955.1 | FKOI00000000 |
| M0139 | United States | 72 | PRJNA216717 | SAMN02325668 | 2003 | GCA_000531015.1 | JAZL00000000 |
